# Hydrogen-Bonding Interactions Trigger a Spin-Flip in Iron(III) Porphyrin Complexes**

**DOI:** 10.1002/anie.201411399

**Published:** 2015-01-30

**Authors:** Dipankar Sahoo, Matthew G Quesne, Sam P de Visser, Sankar Prasad Rath

**Affiliations:** Department of Chemistry, Indian Institute of Technology Kanpur Kanpur-208016 (India) E-mail: sprath@iitk.ac.in; Manchester Institute of Biotechnology and School of Chemical Engineering and Analytical Science, The University of Manchester 131 Princess Street, Manchester M1 7DN (UK) E-mail: sam.devisser@manchester.ac.uk

**Keywords:** electronic structure, hydrogen bonding, porphyrinoids, spin crossover, structure elucidation

## Abstract

A key step in cytochrome P450 catalysis includes the spin-state crossing from low spin to high spin upon substrate binding and subsequent reduction of the heme. Clearly, a weak perturbation in P450 enzymes triggers a spin-state crossing. However, the origin of the process whereby enzymes reorganize their active site through external perturbations, such as hydrogen bonding, is still poorly understood. We have thus studied the impact of hydrogen-bonding interactions on the electronic structure of a five-coordinate iron(III) octaethyltetraarylporphyrin chloride. The spin state of the metal was found to switch reversibly between high (S=^5^/_2_) and intermediate spin (S=^3^/_2_) with hydrogen bonding. Our study highlights the possible effects and importance of hydrogen-bonding interactions in heme proteins. This is the first example of a synthetic iron(III) complex that can reversibly change its spin state between a high and an intermediate state through weak external perturbations.

Heme proteins carry out a wide range of physiological functions with the same prosthetic group, the iron derivative of protoporphyrin IX, which has continued to fascinate and perplex. A common feature in many of these systems, such as hemoglobin, cytochrome P450, and horseradish peroxidase, is the appearance of hydrogen bonds in the secondary coordination sphere which are assumed to control the metal-mediated processes.[1–3] Furthermore, structural studies have implicated active-site hydrogen-bonding networks in the function of various non-heme proteins.[4, 5] There has been substantial speculation that hydrogen bonding in heme/non-heme proteins could control the relative stability of the oxidation/spin state of the iron center and thereby control the electronic structure and reactivity.[2–6] However, the fundamental aspects of how hydrogen bonds facilitate most of these processes are still largely unknown. We report here the synthesis, structure, and property of a five-coordinate iron(III) octaethyltetraarylporphyrin chloride whose metal spin can be switched reversibly by hydrogen bonding between a high (*S*=^5^/_2_) and an intermediate spin (*S*=^3^/_2_) state.

Iron(III) octaethyltetraarylporphyrin chloride **1** was synthesized by the insertion of iron into the free base porphyrin in *N*,*N*-dimethylformamide (Scheme 1). Dark-purple crystals of the molecule were grown by slow diffusion of cyclohexane into a solution of the complex in chloroform at room temperature. The addition of phenol (4–5 molar excess) to the chloroform solution of **1** and subsequent slow diffusion with *n*-hexane at room temperature in air yields dark purple crystals of iron(III) octaethyltetraarylporphyrin chloride **2**, in which the axial chloride is engaged in hydrogen-bonding interactions with the unbound phenol. Similarly, the addition of 2-isopropylaniline or aniline into the dichloromethane solution of **1** also yields dark purple crystals of iron(III) octaethyltetraarylporphyrin chloride **3** and **4**, respectively, where the axial chloride is engaged in hydrogen-bonding interactions with 2-isopropylaniline or aniline.

**Scheme 1:**
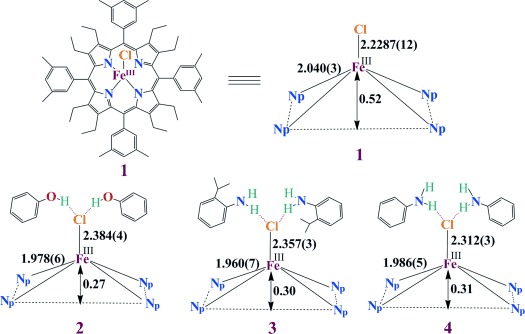
Schematic representation of the complexes and their abbreviations, with distances as obtained from the crystal structures.

All the complexes reported here were isolated as a crystalline solid in good yields and structurally characterized.[7] Perspective views of the complexes are shown in Figure 1 (for **1**–**3**; see Figure S1 in the Supporting Information for **4** and Figures S2–S5 for the molecular packing diagrams). In the X-ray structure of the complexes (Tables S1 and S2 in the Supporting Information), the iron centers have a five-coordinate square-pyramidal geometry and show considerable doming of the porphyrin cores and out-of-plane displacement of the metal ions. The Fe–Cl bond undergoes an extensive elongation from a distance of 2.2287(12) Å in **1** to 2.384(4), 2.357(3), and 2.312(3) Å in the hydrogen-bonded complexes **2**, **3**, and **4**, respectively (see Table S2 in the Supporting Information). Also, the average Fe–N_p_ distances in the two types of complexes are very different: 2.040(3) Å in **1** and 1.978(6), 1.960(7), and 1.986(5) Å in **2**, **3**, and **4**, respectively. The displacement of the iron center from the mean plane of the C_20_N_4_ porphyrinato core (▵) is contracted from 0.52 Å (in **1**) to 0.27, 0.30, and 0.31 Å in **2, 3**, and **4**, respectively. All these structural features are characteristic of Fe having a high-spin (*S*=^5^/_2_) nature in **1** but intermediate spin (*S*=^3^/_2_) states in **2**, **3**, and **4**.[8–11] As can be seen, the Fe-N_p_ and Δ distances in **2**, **3**, and **4** are much shorter than in **1**, whereas the Fe–Cl distance is much longer in the series (see Table S3 in the Supporting Information, which compares selected structural parameters of the complexes reported here along with similarly distorted iron(III) porphyrin chloride reported earlier). The proton on the phenolic oxygen atom was directly located in the difference Fourier maps with an O–H distance of 0.94(3) Å and is found to be engaged in hydrogen-bonding interactions with the chlorine atom in **2** [O1⋅⋅⋅Cl1=3.100(8) Å]. Similar hydrogen-bonding interactions are also observed in **3** and **4**, with N1L⋅⋅⋅Cl1=3.262(7) [3.318(7)] Å, and N2L⋅⋅⋅Cl1=3.021(9) Å. As can be seen, the Fe–Cl1 distance increases as the strength of the hydrogen bonding increases. The presence of two such hydrogen-bonding interactions between phenol, 2-isopropylaniline, or aniline and the axial chloride ligand in **2**, **3**, and **4**, respectively, results in an unusual lengthening of the Fe–Cl bond, which eventually shortens the Fe–N_p_ bond and the Δ distances and thus leads to a natural way of stabilizing the intermediate spin state of iron. The UV/Vis spectrum of **1** shows a split Soret band at 401 and 443 nm, while the hydrogen-bonding interactions in **2** and **3** leads to a small blue-shift of the Soret band to *λ*_max_=440 and 437 nm, respectively. Figure 2 compares the UV/Vis spectra of **1**, **2**, and **3** in benzene.

**Figure 1:**
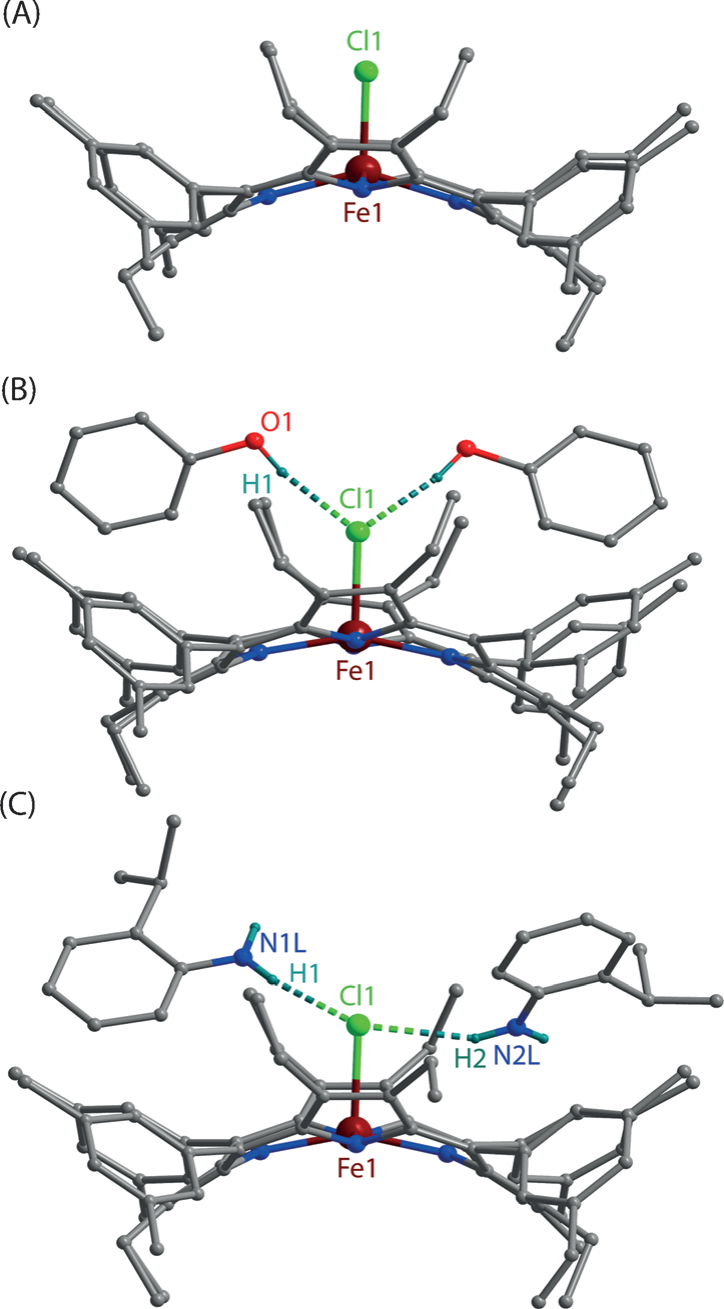
Molecular structures (at 100 K) of (A) 1, (B) 2, and (C) 3 (all the H atoms except for the phenol/amine have been omitted for clarity). Selected bond distances [Å] and angle (°) for 1: Fe1-N1 2.040(3), Fe1-N2 2.044(3), Fe1-N3 2.031(3), Fe1-N4 2.046(3), Fe1-Cl1 2.2287(12); N1-Fe1-N2 87.05(12), N1-Fe1-N4 87.17(12), N2-Fe1-N4 145.46(12), N3-Fe1-N1 159.09(12), N3-Fe1-N2 86.61(12), N3-Fe1-N4 86.82(11), N1-Fe1-Cl1 100.26(9), N2-Fe1-Cl1 107.27(9), N3-Fe1-Cl1 100.66(9), N4-Fe1-Cl1 107.27(9). For 2: Fe1-N1 1.997(6), Fe1-N2 1.960(5), Fe1-Cl1 2.384(4); N1-Fe1-N2 89.5(2), N1-Fe1-Cl1 102.3(2), N2-Fe1-Cl1 94.6(2). Hydrogen-bonding interactions: O1⋅⋅⋅Cl1 3.100(8), O2⋅⋅⋅O1 2.923(14). For 3: Fe1-N1 1.965(7), Fe1-N2 1.970(7), Fe1-N3 1.937(7), Fe1-N4 1.968(7), Fe1-Cl1 2.357(3); N1-Fe1-N2 89.2(3), N1-Fe1-N4 88.5(3), N2-Fe1-N4 155.7(3), N3-Fe1-N1 169.9(3), N3-Fe1-N2 87.9(3), N3-Fe1-N4 90.2(3), N1-Fe1-Cl1 95.6(2), N2-Fe1-Cl1 100.8(2), N3-Fe1-Cl1 94.4(2), N4-Fe1-Cl1 103.5(2). Hydrogen-bonding interactions: N1L⋅⋅⋅Cl1 3.262(7), N2L⋅⋅⋅Cl1 3.021(9).

**Figure 2:**
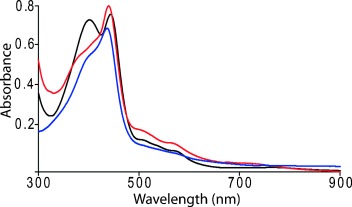
UV/Vis (in C_6_H_6_ at 295 K) spectra using polycrystalline samples of 1 (black), 2 (red), and 3 (blue).

The porphyrin rings are highly distorted in the complexes reported here and are best appreciated by comparing the out-of-plane displacements of the porphyrin core atoms in **1** and **2** (see Figure S6 in the Supporting Information). The porphyrin ring is distorted in a saddle shape, with the two complexes showing nearly identical atom deviations from the mean porphyrin plane.

Mössbauer parameters are one of the most powerful probes to determine the spin states of the iron(III) porphyrins and Figure 3 compares the spectra of microcrystalline samples of **1** and **2** at 295 K.[8, 9, 11] Complex **1** shows a small quadrupole splitting [*δ* (Δ*E*_q_): 0.28 (0.88) mm s^−1^] which falls within the range of the parameters known for high-spin (*S*=^5^/_2_) Fe^III^porphyrins, while **2** exhibits large quadrupole-split doublets [*δ* (Δ*E*_q_): 0.29 (3.56) mm s^−1^] characteristic of iron in an intermediate spin (*S*=^3^/_2_) state.

**Figure 3:**
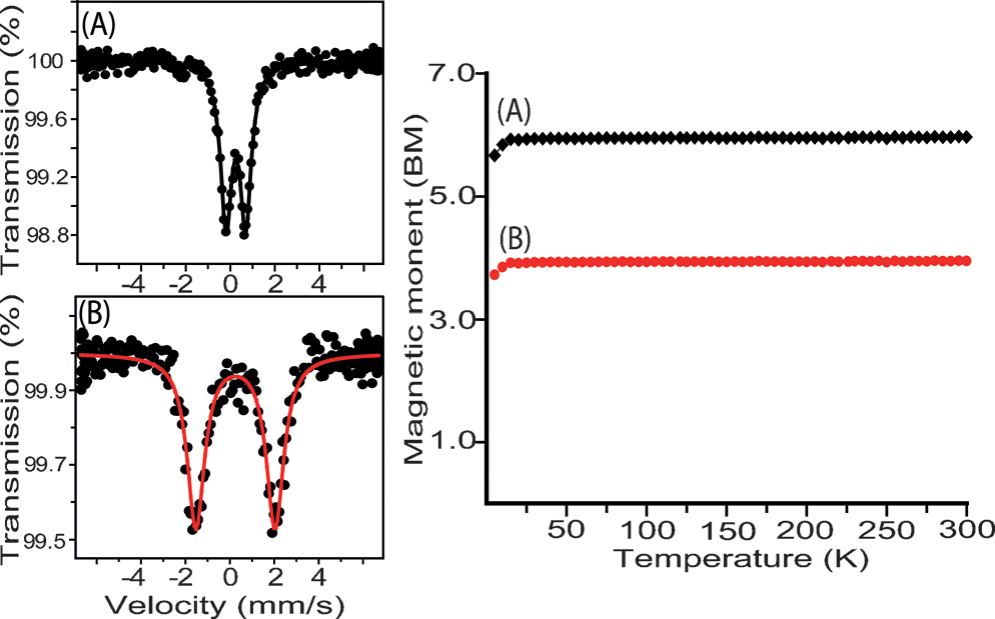
Left: Mössbauer spectra at 295 K and right: plot of magnetic moment versus temperature (K) of the microcrystalline samples of A) 1 and B) 2.

The magnetic moments of the microcrystalline samples of **1** and **2** are found to be substantially independent of temperature over a temperature range of 5 to 300 K. Figure 3 also shows the plot of the effective magnetic moment against the temperature. A value of 5.86 and 3.85 μB per iron at 295 K have been observed for **1** and **2**, respectively, which further confirm the presence of the high (*S*=^5^/_2_) and intermediate spins (S=^3^/_2_) of iron in the complexes. The EPR spectroscopic measurements were carried out for the complexes in both the solid and solution phase (see Figure S7 in the Supporting Information); all the spectra are axially symmetric with *g*_⊥_=5.78 and *g*_∥_=1.99 for **1**, *g*_⊥_=4.16 and *g*_∥_=2.01 for **2**, and *g*_⊥_=4.19 and *g*_∥_=1.96 for **3** at 77 K in the solid state. Similar *g* values are also obtained for the molecules in solution (see the Experimental Section). These results provide unequivocal evidence of the high-spin Fe^III^ (*S*=^5^/_2_) nature of complex **1** whereas **2** and **3** show the signature of an intermediate spin (*S*=^3^/_2_) state, in both the solid and solution phase.

^1^H NMR spectroscopy is a valuable tool to distinguish different spin states of iron(III) porphyrins in solution;[8–11] Figure 4 shows the spectra of **1** and **2** recorded at 298 K in CDCl_3_. For complex **1**, four methylene proton signals are observed at 45.43, 37.65, 34.47, and 23.52 ppm which are characteristic of high-spin iron. In complex **2**, however, four methylene peaks (at 49.29, 24.93, 24.13, and 13.13 ppm) are found in a relatively wider region, and the signal positions are very similar to those of the reported five-coordinate iron(III) porphyrins with pure intermediate spin states.[11a] Similarly, the ^1^H NMR spectrum has also been recorded for **3** in CDCl_3_ (see Figure S8 in the Supporting Information), which gives similar spectral features as **2**. On the basis of the above results, the Fe^III^ centers in **2** and **3** are assigned to be in the intermediate state, as also observed in the solid state. The temperature dependence of the signals almost follow the Curie law (see Figure S9 in the Supporting Information for **2**), which is indicative of a single spin state for the complex over the temperature range.[8, 9] Moreover, stepwise additions of phenol to **1** in CDCl_3_ resulted in the ^1^H NMR spectra changing dramatically, with one methylene signal shifting downfield, while the three others move more upfield. Similar spectral changes are also obtained during titration with 2-isopropylaniline. The ^1^H NMR spectra, therefore, clearly demonstrate the effect of hydrogen bonding in solution. It is interesting to note here that the addition of a large excess of phenol to **1** in chloroform and subsequent reflux for 30 min, however, produces five-coordinate phenolato Fe^III^ porphyrin, which was also isolated as a solid and characterized to have only a high-spin iron center (see Figures S8 and S10 in the Supporting Information).

**Figure 4:**
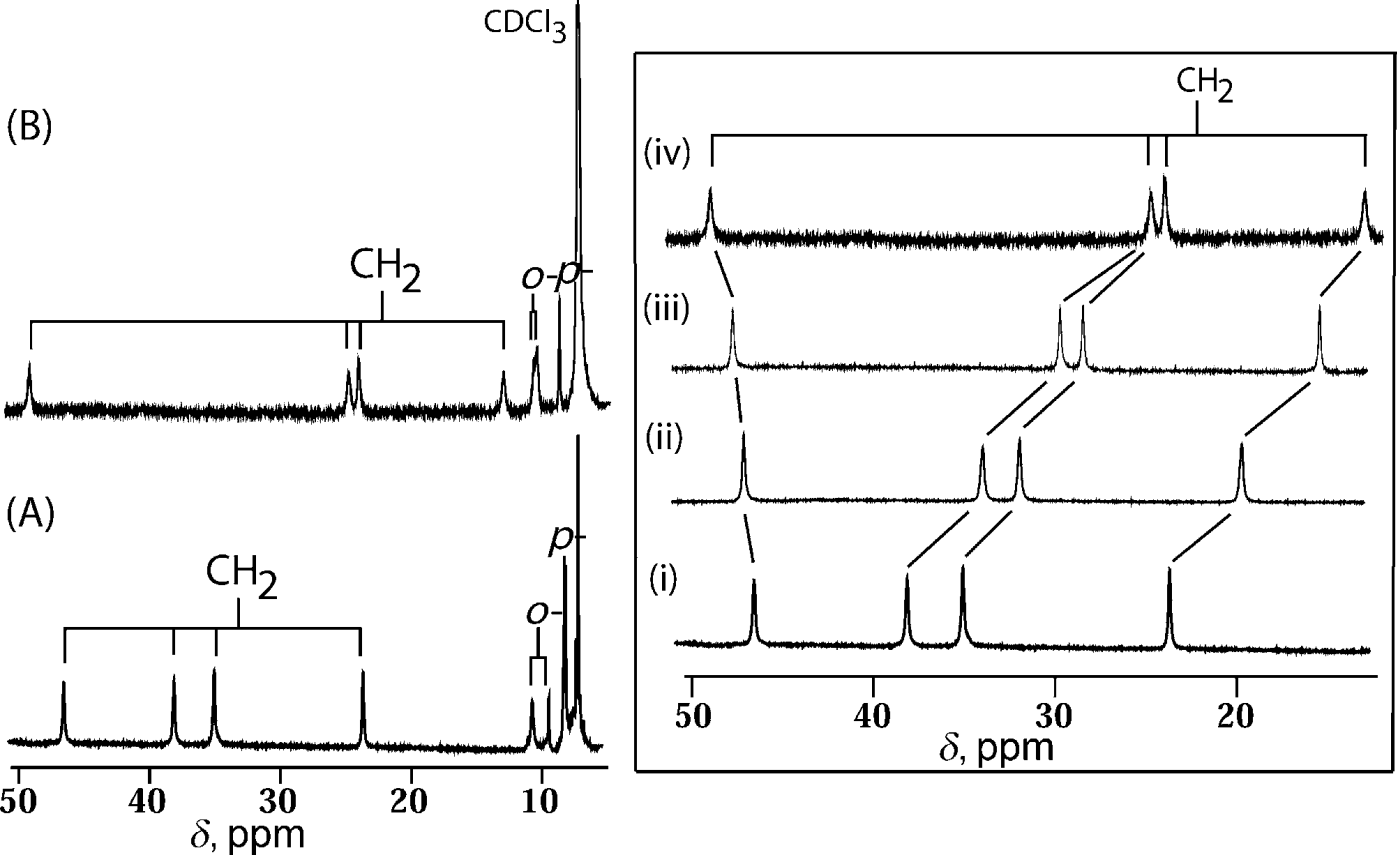
^1^H NMR (at 295 K in CDCl_3_) spectra of A) 1 and B) 2 using crystalline samples. Right: ^1^H NMR spectral changes (methylene signals only) of 1 (ca. 10^−3^ m) in CDCl_3_ at 295 K upon gradual addition of phenol at the molar ratio of i) 1:0, ii) 1:3, iii) 1:5, and iv) solution after 6 h.

To gain insight into the origin of the spin-state reversal, we ran a series of density functional theory calculations on models **1**–**4**, using a selection of different density functional theory methods and basis sets (See the Supporting Information for details). The optimized geometries are given in Figure 5. Thus, the system without any hydrogen-bonding interactions remains in a high-spin state that is Δ*G*_SQ_=2.3 kcal mol^−1^ below the quartet spin state. In contrast, the addition of two hydrogen-bonded phenol or aniline molecules changes the spin-state ordering and makes the quartet spin state the ground state by Δ*G*_SQ_=6.9 and 1.3 kcal mol^−1^, respectively. In the gas phase, ^4^**1** is lower in energy than ^6^**1**, and only the addition of the entropic, thermal, and dispersion corrections lowers ^6^**1** below ^4^**1**. The optimized geometries of ^6^**1**, ^4^**2**, and ^4^**4** match the crystal structure coordinates reported in Scheme 1 reasonably well and have very similar bond distances. In general, the calculated bond lengths between second-row elements, such as Fe–S or Fe–Cl distances, have a deviation from experiment of the order of about 0.10 Å, and most distances are well within this range.[12] Thus, the Fe–Cl bond elongates from 2.29 Å in ^6^**1** to 2.48 Å in ^4^**2** because of the electron-withdrawing effect of the phenol hydrogen bonds pulling the chloride away from the iron center. At the same time, there is charge transfer (about *Q*=−0.14) from the metal to the phenol molecules. The elongation of the Fe–Cl bond as a result of the hydrogen-bonding interactions results in the energetic stabilization of the quartet spin state over the sextet spin state and the change in spin-state ordering. The hydrogen-bonding interactions are on the order of 2.1 Å in all the optimized geometries, which are typical for interactions with second-row elements, such as sulfur or chloride.[13]

**Figure 5:**
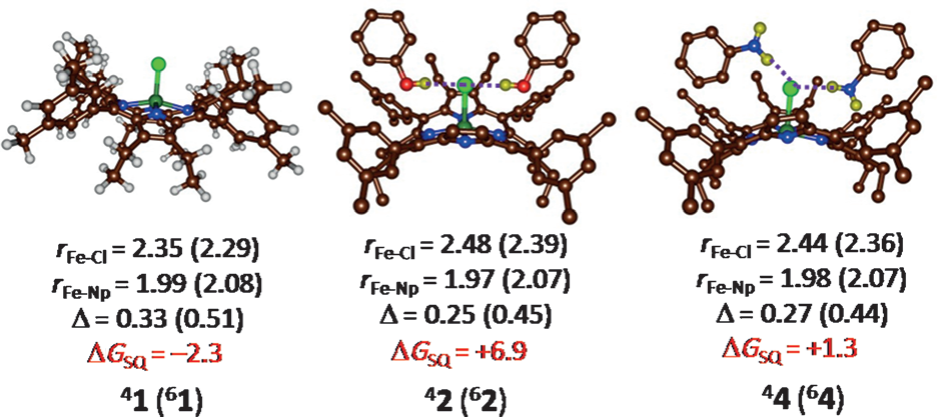
UB3LYP/BS3 optimized geometries of ^4,6^1, ^4,6^2, and ^4,6^4 with bond lengths in Å. Δ is the average displacement of the metal from the porphyrin plane. Free energies are relative to the quartet spin state in kcal mol^−1^ and include solvent, entropic, and thermal corrections to the energy.

Studies of heme models suggest that the intermediate spin state critically depends on the presence of a weak-field axial ligand, while deformation of the porphyrin ring is also known to play a significant role in controlling the spins of iron(III) porphyrinates.[8–11] Here, the porphyrin rings have identical ring deformations (see Figure S6 in the Supporting Information) for the complexes with (**2**, **3**, and **4**) or without (**1**) hydrogen-bonding interactions. Thus, the ring deformation is not responsible for the spin flip as observed earlier.[9b,d] Moreover, an axial chloride ion is generally not considered to be a sufficiently weak-field ligand that enables the stabilization of the intermediate spin of iron on its own. The lengthening of the Fe–Cl bond distances observed in the present study is ascribed to the effects of the hydrogen-bonding network, which removes electron density from the chloride ligand and causes a weakening of the metal–chloride bond. X-ray structure analysis of a series of transition-metal chloro complexes [M^II^(tao)(Cl)]^+^, where M=Cr, Mn, Fe, Co, Ni, Cu, and Zn and tao=tris(2-aminooxazoline)amine, have been reported earlier.[14a] The M^II^–Cl bond lengths were found to be longer than those found in other trigonal bipyramidal complexes because of the electron-withdrawing effects of the hydrogen-bonding network. The perturbation of spin states in iron complexes by hydrogen bonding has been seen before; however, such an abrupt change between a high and intermediate spin of a synthetic iron(III) porphyrin has been reported here for the first time.[14b–d]

To further understand the spin-state ordering change of **1** versus **2** and **4** we analyzed the relevant metal-type molecular orbitals of the complexes. Figure 6 A gives the orbital energy levels of the α and β set of orbitals for the *δ_xy_*, π*_xz_, π*_yz_, 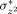, and 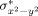 molecular orbitals for ^4,6^**1** and ^4,6^**2**. As can be seen, dramatic changes in the orbital energy levels are obtained upon hydrogen bonding (see Table S18 in the Supporting Information). Thus, the addition of two hydrogen-bonding phenol moieties stabilizes the *δ_xy_* orbital dramatically in the sextet spin state as well as the π*_xz_, π*_yz_ and 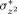 orbitals. Stabilization of the *δ_xy_* orbital leads to its double occupation and a conversion of a high spin into intermediate spin state. As shown previously,[15] when the metal moves out of the plane of the porphyrin ring, that is, there is an increased value of Δ, the π*_xz_/π*_yz_ orbitals interact with an occupied e_g_ pair of orbitals and are stabilized in energy, which is indeed what is seen here. Hydrogen-bonding interactions pull the chloride anion away from the metal, which is pushed back into the plane of the porphyrin, thereby reducing the metal displacement value Δ and thereby weakening the orbital overlap between π*_xz_/π*_yz_ and the e_g_ orbitals. Energetic lowering of the π*_xz_, π*_yz_, as well as the *δ_xy_* orbitals stabilize a quartet spin state over the sextet spin state. Particularly clear is the orbital interaction of this mixed π*_xz_/e_g_ orbital with contributions of the 2p_*x*_ orbitals on the phenolate oxygen atoms. Therefore, hydrogen-bonding interactions have a direct effect on the energy level of the π*_xz_ molecular orbital and consequently the quartet–sextet energy splitting. It should be noted here as well that hydrogen-bonding interactions to nonheme iron(IV)-oxo complexes were found to decrease the catalytic activity of the oxidant,[16] so that seemingly innocent weak interactions can have major implications on a catalytic reaction center.

**Figure 6:**
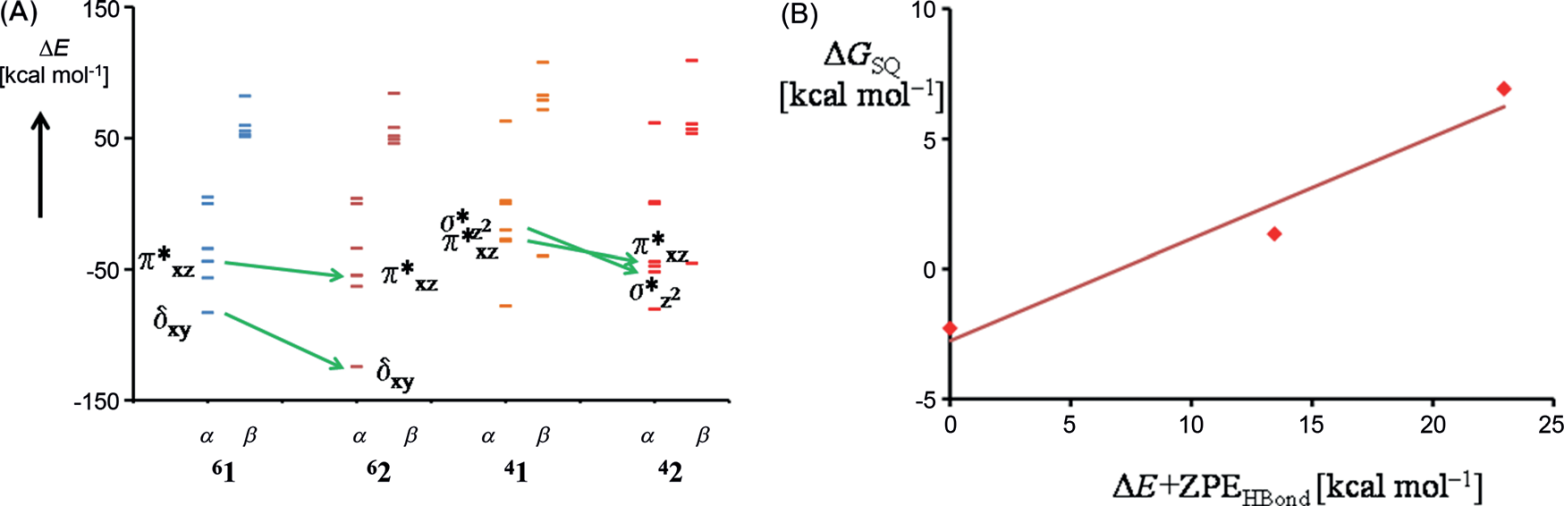
A) Orbital energy diagram of structures ^4,6^1 and ^4,6^2. B) Correlation between the sextet–quartet energy splitting (Δ*G*_SQ_) and the hydrogen-bond energy strength (Δ*E*+ZPE_HBond_), which is defined as the energy of the hydrogen-bonded complex with respect to three isolated species.

To further ascertain that hydrogen-bonding interactions affect the spin-state ordering and relative energies we calculated the strength of the hydrogen-bonding interaction from the difference in energy of ^6^**A** with the sum of ^4^**B**+2 PhOH and the sum of ^4^**B**+2 PhNH_2_. Figure 6 B indeed confirms that there is a linear correlation between the strength of the interaction of the phenol/aniline molecules with the iron(III) chloride complex and the corresponding quartet–sextet energy splitting.

Substrate binding to cytochrome P450s is a complex process and can trigger the change in the spin state from low spin to high spin in the heme iron and concomitantly induce a change in the reduction potential from about −300 to 100 mV more positive.[2, 3] The influence of a hydrogen bond at the coordinated imidazole on the geometric and electronic structure of an iron center has been investigated earlier with the model five-coordinate iron(II) porphyrinates.[6] Clear structural differences have been observed between iron(II) derivatives with either neutral imidazole or anionic imidazolate as the axial ligand, however, both types of species are found to be high-spin (*S*=2) systems only.[6] In fact, the hydrogen-bonding interaction with the coordinated imidazole increases both the Fe–N_p_ and Δ distances.[6] In sharp contrast, the hydrogen-bonding interactions between the phenol/2-isopropylaniline/aniline and axial chloride ligand in **2**/**3**/**4** result in an unusual lengthening of the Fe–Cl bonds, which eventually shorten both the Fe–N_p_ and Δ distances. This leads to stabilization of the intermediate-spin state (*S*=^3^/_2_) of the iron center in an iron(III) porphinato chloride, which is otherwise high spin (*S*=^5^/_2_) in nature. Furthermore, computational calculations clearly support the experimentally assigned spin state.

## Supporting Information

As a service to our authors and readers, this journal provides supporting information supplied by the authors. Such materials are peer reviewed and may be re-organized for online delivery, but are not copy-edited or typeset. Technical support issues arising from supporting information (other than missing files) should be addressed to the authors.

miscellaneous_information

## References

[1a] Jeffrey GA (1997). An introduction to hydrogen bonding.

[1b] Gordon MS, Jensen JH (1996). Acc. Chem. Res.

[2a] Ortiz de Montellano PR (2005). Cytochrome P450: Structure, Mechanism, and Biochemistry.

[2b] Denisov IG, Makris TM, Sligar SG, Schlichting I (2005). Chem. Rev.

[3a] Tripathi S, Li H, Poulos TL (2013). Science.

[3b] Mak PJ, Yang Y, Im S, Waskell LA, Kincaid JR (2012). Angew. Chem. Int. Ed.

[b01] (2012). Angew. Chem.

[3c] Nagano S, Poulos TL (2005). J. Biol. Chem.

[3d] Schlichting I, Berendzen J, Chu K, Stock AM, Maves SA, Benson DE, Sweet RM, Ringe D, Pestko GA, Sligar SG (2000). Science.

[4a] Shook RL, Borovik AS (2008). Chem. Commun.

[4b] Shook RL, Peterson SM, Greaves J, Moore C, Rheingold AL, Borovik AS (2011). J. Am. Chem. Soc.

[4c] Natale D, Mareque-Rivas JC (2008). Chem. Commun.

[4d] Sahu S, Widger LR, Quesne MG, de Visser SP, Matsumura H, Moënne-Loccoz P, Siegler MA, Goldberg DP (2013). J. Am. Chem. Soc.

[4e] Widger LR, Davies CG, Yang T, Siegler MA, Troeppner O, Jameson GNL, Ivanović-Burmazović I, Goldberg DP (2014). J. Am. Chem. Soc.

[5a] Blasiak LC, Vaillancourt FH, Walsh CT, Drennan CL (2006). Nature.

[5b] Marshall NM, Garner DK, Wilson TD, Gao Y-G, Robinson H, Nilges MJ, Lu Y (2009). Nature.

[5c] Umena Y, Kawakami K, Shen J-R, Kamiya N (2011). Nature.

[6a] Hu C, Sulok CD, Paulat F, Lehnert N, Twigg AI, Hendrich MP, Schulz CE, Scheidt WR (2010). J. Am. Chem. Soc.

[6b] Hu C, Noll BC, Piccoli PMB, Schultz AJ, Schulz CE, Scheidt WR (2008). J. Am. Chem. Soc.

[6c] Hu C, Noll BC, Schulz CE, Scheidt WR (2008). Inorg. Chem.

[7] http://www.ccdc.cam.ac.uk/data_request/cif.

[8a] Weiss R, Gold A, Terner J (2006). Chem. Rev.

[8b] Nakamura M (2006). Coord. Chem. Rev.

[9a] Sil D, Khan FST, Rath SP (2014). Inorg. Chem.

[9b] Ghosh SK, Bhowmik S, Sil D, Rath SP (2013). Chem. Eur. J.

[9c] Bhowmik S, Dey S, Sahoo D, Rath SP (2013). Chem. Eur. J.

[9d] Bhowmik S, Ghosh SK, Layek S, Verma HC, Rath SP (2012). Chem. Eur. J.

[9e] Bhowmik S, Ghosh SK, Rath SP (2011). Chem. Commun.

[9f] Ghosh SK, Rath SP (2010). J. Am. Chem. Soc.

[9g] Ghosh SK, Patra R, Rath SP (2010). Inorg. Chem.

[10a] Patra R, Chaudhury A, Ghosh SK, Rath SP (2008). Inorg. Chem.

[10b] Patra R, Rath SP (2009). Inorg. Chem. Commun.

[11a] Nakamura M, Ikeue T, Ohgo Y, Takahashi M, Takeda M (2002). Chem. Commun.

[11b] Cheng RJ, Chen PY, Gau PR, Chen CC, Peng SM (1997). J. Am. Chem. Soc.

[12a] Kumar D, Latifi R, Kumar S, Rybak-Akimova EV, Sainna MA, de Visser SP (2013). Inorg. Chem.

[12b] Vardhaman AK, Sastri CV, Kumar D, de Visser SP (2011). Chem. Commun.

[13] de Visser SP, Tan LS (2008). J. Am. Chem. Soc.

[14a] Sickerman NS, Park YJ, Ng GK-Y, Bates JE, Hilkert M, Ziller JW, Furche F, Borovik AS (2012). Dalton Trans.

[14b] Ni Z, Shores MP (2009). J. Am. Chem. Soc.

[14c] Ni Z, McDaniel AM, Shores MP (2010). Chem. Sci.

[14d] Young MC, Liew E, Hooley RJ (2014). Chem. Commun.

[15] de Visser SP, Ogliaro F, Gross Z, Shaik S (2001). Chem. Eur. J.

[16] Latifi R, Sainna MA, Rybak-Akimova EV, de Visser SP (2013). Chem. Eur. J.

