# Supplementary material for: Hydrogen-Bonding Interactions Trigger a Spin-Flip in Iron(III) Porphyrin Complexes
Source: Angew Chem Int Ed Engl. 2015 Jan 30;54(16):4796–800. doi: 10.1002/anie.201411399 (PMC4687417; doi:10.1002/anie.201411399)
Supplement: Supplementary file 1 — miscellaneous_information [file anie0054-4796-sd1.pdf]

Angewandte Chemie
Eine Zeitschrift der Gesellschaft Deutscher Chemiker

Supporting Information

Hydrogen-Bonding Interactions Trigger a Spin-Flip in Iron(III) Porphyrin Complexes**

Dipankar Sahoo, Matthew G. Quesne, Sam P. de Visser, and Sankar Prasad Rath**

anie_201411399_sm_miscellaneous_information.pdf

Supporting Information

Instrumentation.

UV-vis spectra were recorded on a PerkinElmer UV/vis spectrometer. Electron paramagnetic resonance (EPR) spectra were obtained on a Bruker EMX EPR spectrometer. Elemental (C, H, and N) analyses were performed on a Perkin-Elmer 2400II elemental analyzer. ^{1}H NMR spectra were recorded on a JEOL 500 MHz instrument. The spectra for paramagnetic molecules were recorded over a 100- kHz bandwidth with 64 K data points and a 5-ms 90° pulse. For a typical spectrum between 2000 and 3000 transients were accumulated with a 50-µs delay time. The residual ^{1}H resonances of the solvents were used as a secondary reference. ^{57}Fe Mössbauer spectra were recorded using a Wissel 1200 spectrometer and a proportional counter. $^{57}Co(Rh)$ in a constant acceleration mode was used as the radioactive source. Isomer shifts (δ) are given related to α-iron foil at room temperature. Magnetic susceptibility data were collected using a Quantum Design MPMS SQUID magnetometer over the temperature range 5 to 300K.

X-ray Structure Solution and Refinement.

Single-crystal X-ray data were collected at 100 K on a Bruker SMART APEX CCD diffractometer equipped with CRYO Industries low temperature apparatus and intensity data were collected using graphite-monochromated Mo Kα radiation (λ= 0.71073 Å). The data integration and reduction were processed with SAINT software.[1] An absorption correction was applied.[2] The structure was solved by the direct method using SHELXS-97 and was refined on F2 by full-matrix least-squares technique using the SHELXL-2014 program package.[3] Non-hydrogen atoms were refined anisotropically. In the refinement, hydrogens were treated as riding atoms using SHELXL default parameters. Crystal data and data collection parameters are given in Table S1. X-ray structure of **1** contains severely disordered solvent molecules (twelve cyclohexane and eight water molecules in the unit cell) which could not be modeled properly due to weakly diffracting nature of the crystal. SQUEEZE[4] routine of PLATON was therefore used to remove such unbound and highly disordered solvent molecules but the reported formula includes the solvents.

Computational Details.

Calculations were initiated from the crystal structure coordinates reported in this paper. Complex **1** represents [Fe^{III}(TDMOETPP)Cl], TDMOETPP=3,5-Dimethyl-2,3,7,8,12,13,17,18-octaethyl-5,10,l5,20-tetraphenylporphyrin. Hydrogen atoms were added to this complex to give the molecular formula $C_{68}H_{76}N_4FeCl$ and a total atom count of 150. The second structure **2** uses [Fe^{III}(TDMOETPP)Cl] in complex with two molecules of phenol, acting as the H-bond donors in the system. After adding the hydrogen atoms to crystal

structure **2**, we were therefore left with a system that has the molecular formula $C_{80}H_{88}N_4O_2FeCl$ and an atom count of 176. The crystal structure **4** has two aniline molecules in place of the phenol groups of structure **2** and therefore has the molecular formula $C_{86}H_{102}N_6FeCl$ with a total atom count of 178. All structures were optimized using the Jaguar 7.9 software package.[5] No constraints were placed on complex **1** with the H donor bonding distances being maintained for complexes **2** and **4**. Analytical frequencies were also carried out on all optimised structures using the Gaussian-09 software package.[6] Free energies were taken from the Gaussian frequencies and contain, zero-point, thermal and entropic corrections to the energy at 298.15 K and 1 atm.

Initial geometry optimisations were carried out on the doublet, quartet and sextet spin states for each structure using both the unrestricted hybrid density functional method B3LYP,[7] as well as the pure density functional method BP86.[8] A basis set containing the 6-31G+ double-ζ basis set on all atoms except for the iron were a double-ζ LACVP basis set, that includes an effective core potential, was used (basis set BS1).[9] Using the optimised structures for each spin state in complex **2** a geometry scan was run to increase the Cl---HOPh bond lengths from close to 2 Å to close to 3 Å, by moving the phenol molecules away in steps of 0.1 Å. All scan energies and geometries for the structure **2** complex were obtained using BS1 and energies were corrected using data from the vibrational frequencies. Additional gas phase, solvent corrected and dispersion corrected calculations were done with the triple-ζ LACV3P+ basis set on iron (with core potential) and 6-311+G* on the rest of the atoms (basis set BS2).

All three systems were also optimised using a slightly improved initial basis set (BS3) containing the polarised double-ζ basis 6-31G+* on all atoms except for the iron were the double-ζ basis with an effective core potential LACVP* was used. After crystal structure coordinate were optimized in BS3, using the same protocol as for BS1, a four basis set (BS4) were the triple-ζ LACV3P++** basis set on iron (with core potential) and 6-311++G** on the rest of the atoms was used. Single point gas phase, solvent corrected and dispersion corrected calculation were run applying the BS4 energies to BS3 optimisations.

The hydrogen bond strengths of the phenol and aniline bound complexes were determined from the reaction energies for equations 1 and 2 at UB3LYP/BS3 and include zero-point energies.

$^{6}\mathbf{A} \rightarrow {}^{4}\mathbf{B} + 2\ C_6H_5OH$ (1)

$^{6}\mathbf{A} \rightarrow {}^{4}\mathbf{C} + 2\ C_6H_5NH_2$ (2)

Experimental Section:

Materials:

Porphyrin free base 3,5-dimethyl-2,3,7,8,12,13,17,18-octaethyl-5,10,l5,20-tetraphenylporphyrin was prepared from 3,5-dimethyl benzaldehyde and the diethyl pyrrole using a literature procedure.[10] Reagents and solvents were purchased from commercial sources and purified by standard procedures before use.

Synthesis of 1:

3,5-Dimethyl-2,3,7,8,12,13,17,18-octaethyl-5,10,l5,20-tetraphenylporphyrin (100 mg, 0.105 mmol) was dissolved in 100 mL N, N-dimethylformamide. Excess $FeCl_2$ (66 mg, 0.51 mmol) was added and reflux for 2.5 hours under nitrogen. The resulting solution was then cooled to room temperature and transferred into a separetory funnel. 100 mL chloroform was added to the solution which was then washed well with 0.2(N) HCl (2×100 mL). Organic layer was separated and dried over anhydrous Na_2SO_4. The resulting solution was evaporated to dryness and purified by column chromatography using silica gel. The major fraction eluted with chloroform was collected and vacuum dried to obtain brown solid. Yield: 70 mg (64%). Anal. Calcd (found) for $C_{74}H_{90}N_4OFeCl$: C, 77.77 (78.00); H, 7.94 (8.22); N, 4.90 (5.11). UV-vis (benzene) [λ_{max}, nm (ε, M^{-1} cm^{-1})]: 443(2.4×10^4), 401(2.2×10^4), 578(2.0×10^1); ^{1}H NMR ($CDCl_3$, 295K): o-*H*,10.72,9.48; p-*H*, 8.21; H-*CH*$_3$*(m)*,3.81,3.41;-*CH*$_2$-, 45.43, 37.65, 34.47 and 23.52 ppm. EPR data: in solid (77 K), $g_\perp = 5.78$ and $g_{II} = 1.99$; in toluene (77 K), $g_\perp$=5.75 and g_{II}=1.99.

Synthesis of 2:

Compound **1** (100 mg, 0.096 mmol) was dissolved in chloroform (10 mL); phenol (45 mg, 0.48 mmol) was added and the mixture was stirred for 15 min under nitrogen atmosphere. The resulting solution was then carefully layered with n-hexane and kept for slow diffusion. On standing for 6–8 days in air at room temperature, dark-purple crystals were formed, which was collected by filtration, washed thoroughly with the mother liquor, and dried under vacuum. Yield: 71 mg (60%). Anal. Calcd (found) for $C_{94}H_{102}N_4O_4FeCl_7$: C, 68.18 (68.40); H, 6.21 (6.43); N, 3.38 (3.57). UV-vis (benzene) [λ_{max}, nm (ε, M^{-1} cm^{-1})]: 440 (3.2×10^4), 581 (2.2×10^1). ^{1}H NMR ($CDCl_3$, 295 K): o-*H*, 10.83, 10.29; p-*H*, 7.51; -*CH*$_2$-, 49.29, 24.93, 24.13 and 13.13 ppm. EPR data: in solid (77 K), $g_\perp$=4.16 and g_{II}=2.01; in toluene (77 K), $g_\perp = 4.18$ and $g_{II} = 2.00$.

Synthesis of 3:

Compound **1** (100 mg, 0.096 mmol) was dissolved in chloroform (10 mL); 2-isopropyl aniline (64 mg, 0.48 mmol) was added and the mixture was stirred for 15 min under nitrogen atmosphere. The resulting solution was then carefully layered with n-hexane and kept for slow diffusion in air at room temperature. On standing for 6–8 days, dark-purple crystals were formed, which was collected by filtration, washed thoroughly with the mother liquor, and dried under vacuum. Yield: 70 mg (55%). Anal. Calcd (found) for $C_{92}H_{109}N_7FeCl$: C, 78.69 (78.44); H, 7.82 (8.05); N, 6.98 (6.74). UV-vis (benzene) [λ_{max}, nm (ε, M^{-1} cm^{-1})]: 437(2.1×10^4). ^{1}H NMR ($CDCl_3$, 295 K): o-*H*, 11.01, 10.23; p-

H, 8.99; -CH_2-, 50.07, 25.07, 24.09 and 13.41 ppm. EPR data: in toluene (77 K), $g_{\perp}$ = 4.19 and g_{II} = 1.96.

References:

[1] SAINT+, 6.02 ed., Bruker AXS, Madison, WI, **1999**.

[2] G.M. Sheldrick, SADABS 2.0, **2000**.

[3] G. M. Sheldrick, *SHELXL-2014: Program for Crystal Structure Refinement*; University of Göttingen: Göttingen, Germany, **2014**.

[4] A. L. Spek, *J. Appl. Crystallogr.* **2003**, *36*, 7.

[5] *Jaguar,* version 7.9, Schrodinger, LLC, New York, NY, 2011.

[6] M. J. Frisch et al Gaussian 09, revision C.01; Gaussian, Inc., Wallingford, CT, 2010.

[7] (a) A. D. Becke, *J. Chem. Phys*. **1993**, *98*, 5648–5652. (b) C. Lee, W. Yang, R. G. Parr, *Phys. Rev. B* **1988**, *37*, 785–789.

[8] A. D. Becke, *Phys. Rev. A* **1988**, *38,* 3098–3100. (b) J. P. Perdew, *Phys. Rev. B* **1986**, *33*, 8822–8824.

[9] P. J. Hay, W. R.Wadt, W. R. *J. Chem. Phys*. **1985**, *82*, 270–283.

[10] K. M. Barkigia, M. Dolores Berber, J. Fajer, C. J. Medforth, M. W. Renner, K. M. Smith, *J. Am. Chem. Soc.* **1990**, *112*, 8851-8857.

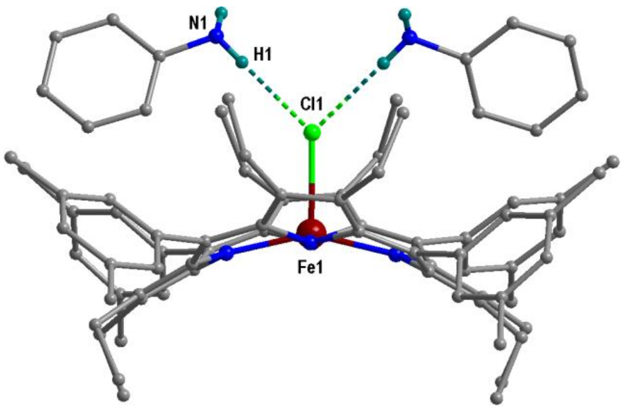

Figure S1. Molecular structures (at 100 K) of **4** (all the H atoms except for the aniline have been omitted for clarity). Selected bond distances (Å) and angle (deg) for **4**: Fe1–N1, 1.997(5); Fe1–N2, 1.976(4); Fe1–Cl1, 2.312(3); N1-Fe1-N2, 88.74(19); N1-Fe1-Cl1, 103.18(14); N2-Fe1-Cl1, 95.77(15). H-bonding interactions: N1L···Cl1, 3.318(7); N2L···N1L, 3.147(9).

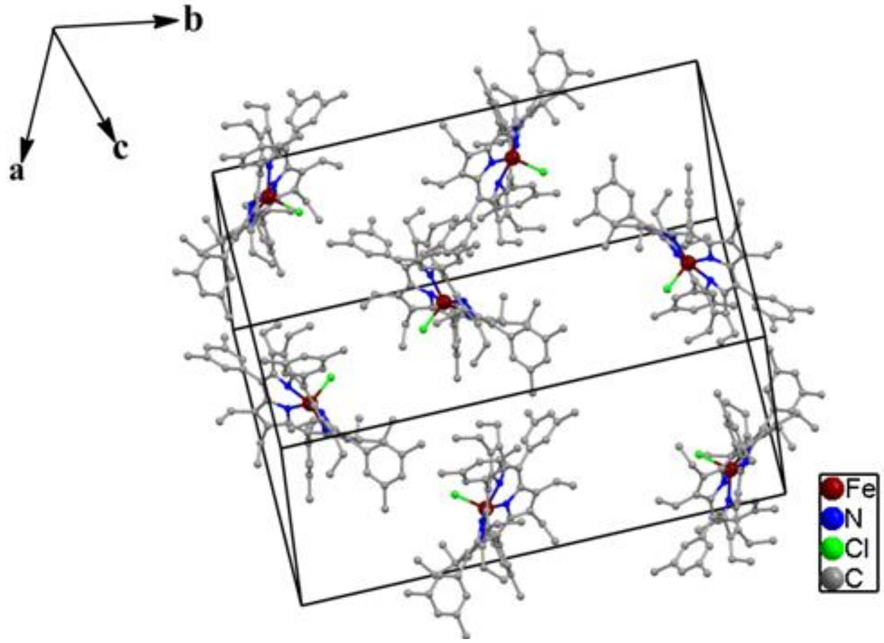

Figure S2. Diagram illustrating the packing of complex **1** in the unit cell (H atoms have been omitted for clarity).

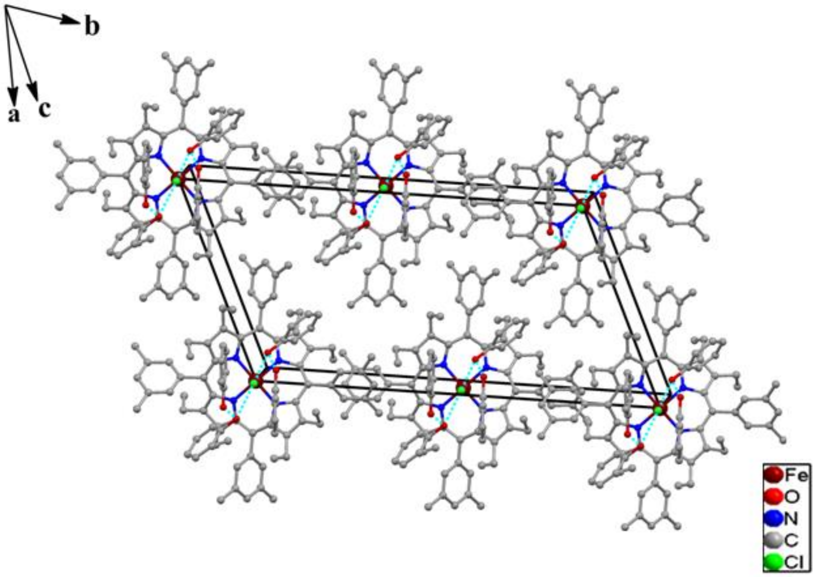

Figure S3. Diagram illustrating the packing of complex **2** in the unit cell (H atoms and $CHCl_3$ have been omitted for clarity).

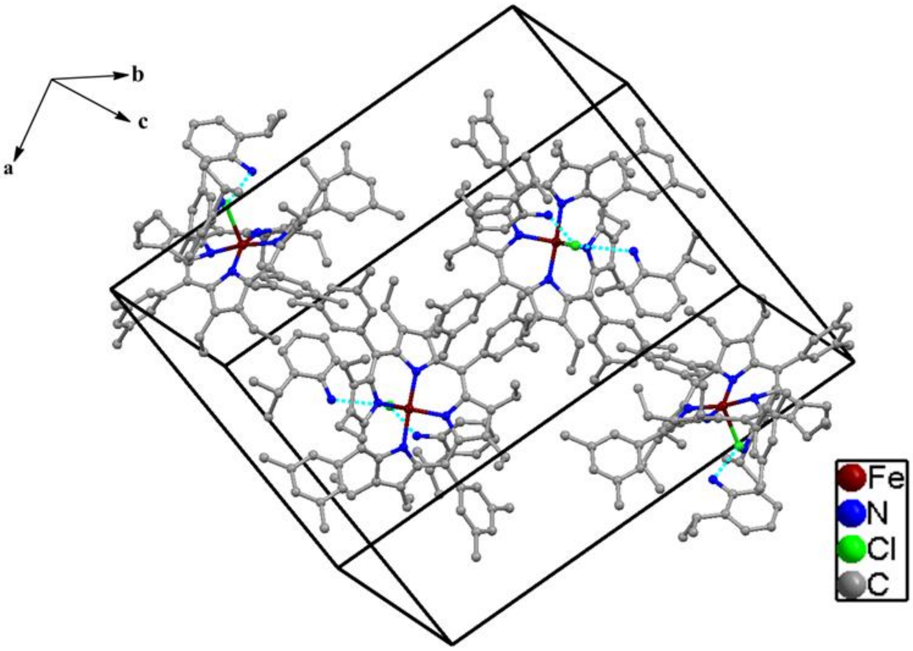

Figure S4. Diagram illustrating the packing of complex **3** in the unit cell (H atoms have been omitted for clarity)

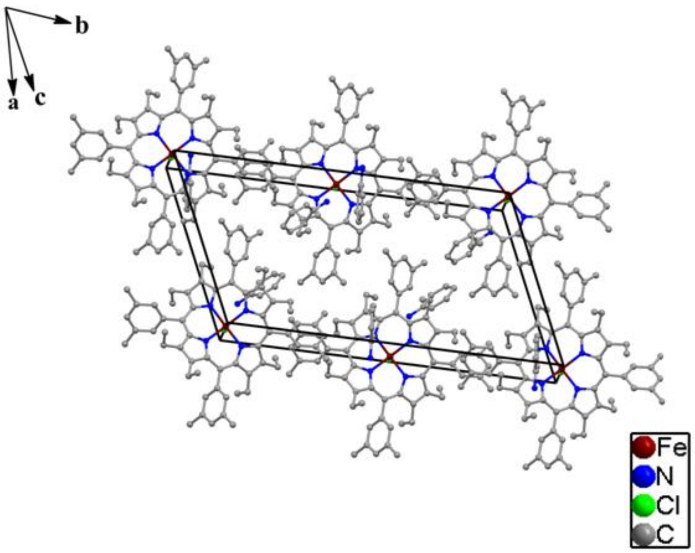

Figure S5. Diagram illustrating the packing of complex **4** in the unit cell (CH_2Cl_2 and H atoms have been omitted for clarity)

Table S1. Crystal data and data collection parameters.

	**1**	**2**	**3**	**4**
Formula	$C_{77} H_{96} Cl N_4 Fe O$	$C_{94} H_{102} Cl_7 N_4 O_4 Fe$	$C_{92} H_{109} Cl Fe N_7$	$C_{94} H_{108} Cl_5 Fe N_8$
T, (K)	100(2)	100(2)	100(2)	100(2)
Formula weight	1184.87	1655.79	1404.16	1582.98
Crystal system	Orthorombic	Monoclinic	Monoclinic	Monoclinic
Space group	*P*bca	*C* 2	*P 21/c*	*C* 2
a, Å	19.510(5)	26.129(5)	16.617(7)	26.066(5)
b, Å	24.761(5)	12.607(5)	19.957(9)	12.554(5)
c, Å	31.415(5)	13.960(5)	25.419(11)	13.936(5)
α, deg	90.000(5)	90.000(5)	90	90.000(5)
β, deg	90.000(5)	114.586(5)	108.342(9)	114.244(5)
γ, deg	90.000(5)	90.000(5)	90	90.000(5)
V, Å^3	15176(6)	4182(2)	8001(6)	4158(2)
Radiation (λ, Å)	Mo Kα (0.71073)	Mo Kα (0.71073)	Mo Kα (0.71073)	Mo Kα (0.71073)
Z	8	2	4	2
d_{calcd}, g.cm^{-3}	1.037	1.315	1.166	1.264
F(000)	5096	1742	3012	1678
μ, mm^{-1}	0.275	0.459	0.272	0.394
No. of unique data	13322	6310	15658	7496
No. of parameters, refined	684	511	895	507
GOF on F^2	0.899	1.048	0.982	1.036
$R1^a$ $[I > 2\sigma(I)]$	0.0734	0.0802	0.1114	0.0610
$R1^a$ (all data)	0.1259	0.0914	0.3061	0.0702
$wR2^b$ (all data)	0.2077	0.2332	0.3746	0.1732

a $R1 = \frac{\sum ||F_o| - |F_c||}{\sum |F_o|}$; b $wR2 = \sqrt{\frac{\sum [w(F_o^2 - F_c^2)^2]}{\sum [w(F_o^2)^2]}}$

Table S2. Selected Bond Distance (Ǻ) and Angles (°) for **1**, **2**, **3** and **4**.

	**1**	**2**	**3**	**4**
Fe(1)-N(1)	2.040(3)	1.997(6)	1.965(7)	1.997(5)
Fe(1)-N(2)	2.044(3)	1.960(5)	1.970(7)	1.976(4)
Fe(1)-N(3)	2.031(3)		1.937(7)	
Fe(1)-N(4)	2.046(3)		1.968(7)	
Fe(1)-Cl(1)	2.2287(12)	2.384(4)	2.357(3)	2.312(3)
N(1)-Fe(1)-N(2)	87.05(12)	89.5 (2)	89.2(3)	88.74(19)
N(1)-Fe(1)-N(4)	87.17(12)		88.5(3)	
N(2)-Fe(1)-N(4)	145.46(12)		155.7(3)	
N(3)-Fe(1)-N(1)	159.09(12)		169.9(3)	
N(3)-Fe(1)-N(2)	86.61(12)		87.9(3)	
N(3)-Fe(1)-N(4)	86.82(11)		90.2(3)	
N(1)-Fe(1)-Cl(1)	100.26(9)	102.3(2)	95.6(2)	103.18(14)
N(2)-Fe(1)-Cl(1)	107.27(9)	94.6(2)	100.8(2)	95.77(15)
N(3)-Fe(1)-Cl(1)	100.66(9)		94.4(2)	
N(4)-Fe(1)-Cl(1)	107.27(9)		103.5(2)	

Table S3. Selected structural parameters for [Fe^{III}(TDMOETPP)Cl], **1** and related species

Complex	Fe-N_p^{a}	Fe-Cl	$\Delta^{Fe}{}_{4N}$^{b}	$\Delta^{Fe}{}_{24}$^{c}	Δ_{24}^{d}	Ref
**1**	2.040(3)	2.2287(12)	0.49	0.52	0.52	tw
**2**	1.978(6)	2.384(4)	0.29	0.27	0.56	tw
**3**	1.960(7)	2.357(3)	0.29	0.30	0.56	tw
**4**	1.986(5)	2.312(3)	0.32	0.31	0.56	tw
[Fe^{III}(OMTPP)Cl]	2.034(6)	2.24 (3)	0.46	0.51	0.51	9
[Fe^{III}(OETPP)Cl]	2.031(5)	2.2418(23)	0.47	0.43	0.56	9
[Fe^{III}(detpp)Cl]	2.075(4)	2.220(2)	0.491	0.54	0.14	10
[Fe^{III}(trans-tetpp)Cl]	2.064(3)	2.219(1)	0.494	0.54	0.35	10
[Fe^{III}(cis-tetpp)Cl]	2.065(1)	2.226(1)	0.477	0.52	0.34	10
[Fe^{III}(hetpp)Cl]	2.053(2)	2.239(1)	0.490	0.52	0.45	10
[Fe^{III}(DPP)Cl]	2.056(5)	2.238(2)	0.514	0.57	0.47	11
[Fe^{III}(*tn*-OEP)Cl]	2.067(3)	2.1918(10)	0.46	0.47	0.41	12
[Fe^{III}(OEP)Cl]	2.065(3)	2.243(13)	0.468	0.518	0.047	13
[Fe^{III}(TEtP)(Cl)]	2.053(3)	2.2644(13)	0.47	0.45	0.027	14

^{a}Average value in Å. ^{b}Displacement of iron from mean plane containing four porphyrinic nitrogen. ^{c}Displacement of iron from mean plane containing $C_{20}N_4$ porphyrinic core. ^{d}Average displacement of the 24 atoms from the least-squares plane of the porphyrin.

[9] R. J. Cheng, P.Y. Chen, P. R. Gau, C. C. Chen, S. M. Peng, *J. Am. Chem. Soc.* **1997**, *119*, 2563-2569.

[10] R. Weiss, J. Fischer, V. Bulach, J. A. Shelnutt, *C. R. Chimie*. **2002**, *5*, 405-416.

[11] R. Weiss, J. Fischer, V. Bulach, V. Schünemann, M. Gerdan, A. X. Trautwein, J. A. Shelnutt, C. P. Gros, A.Tabard, R. Guilard, *Inorg. Chim. Acta.* **2002**, *337*, 223-232.

[12] R. Patra, A. Chaudhary, S. K. Ghosh S. P. Rath, *Inorg. Chem*. **2008**, *47*, 8324-8335.

[13] M. K. Safo, K. E. Buentello, A. G. Oliver , W. R. Scheidt, *Acta Cryst. E.* **2010**, *66*, m733.

[14] M. Li, T. J. Neal, N. Ehlinger, C. E. Schulz, W. R. Scheidt, *J.Porphyrins Phthalocyanines* **2010**, *14*, 115.

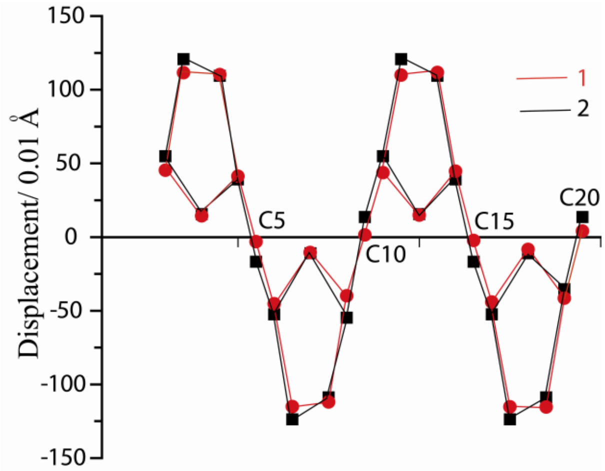

Figure S6. Atom deviations (in units of 0.01 Å) from the least-squares plane of the $C_{20}N_4$ porphyrinato core of **1** and **2**. The horizontal axis represents the atom number in the macrocycle showing the bond connectivity between atoms.

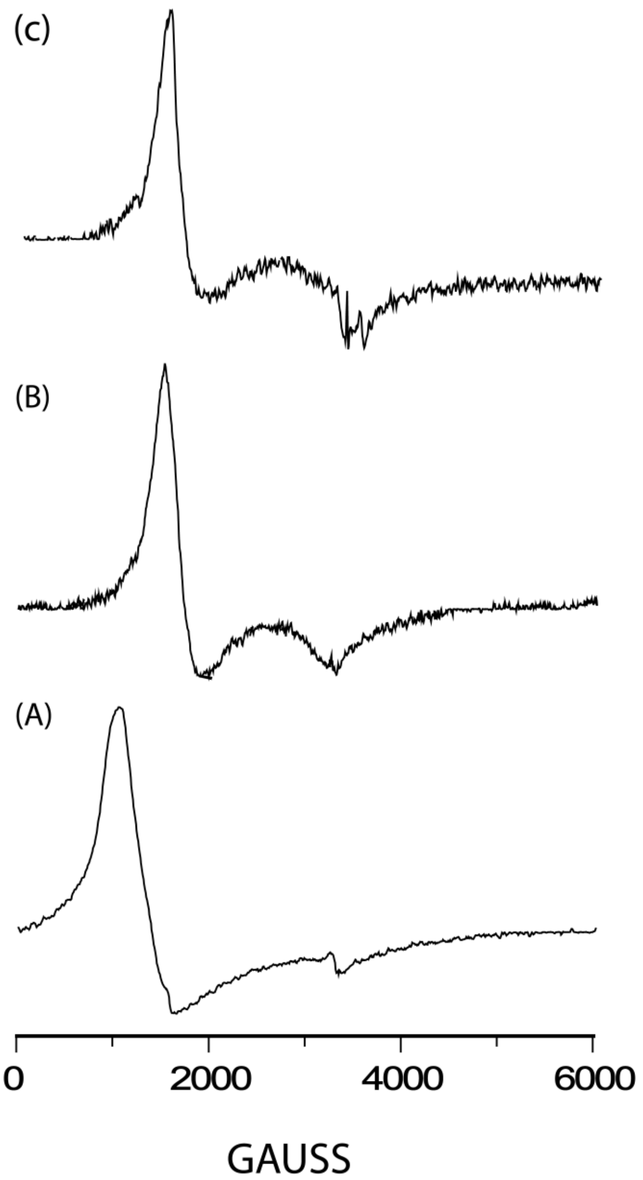

Figure S7. EPR spectra of (A) **1**, (B) **2** and (C) **3** in solid at 77 K.

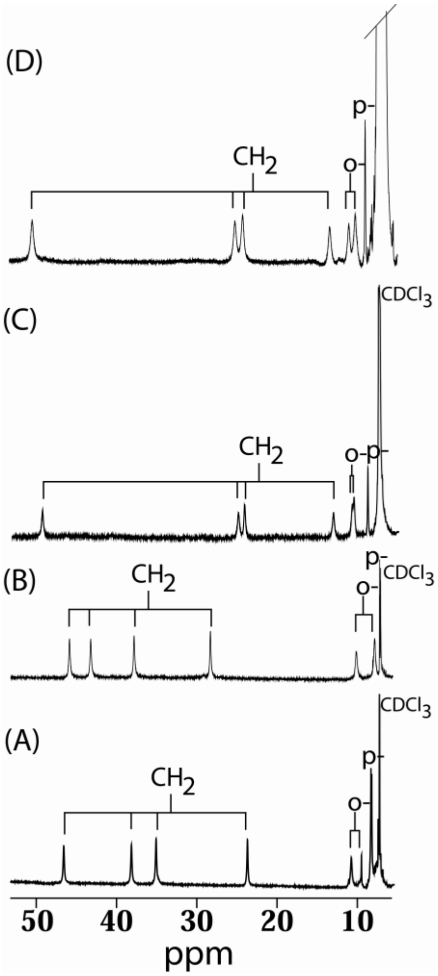

Figure S8. ^{1}H NMR (at 295 K in $CDCl_3$) spectra of (A) **1** and (B) five-coordinate phenolato iron(III)octaethyltetraarylporphyrin, (C) **2** and (D) **3**.

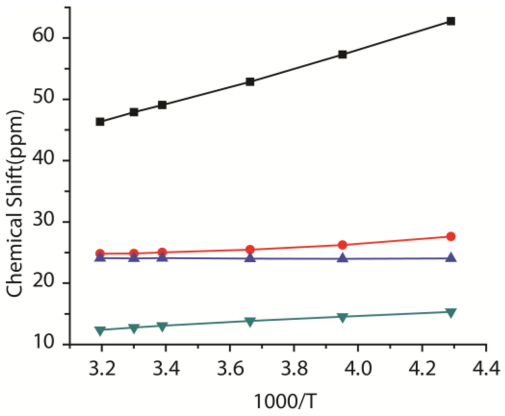

Figure S9. Curie plots (chemical shift versus 1000/T) of the methylene proton signals for complex **2**.

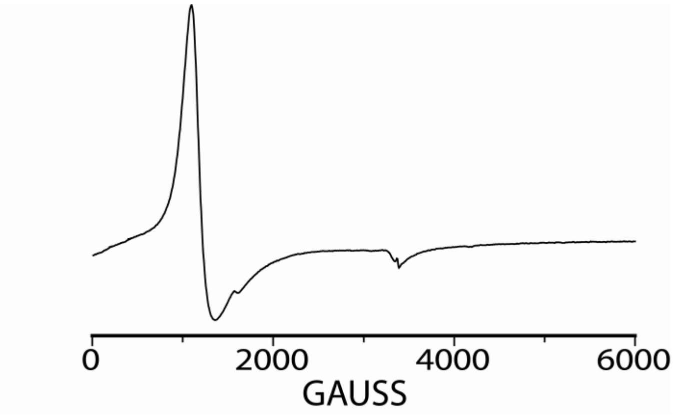

Figure S10. EPR spectrum of five-coordinate phenolato iron(III)octaethyltetraarylporphyrin in solid at 77 K. Simulation of the spectra gives the following values: $g_{\perp}$= 5.91 and g_{II} =1.97.

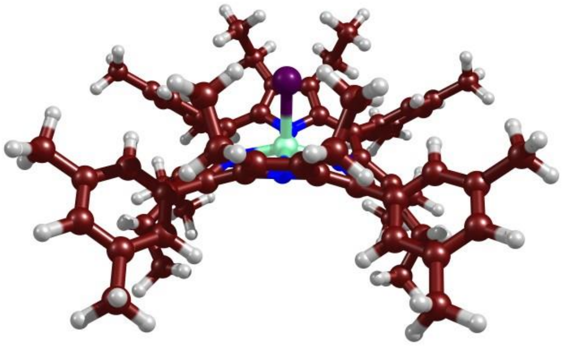

Fe,Cl = 2.344 {2.405} [2.347]
Fe,N_{ave} = 1.965 {1.975} [2.066]
Δ^{Fe} = 0.266 {0.306} [0.273]

^{2}A {^{4}A} [^{6}A]

Figure S11. Bonding data of the complex **1** with no H bond donor calculated at B3LYP/BS1.

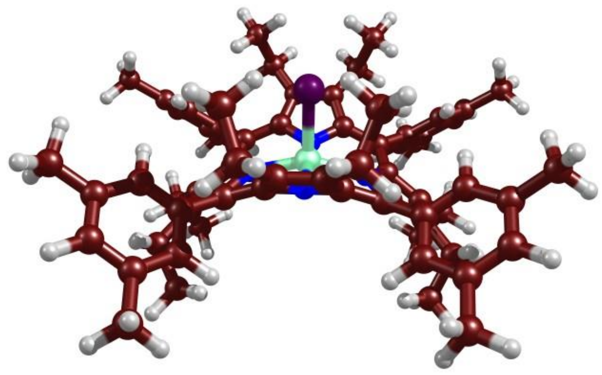

Fe,Cl = 2.290 {2.351} [2.294]

Fe,N_{ave} = 1.975 {1.994} [2.081]

Δ^{Fe} = 0.273 {0.326} [0.513]

^{2}A {^{4}A} [^{6}A]

Figure S12. Bonding data of the complex **1** with no H bond donor calculated at UB3LYP/BS3.

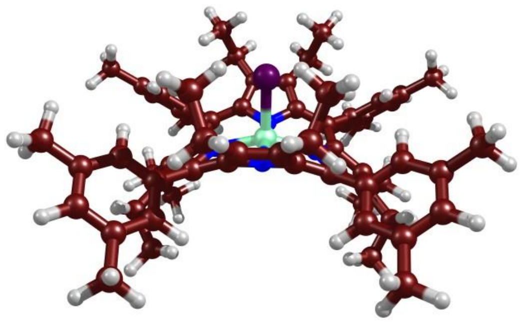

Fe,Cl = 2.320 {2.399} [2.329]

Fe,N_{ave} = 1.951 {1.967} [2.081]

Δ^{Fe} = 0.240 {0.284} [0.500]

^{2}A {^{4}A} [^{6}A]

Figure S13. Bonding data of the complex **1** with no H bond donor calculated at BP86/BS1.

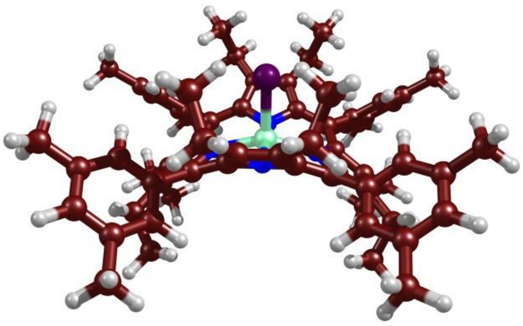

Fe,Cl = 2.262 {2.371} [2.292]
Fe,N_{ave} = 1.966 {1.973} [2.086]
Δ^{Fe} = 0.243 {0.277} [0.496]

^{2}A {^{4}A} [^{6}A]

Figure S14. Bonding data of the complex **1**with no H bond donor calculated at BP86/BS1.

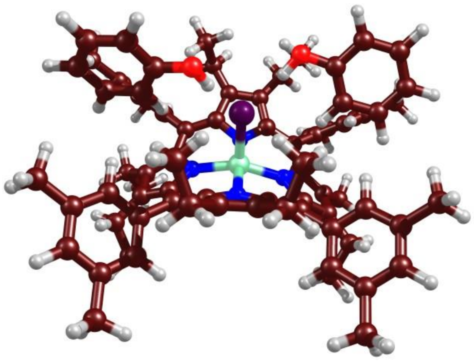

Fe,Cl = 2.450 {2.511} [2.438]
Fe,N_{ave} = 1.963 {1.963} [2.054]
Δ^{Fe} = 0.268 {0.259} [0.426]

^{2}B {^{4}B} [^{6}B]

Figure S15. Bonding data of the complex **2** with two phenol H Donor molecules calculated at UB3LYP/BS1.

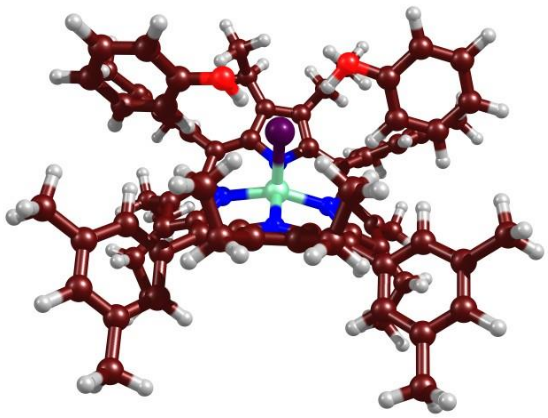

Fe,Cl = 2.554 {2.485} [2.392]
Fe,N_{ave} = 1.986 {1.975} [2.070]
Δ^{Fe} = 0.193 {0.253} [0.452]

^{2}B {^{4}B} [^{6}B]

Figure S16. Bonding data of the complex **2** with two phenol H Donors added as calculated at UB3LYP/BS3.

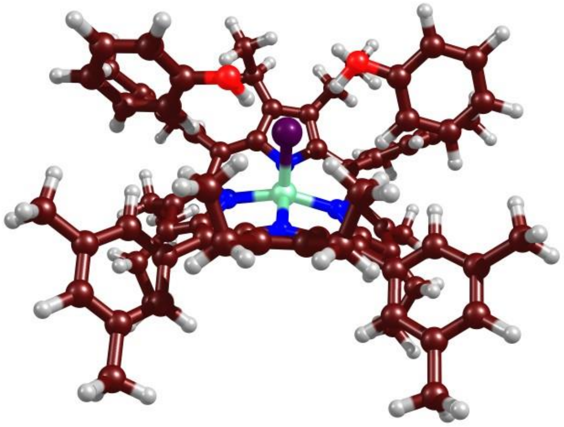

Fe,Cl = 2.420 {2.523} [2.440]
Fe,N_{ave} = 1.945 {1.946} [2.057]
Δ^{Fe} = 0.247 {0.239} [0.411]

^{2}B {^{4}B} [^{6}B]

Figure S17. Bonding data of the complex **2** with two hydrogen bonded phenol molecules as calculated at BP86/BS1.

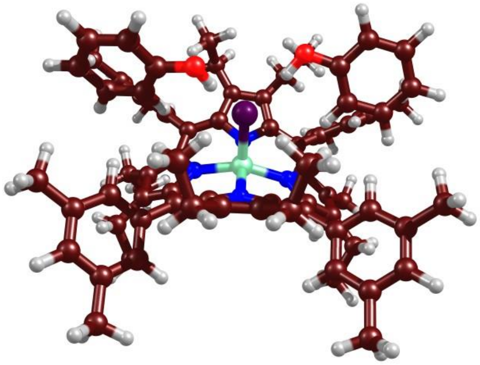

Fe,Cl = 2.375 {2.471} [2.398]
Fe,N_{ave} = 1.962 {1.969} [2.069]
Δ^{Fe} = 0.249 {0.233} [0.448]

^{2}B {^{4}B} [^{6}B]

Figure S18. Bonding data of the complex **2** with two hydrogen bonded phenol molecules added, as calculated at UBP86/BS3.

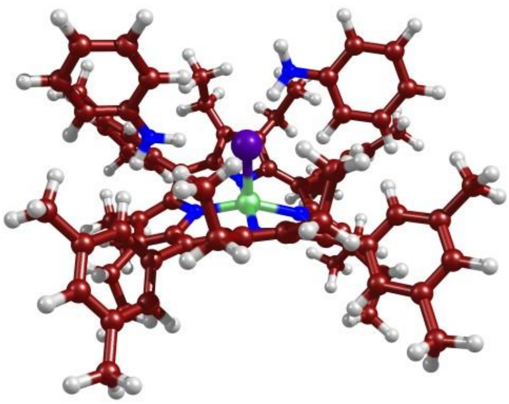

Fe,Cl = 2.400 {2.472} [2.482]
Fe,N_{ave} = 1.966 {1.967} [1.975]
Δ^{Fe} = 0.251 {0.271} [0.250]

^{2}A {^{4}A} [^{6}A]

Figure S19. Bonding data of the complex **4** with two hydrogen bonded aniline molecules added, as calculated at UB3LYP/BS1.

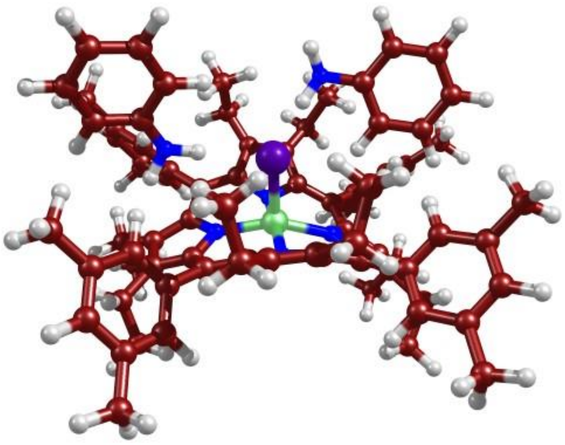

Fe,Cl = 2.360 {2.440} [2.359]
Fe,N_{ave} = 1.978 {1.983} [2.071]
Δ^{Fe} = 0.257 {0.273} [0.441]

^{2}A {^{4}A} [^{6}A]

Figure S20. Bonding data of the complex **4** with two hydrogen bonded aniline molecules added, as calculated at UB3LYP/BS3.

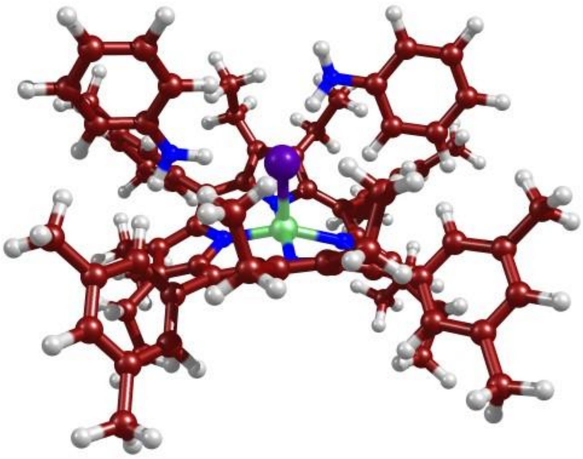

Fe,Cl = 2.372 {2.474} [2.508]
Fe,N_{ave} = 1.953 {1.956} [1.962]
Δ^{Fe} = 0.240 {0.242} [0.229]

^{2}A {^{4}A} [^{6}A]

Figure S21. Bonding data of the complex **4** with two hydrogen bonded aniline molecules added, as calculated at UBP86/BS1.

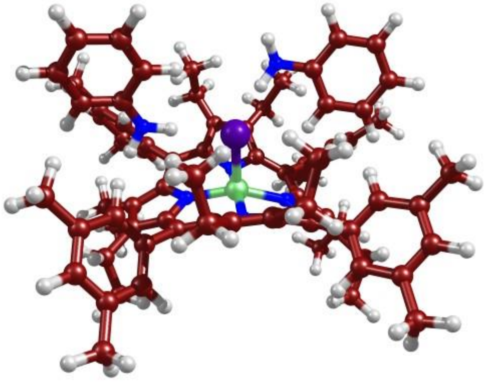

Fe,Cl = 2.327 {2.448} [2.469]
Fe,N_{ave} = 1.967 {1.970} [1.969]
Δ^{Fe} = 0.237 {0.237} [0.219]

^{2}A {^{4}A} [^{6}A]

Figure S22. Bonding data of the complex **4** with two hydrogen bonded aniline molecules added, as calculated at UBP86/BS3.

Table S4: Optimized geometries of ^{2,4,6}1, ^{2,4,6}**2** and ^{2,4,6}**4** using different computational methods and basis sets.

**$BS1_{OPT}$**							
**B3LYP**	Fe,Cl	Fe,N	Δ_{Fe}	**BP86**	Fe,Cl	Fe,Nave	ΔFe
^{2}**1**	2.344	1.965	0.266	^{2}**1**	2.320	1.951	0.240
^{4}**1**	2.405	1.975	0.306	^{4}**1**	2.400	1.967	0.284
^{6}**1**	2.347	2.066	0.273	^{6}**1**	2.329	2.081	0.500
^{2}**2**	2.450	1.963	0.268	^{2}**2**	2.420	1.950	0.247
^{4}**2**	2.511	1.963	0.259	^{4}**2**	2.523	1.953	0.239
^{6}**2**	2.438	2.054	0.426	^{6}**2**	2.440	2.057	0.411
^{2}**4**	2.400	1.966	0.251	^{2}**4**	2.372	1.953	0.240
^{4}**4**	2.472	1.967	0.271	^{4}**4**	2.474	1.956	0.242
^{6}**4**	2.482	1.975	0.250	^{6}**4**	2.508	1.962	0.229
**$BS3_{OPT}$**							
**B3LYP**	Fe,Cl	Fe,Nave	ΔFe	**BP86**	Fe,Cl	Fe,Nave	ΔFe
^{2}**1**	2.289	1.975	0.273	^{2}**1**	2.262	1.966	0.243
^{4}**1**	2.351	1.994	0.326	^{4}**1**	2.371	1.973	0.277
^{6}**1**	2.294	2.081	0.513	^{6}**1**	2.292	2.086	0.496
^{2}**2**	2.554	1.986	0.193	^{2}**2**	2.375	1.962	0.249
^{4}**2**	2.485	1.975	0.253	^{4}**2**	2.471	1.969	0.233
^{6}**2**	2.392	2.070	0.452	^{6}**2**	2.398	2.069	0.448
^{2}**4**	2.360	1.978	0.257	^{2}**4**	2.327	1.967	0.237
^{4}**4**	2.440	1.983	0.273	^{4}**4**	2.448	1.970	0.237
^{6}**4**	2.359	2.071	0.441	^{6}**4**	2.469	1.969	0.219

Table S5. Absolute energies B3LYP/BS1 optimised

	E BS1	Zero-point BS1	Gibbs BS1	E BS2	E_{solv} (Kcal/mol) BS2	E_{D3} BS2
^{2}**1**	-3439.123	1.282	-3437.965	-3440.458	-16.110	-0.221
^{4}**1**	-3439.141	1.282	-3437.978	-3440.477	-18.015	-0.221
^{6}**1**	-3439.130	1.281	-3437.974	-3440.475	-16.727	-0.220
^{2}**2**	-4053.930	1.495	-4052.569	-4055.560	-20.126	-0.273
^{4}**2**	-4053.952	1.495	-4052.594	-4055.584	-19.979	-0.271
^{6}**2**	-4053.935	1.494	-4052.576	-4055.575	-20.364	-0.270
^{2}**4**	-4014.215	1.521	-4012.829	-4015.808	-18.666	-0.264
^{4}**4**	-4014.235	1.521	-4012.856	-4015.829	-19.771	-0.264
^{6}**4**	-4014.176	1.517	-4012.797	-4015.771	-18.264	-0.262

Table S6. Absolute energies B3LYP/BS3 optimised

	E BS3	zero BS3	Gibbs BS3	E BS4	E_{solv} BS4	E_{D3} BS4
^{2}**1**	-3439.770	1.275	-3438.613	-3440.567	-15.887	-0.223
^{4}**1**	-3439.787	1.275	-3438.629	-3440.585	-17.602	-0.223
^{6}**1**	-3439.778	1.273	-3438.626	-3440.583	-15.034	-0.222
^{2}**2**	-4054.716	1.485	-4053.369	-4055.695	-14.182	-0.275
^{4}**2**	-4054.741	1.487	-4053.389	-4055.726	-14.080	-0.273
^{6}**2**	-4054.720	1.486	-4053.371	-4055.715	-18.244	-0.270
^{2}**4**	-4014.979	1.513	-4013.604	-4015.948	-15.120	-0.281
^{4}**4**	-4014.999	1.513	-4013.623	-4015.970	-15.486	-0.280
^{6}**4**	-4014.985	1.511	-4013.618	-4015.964	-16.036	-0.275

Table S7. Relative energies B3LYP/BS1 optimised

	ΔE [BS1]	ΔE_{ZPE} [BS1]	ΔE [BS2]	ΔE_{ZPE} [BS2]	ΔG [BS1]	ΔG [BS2]	ΔE_{S+ZPE} [BS2]	ΔG_{S} [BS2]	ΔE_{D3+ZPE} [BS2]	ΔG_{D3} [BS2]
^{2}**1**	0.00	0.00	0.00	0.00	0.00	0.00	0.00	0.00	0.00	0.00
^{4}**1**	-11.29	-11.36	-12.01	-12.08	-8.26	-8.98	-13.99	-10.88	-11.78	-8.68
^{6}**1**	-4.43	-5.37	-10.35	-11.29	-5.72	-11.63	-11.90	-12.25	-10.60	-10.94
^{2}**2**	0.00	0.00	0.00	0.00	0.00	0.00	0.00	0.00	0.00	0.00
^{4}**2**	-14.04	-14.04	-14.62	-14.62	-15.29	-15.87	-14.48	-15.72	-13.72	-14.97
^{6}**2**	-3.24	-4.20	-9.11	-10.08	-4.14	-10.01	-10.32	-10.25	-8.42	-8.36
^{2}**4**	0.00	0.00	0.00	0.00	0.00	0.00	0.00	0.00	0.00	0.00
^{4}**4**	-12.89	-12.57	-13.21	-12.89	-16.85	-17.17	-14.00	-18.27	-12.51	-16.78
^{6}**4**	24.15	22.08	22.95	20.87	19.79	18.58	21.27	18.99	22.12	19.84

Table S8. Relative energies B3LYP/BS3 optimised

	ΔE [BS3]	ΔE_{ZPE} [BS3]	ΔE [BS4]	ΔE_{ZPE} [BS4]	ΔG [BS4]	ΔG [BS4]	ΔE_{S+ZPE} [BS4]	ΔG_{S} [BS4]	ΔE_{D3+ZPE} [BS4]	ΔG_{D3} [BS4]
^{2}**1**	0.00	0.00	0.00	0.00	0.00	0.00	0.00	0.00	0.00	0.00
^{4}**1**	-10.68	-10.69	-11.74	-11.76	-9.83	-10.89	-13.47	-12.61	-11.54	-10.67
^{6}**1**	-5.26	-6.18	-10.49	-11.40	-8.05	-13.27	-10.55	-12.42	-11.08	-12.95
^{2}**2**	0.00	0.00	0.00	0.00	0.00	0.00	0.00	0.00	0.00	0.00
^{4}**2**	-15.19	-14.40	-19.53	-18.74	-12.31	-16.64	-18.64	-16.54	-17.37	-15.27
^{6}**2**	-2.25	-2.00	-12.51	-12.26	-1.40	-11.66	-16.32	-15.72	-8.94	-8.34
^{2}**4**	0.00	0.00	0.00	0.00	0.00	0.00	0.00	0.00	0.00	0.00
^{4}**4**	-12.71	-12.81	-14.13	-14.23	-11.51	-12.93	-14.59	-13.29	-14.12	-12.82
^{6}**4**	-3.89	-5.07	-10.35	-11.52	-8.51	-14.96	-12.44	-15.88	-8.04	-11.48

Table S9. Group spin densities and charges obtained from the UB3LYP/BS2/Gas-phase calculations.

**Spin**						**charge**				
	Fe	Cl	lig	HD1	HD1	Fe	Cl	lig	HD1	HD1
^{2}**1**	1.22	-0.07	-0.14			0.18	-0.31	0.13		
^{4}**1**	2.75	0.25	-0.01			0.20	-0.36	0.16		
^{6}**1**	4.12	0.27	0.61			0.22	-0.27	0.05		
^{2}**2**	1.31	-0.07	-0.23	-0.01	0.00	0.43	-0.46	0.19	-0.08	-0.08
^{4}**2**	2.82	0.15	0.02	0.01	0.01	0.44	-0.49	0.22	-0.09	-0.09
^{6}**2**	4.13	0.16	0.69	0.01	0.01	0.51	-0.37	0.04	-0.08	-0.09
^{2}**4**	1.29	-0.08	-0.21	0.00	-0.01	0.35	-0.40	0.52	-0.43	-0.04
^{4}**4**	2.80	0.17	0.01	0.01	0.01	0.37	-0.44	0.58	-0.45	-0.05

Table S10. Group spin densities and charges obtained from the UBP86/BS2/Gas-phase calculations.

Spin						charge				
**BP86**	Fe	Cl	lig	HD1	HD1	Fe	Cl	lig	HD1	HD1
^{2}**1**	1.04	0.00	-0.04			-0.02	-0.26	0.28		
^{4}**1**	2.58	0.31	0.10			0.01	-0.31	0.29		
^{6}**1**	4.00	0.32	0.68			0.02	-0.23	0.21		
^{2}**2**	1.04	-0.03	-0.01	-0.01	0.00	0.23	-0.42	0.35	-0.08	-0.08
^{4}**2**	2.62	0.16	0.20	0.01	0.01	0.25	-0.49	0.40	-0.08	-0.08
^{6}**2**	4.02	0.17	0.79	0.01	0.01	0.35	-0.37	0.18	-0.07	-0.08
^{2}**4**	1.04	-0.02	-0.02	0.01	-0.01	0.13	-0.35	0.72	-0.44	-0.06
^{4}**4**	2.61	0.20	0.18	0.01	0.01	0.19	-0.39	0.78	-0.51	-0.07

Table S11. Group spin densities and charges obtained from the UB3LYP/BS2/Solvent calculations.

Spin						Charge				
	Fe	Cl	lig	HD1	HD1	Fe	Cl	lig	HD1	HD1
^{2}**1**	1.19	-0.06	-0.13			0.27	-0.48	0.21		
^{4}**1**	2.81	0.19	0.00			0.32	-0.54	0.22		
^{6}**1**	4.13	0.23	0.64			0.33	-0.42	0.10		
^{2}**2**	1.25	-0.06	-0.18	-0.01	0.00	0.46	-0.48	0.21	-0.10	-0.09
^{4}**2**	2.85	0.13	0.01	0.01	0.01	0.50	-0.53	0.23	-0.10	-0.10
^{6}**2**	4.14	0.15	0.69	0.01	0.01	0.55	-0.39	0.05	-0.10	-0.11
^{2}**4**	1.23	-0.07	-0.16	0.00	-0.01	0.40	-0.41	0.52	-0.46	-0.04
^{4}**4**	2.82	0.16	0.00	0.01	0.01	0.41	-0.46	0.59	-0.48	-0.06

Table S12. Group spin densities and charges obtained from the UB3LYP/BS2/Solvent calculations.

Spin						Charge				
	Fe	Cl	lig	HD1	HD1	Fe	Cl	lig	HD1	HD1
^{2}**1**	1.04	-0.01	-0.03			0.06	-0.42	0.36		
^{4}**1**	2.63	0.24	0.14			0.11	-0.51	0.39		
^{6}**1**	4.00	0.26	0.73			0.11	-0.38	0.27		
^{2}**2**	1.04	-0.03	-0.01	-0.01	0.00	0.28	-0.46	0.38	-0.10	-0.10
^{4}**2**	2.64	0.14	0.20	0.01	0.01	0.30	-0.52	0.43	-0.10	-0.10
^{6}**2**	4.02	0.16	0.80	0.01	0.01	0.39	-0.40	0.20	-0.09	-0.10
^{2}**4**	1.04	-0.02	-0.02	0.01	-0.01	0.15	-0.36	0.75	-0.48	-0.06
^{4}**4**	2.62	0.18	0.18	0.01	0.01	0.22	-0.41	0.81	-0.53	-0.09

Table S13. Group spin densities and charges obtained from the UB3LYP/BS4/Gas-phase calculations.

Spin						**charge**				
	Fe	Cl	Lig	HD1	HD2		Fe	Cl	lig	HD1	HD2
^{2}**1**	1.21	-0.05	-0.16				0.24	-0.34	0.11		
^{4}**1**	2.78	0.26	-0.05				0.23	-0.39	0.15		
^{6}**1**	4.10	0.29	0.61				0.25	-0.29	0.04		
^{2}**2**	1.95	-0.02	-0.93	0.00	0.00		0.35	-0.46	0.23	-0.06	-0.05
^{4}**2**	2.83	0.14	0.02	0.00	0.00		0.35	-0.42	0.19	-0.06	-0.07
^{6}**2**	4.12	0.17	0.70	0.01	0.01		0.51	-0.31	-0.06	-0.07	-0.07
^{2}**4**	1.32	-0.07	-0.25	0.00	0.00		0.33	-0.34	0.34	-0.27	-0.05
^{4}**4**	2.83	0.18	-0.02	0.01	0.00		0.35	-0.41	0.38	-0.28	-0.04
^{6}**4**	4.12	0.20	0.67	0.01	0.00		0.46	-0.29	0.15	-0.27	-0.04

Table S14. Group spin densities and charges obtained from UB3LYP/BS4/Gas-phase calculations.

Spin Multiplicity						Charge				
	Fe	Cl	lig	HD1	HD2		Fe	Cl	lig	HD1	HD2
^{2}**1**	1.04	0.02	-0.06				0.06	-0.31	0.25		
^{4}**1**	2.59	0.33	0.08				0.07	-0.33	0.25		
^{6}**1**	3.99	0.34	0.67				0.09	-0.26	0.17		
^{2}**2**	1.07	-0.03	-0.04	0.00	0.00		0.16	-0.36	0.23	-0.02	0.00
^{4}**2**	2.64	0.18	0.17	0.01	0.01		0.26	-0.44	0.27	-0.04	-0.05
^{6}**2**	4.00	0.18	0.80	0.01	0.01		0.30	-0.34	0.14	-0.06	-0.04
^{2}**4**	1.06	-0.02	-0.04	0.00	0.00		0.16	-0.28	0.49	-0.30	-0.07
^{4}**4**	2.63	0.21	0.14	0.01	0.01		0.17	-0.34	0.51	-0.30	-0.04

Table S15. Group spin densities and charges obtained from UB3LYP/BS4/Solvent calculations.

Spin						Charge				
	Fe	Cl	lig	HD1	HD2	Fe	Cl	lig	HD1	HD2
^{2}**1**	1.19	-0.04	-0.15			0.33	-0.50	0.17		
^{4}**1**	2.83	0.21	-0.04			0.36	-0.56	0.21		
^{6}**1**	4.11	0.24	0.65			0.35	-0.42	0.07		
^{2}**2**	1.94	-0.02	-0.92	0.00	0.00	0.41	-0.48	0.20	-0.07	-0.06
^{4}**2**	2.85	0.14	0.00	0.00	0.00	0.41	-0.43	0.14	-0.06	-0.07
^{6}**2**	4.13	0.16	0.70	0.01	0.01	0.55	-0.33	-0.05	-0.08	-0.09
^{2}**4**	1.27	-0.06	-0.20	0.00	0.00	0.37	-0.36	0.37	-0.32	-0.06
^{4}**4**	2.84	0.18	-0.03	0.01	0.00	0.40	-0.43	0.40	-0.33	-0.04
^{6}**4**	4.14	0.20	0.65	0.01	0.00	0.50	-0.30	0.17	-0.32	-0.05

Table S16. Group spin densities and charges obtained from UBP86/BS4/Solvent calculations.

Spin						Charge				
	Fe	Cl	lig	HD1	HD2	Fe	Cl	lig	HD1	HD2
^{2}**1**	1.05	0.01	-0.06			0.15	-0.46	0.31		
^{4}**1**	2.62	0.26	0.12			0.17	-0.51	0.34		
^{6}**1**	3.99	0.29	0.73			0.18	-0.40	0.22		
^{2}**2**	1.24	-0.03	-0.21	0.00	0.00	0.20	-0.37	0.22	-0.04	-0.02
^{4}**2**	2.66	0.15	0.17	0.01	0.01	0.32	-0.47	0.29	-0.06	-0.07
^{6}**2**	4.01	0.17	0.81	0.01	0.01	0.34	-0.36	0.14	-0.07	-0.05
^{2}**4**	1.05	-0.02	-0.03	0.00	0.00	0.18	-0.29	0.52	-0.35	-0.07
^{4}**4**	2.64	0.20	0.14	0.01	0.01	0.23	-0.36	0.53	-0.35	-0.05

Table S17. Bonding data for complex 2 throughout scan, whereby the Cl–HO distance is fixed.

^{2}**B3LYP**	Fe,Cl	Fe,H_{Sub}	Fe,N_{ave}	Δ_{Fe}	^{2}**BP86**	Fe,Cl	Fe,H_{Sub}	Fe,N_{ave}	Δ_{Fe}
scan01	2.436	2.284	1.964	0.261	scan01	2.41	2.28	1.95	0.25
scan02	2.435	2.384	1.964	0.261	scan02	2.41	2.38	1.95	0.25
scan03	2.428	2.484	1.966	0.259	scan03	2.40	2.48	1.95	0.25
scan04	2.419	2.584	1.965	0.258	scan04	2.39	2.58	1.95	0.25
scan05	2.417	2.684	1.964	0.260	scan05	2.38	2.68	1.95	0.25
scan06	2.410	2.784	1.964	0.259	scan06	2.38	2.78	1.95	0.25
scan07	2.405	2.884	1.966	0.258	scan07	2.38	2.88	1.95	0.25
scan08	2.399	2.984	1.966	0.257	scan08	2.38	2.98	1.95	0.25
^{4}**B3LYP**	Fe,Cl	Fe,H_{Sub}	Fe,N_{ave}	Δ_{Fe}	^{4}**BP86**	Fe,Cl	Fe,H_{Sub}	Fe,N_{ave}	Δ_{Fe}
scan01	2.50	2.28	1.96	0.27	scan01	2.51	2.28	1.95	0.24
scan02	2.49	2.38	1.96	0.27	scan02	2.50	2.38	1.95	0.25
scan03	2.49	2.48	1.97	0.27	scan03	2.50	2.48	1.96	0.25
scan04	2.48	2.58	1.97	0.27	scan04	2.49	2.58	1.96	0.25
scan05	2.48	2.68	1.97	0.28	scan05	2.48	2.68	1.96	0.25
scan06	2.47	2.78	1.97	0.28	scan06	2.48	2.78	1.96	0.25
scan07	2.47	2.88	1.97	0.28	scan07	2.47	2.88	1.96	0.26
scan08	2.46	2.98	1.97	0.28	scan08	2.47	2.98	1.96	0.26
^{6}**B3LYP**	Fe,Cl	Fe,H_{Sub}	Fe,N_{ave}	Δ_{Fe}	^{6}**BP86**	Fe,Cl	Fe,H_{Sub}	Fe,N_{ave}	Δ_{Fe}
scan01	2.430	2.284	2.055	0.429	scan01	2.430	2.281	2.058	0.417
scan02	2.424	2.384	2.056	0.432	scan02	2.424	2.381	2.061	0.428
scan03	2.419	2.484	2.057	0.442	scan03	2.418	2.481	2.063	0.428
scan04	2.413	2.584	2.057	0.437	scan04	2.413	2.581	2.065	0.427
scan05	2.409	2.684	2.058	0.444	scan05	2.407	2.681	2.064	0.432
scan06	2.404	2.784	2.058	0.443	scan06	2.405	2.781	2.067	0.432
scan07	2.401	2.884	2.060	0.444	scan07	2.400	2.881	2.068	0.433
scan08	2.397	2.984	2.061	0.443	scan08	2.395	2.981	2.068	0.429

Table S18. Orbital energies for 4,61, 4,62 and 4,64 (in au) as obtained from UB3LYP/BS3.

	^{6}**1**		^{6}**2**		^{6}**4**		^{4}**1**		^{4}**2**		^{4}**4**	
	alpha	beta	alpha	beta	alpha	beta	alpha	beta	alpha	beta	alpha	beta
δ_{xy}	-0.3103	-0.0938	-0.3865	-0.1062	-0.3866	-0.1035	-0.3027	-0.2421	-0.3120	-0.2561	-0.3127	-0.2568
σ^*_{z2}	-0.2680	-0.0895	-0.2888	-0.1152	-0.2819	-0.1066	-0.2106	-0.0642	-0.2668	-0.0871	-0.2637	-0.0848
π^*_{yz}	-0.2479	-0.0827	-0.2751	-0.0957	-0.2680	-0.0934	-0.2242	-0.0525	-0.2544	-0.0933	-0.2475	-0.0941
π^*_{xz}	-0.2476	-0.0964	-0.2756	-0.1101	-0.2700	-0.1084	-0.2221	-0.0466	-0.2597	-0.0985	-0.2502	-0.0980
σ^*_{x2-y2}	-0.2328	-0.0469	-0.2430	-0.0541	-0.2393	-0.0518	-0.0782	-0.0071	-0.0858	-0.0103	-0.0886	-0.0123
a_{1u}	-0.1780		-0.1885		-0.1860		-0.1786		-0.1841		-0.1883	
a_{2u}	-0.1701		-0.1823		-0.1779		-0.1753		-0.1820		-0.1840	

Cartesians

^{2}1{B3LYP}$_{\text{optBS1}}$

6	8.164658670	30.097718357	31.142833509
6	7.654562539	30.090199747	29.721589063
1	7.678400360	29.063998485	29.337251640
1	6.610499014	30.402721463	29.677215166
6	8.485335331	30.989701685	28.775988320
1	8.090447827	30.941894440	27.753841965
1	8.455878425	32.034445708	29.103456815
1	9.534767238	30.674867278	28.752801638
6	9.528534975	27.924918356	30.967220119
1	8.889036183	27.715402634	30.102202595
6	10.981006097	28.118762370	30.470988731
1	11.324702817	27.229050229	29.929486525
1	11.056388080	28.980164533	29.797717529
6	5.934582353	32.205940404	31.234472306
6	5.814344737	33.113385424	30.168163730
1	6.598191491	33.847921012	30.007385541
6	4.703468447	33.081043411	29.310219470
6	3.703528163	32.122429709	29.543189493
1	2.840493672	32.085457742	28.882273316
6	3.791585317	31.207754029	30.606533568
6	4.919494842	31.255182221	31.440480475
6	4.575625857	34.074640375	28.174180307
1	5.554908940	34.334628017	27.757865400
1	3.956771894	33.677002178	27.362703716
1	4.108209840	35.009162743	28.514122447
6	2.682160754	30.208045996	30.858289578
1	1.895822862	30.639875375	31.492710254
1	2.207911897	29.892029153	29.922414315
1	3.056823570	29.314372622	31.368166342
6	10.082004782	24.579236661	33.840305664
1	10.124455041	23.494527535	33.775689158
6	12.617096071	24.609339916	33.718885090
1	13.015912165	24.377117020	34.715788383
1	12.532842163	23.663070659	33.173642673
6	7.550640212	24.404281754	33.959306320
1	7.741905352	23.351834192	34.191695682
1	7.048989027	24.441007225	32.982812198
26	9.599218443	32.233787814	34.456865208
7	8.560915660	33.632118135	33.544148120
7	8.885877976	30.903466345	33.198377708
7	10.742072136	30.853343589	35.244633587
7	10.885795560	33.611857565	35.030452925
17	8.122280202	32.233052199	36.276715770
6	8.527690409	34.955268745	33.978141083
6	7.164247069	35.462629247	33.868274895
6	6.410022316	34.468042640	33.262665346
6	7.315387015	33.357700687	32.981331141
6	7.125993044	32.245643432	32.141552128
6	8.035996907	31.168443148	32.125414054
6	8.991705309	29.132351628	31.698179552
6	9.362080937	29.600690610	33.029543664
6	9.915425492	28.866492264	34.097289952
6	10.400014846	29.502804361	35.255775000
6	10.761168112	28.929021548	36.546877371
6	11.419752451	29.919700055	37.262067221
6	11.482514558	31.096783706	36.402056050
6	12.270962618	32.252468726	36.529403825
6	12.086324846	33.375144844	35.693139389
6	12.972587470	34.518741957	35.494054973
6	12.241109894	35.487093394	34.823028760
6	10.898435827	34.948115142	34.628531588
6	9.711654698	35.638240128	34.310922998
6	6.605203093	36.740679629	34.446899273
1	5.660369372	36.978842695	33.944033501
1	7.268306101	37.585082053	34.253973940
6	6.345733323	36.646474794	35.969637041
1	5.933702183	37.590454381	36.347452415
1	5.637598208	35.843635967	36.200359403
1	7.272798882	36.434500527	36.512136264
6	4.908284912	34.492366828	33.106910853
1	4.556587329	35.527157178	33.190014883
1	4.605191318	34.149413007	32.116058867
6	4.181719696	33.637808457	34.173593229
1	3.094719900	33.705938321	34.041865715
1	4.471148845	32.585037393	34.096823971
1	4.429789327	33.974919283	35.185342356
1	9.488158386	27.033270551	31.594075147
1	11.661973862	28.289472789	31.311688264
6	10.364790700	27.581638189	37.101476963
1	10.513204846	26.793469958	36.362268401
1	11.022368558	27.333108842	37.942774536
6	8.897786811	27.538238564	37.591657359
1	8.657634311	26.546628865	37.994739352
1	8.721565690	28.281508341	38.376276735
1	8.204248639	27.752190568	36.772013291
6	11.814782485	29.819981176	38.715965807
1	11.871217090	28.763032292	39.000999374
1	12.810504890	30.232047500	38.887011348
6	10.812232957	30.532073117	39.656411320
1	11.124484429	30.420849918	40.702144133
1	10.751344415	31.600659803	39.427632491
1	9.805693611	30.114579257	39.549216541
6	14.451610452	34.596641709	35.789476401
1	14.763610299	35.647478048	35.781107348
1	14.673663892	34.225751863	36.791318921
6	15.313674653	33.818353239	34.767476505
1	16.379106130	33.914126043	35.009860336
1	15.058610620	32.753144732	34.771057672
1	15.158189124	34.197115773	33.750877442
6	12.806822188	36.766191679	34.253670549
1	12.154293461	37.615200045	34.462107943
1	13.759683817	36.988243131	34.747578236
6	13.048354095	36.688161322	32.727756362
1	13.481579759	37.625441610	32.358064593
1	13.735133158	35.871317069	32.478409538
1	12.109446222	36.512886777	32.191631592
1	5.015372950	30.548887338	32.259802831
6	9.973167755	27.372469455	34.005092163
6	8.792814766	26.611911150	34.025726554
1	7.837064633	27.121645457	34.104735385
6	8.832386143	25.210223619	33.948809051
6	11.279998974	25.314135747	33.812878098
6	11.210353649	26.712457329	33.900646807
1	12.123829842	27.299907003	33.888695396
1	13.360703997	25.230057422	33.207884799
6	13.337542358	32.306855570	37.579122551
6	13.210250326	33.184270609	38.667445313
1	12.330546958	33.817507508	38.735919770
6	14.197858394	33.250312939	39.664929773
6	15.325013675	32.422680702	39.548240073
1	16.096249453	32.466168788	40.314046774
6	15.481652321	31.536602781	38.466920104
6	14.477208750	31.486844506	37.490249503
1	14.573652631	30.804281056	36.651044504
6	16.698021977	30.639189739	38.380441083
1	16.623598624	29.799992818	39.085170655
1	16.812172163	30.217345417	37.377005228
1	17.616842557	31.185170156	38.624326556
6	14.055024604	34.213959671	40.824274371
1	13.013492332	34.286365416	41.156301351
1	14.662709478	33.903010514	41.680373097
1	14.376829230	35.225962734	40.542695906
6	9.708044390	37.134663007	34.350883290
6	9.538608485	37.878633027	33.168745257
1	9.404989387	37.354020923	32.227671031
6	9.529644589	39.280112796	33.189109759
6	9.683787228	39.934769627	34.424616357
6	9.851330583	39.219918543	35.620830391
6	9.864827455	37.815583886	35.568695626
1	9.991958000	37.242823319	36.482170723
1	6.842518586	24.791202342	34.700762632
6	10.035100258	39.942268232	36.939168127
1	11.099854507	40.060633276	37.183313753
1	9.592556928	40.943626108	36.911573233
1	9.572962934	39.389800459	37.764465086
6	9.353919072	40.077515515	31.913599619
1	8.526479421	40.792823663	32.000367733
1	10.256461935	40.655413990	31.675715317
1	9.143808121	39.424248523	31.061089689
1	9.667065487	41.022029868	34.454820633

^{2}1{B3LYP}$_{\text{optBS3}}$

6	8.182141498	30.062120308	31.176253435
6	7.701205577	30.032484056	29.745178174
1	7.711453850	28.996718728	29.388439375
1	6.665433527	30.363684057	29.669149990
6	8.570415286	30.887406924	28.801921164
1	8.195147045	30.829030136	27.772512804
1	8.562320316	31.939695743	29.105516897
1	9.612722597	30.547388953	28.804478635
6	9.553086113	27.895688446	31.054701497
1	8.948968315	27.687679126	30.165080399
6	11.019046220	28.085856659	30.618081606
1	11.383305519	27.194586998	30.092181052
1	11.123975727	28.945201615	29.945891345
6	6.004519870	32.213821352	31.168599211
6	5.960516918	33.118847964	30.099317220
1	6.771575492	33.833652736	29.978041700
6	4.898578042	33.112827996	29.188562556
6	3.868561104	32.182016019	29.372175510
1	3.038717812	32.163569927	28.666670522
6	3.877658255	31.271708563	30.436962854
6	4.959182922	31.293442515	31.324340408
6	4.854612810	34.104482498	28.046696364
1	5.858332072	34.320221573	27.663734523
1	4.247214349	33.731565221	27.214669124
1	4.418160268	35.060502124	28.367098011
6	2.735857037	30.298901817	30.632895508
1	1.965319739	30.721535622	31.291610393
1	2.251401463	30.051534876	29.681462429
1	3.077873587	29.365250291	31.093167195
6	9.953579847	24.601739866	33.977781491
1	9.970361427	23.513429760	33.930816625
6	12.482804204	24.565832022	33.834109845
1	12.890732535	24.355744983	34.832034914
1	12.369251049	23.604481886	33.320789956
6	7.423743108	24.488788983	34.124402485
1	7.589426477	23.435228997	34.373670984
1	6.913779381	24.522362512	33.152154586
26	9.571652211	32.240244653	34.486822693
7	8.545044194	33.632399868	33.536651499
7	8.859529362	30.889484176	33.231943136
7	10.744360747	30.867289770	35.264738742
7	10.843635015	33.641292109	35.086934364
17	8.122119804	32.231006289	36.258992271
6	8.478949325	34.939210669	33.976872480
6	7.120491795	35.438615787	33.835289110
6	6.396005745	34.439738675	33.216117745
6	7.322004420	33.347428265	32.962140503
6	7.152786562	32.230652814	32.124021191
6	8.046174563	31.141132795	32.145080399
6	8.991334372	29.107648057	31.758012228
6	9.333284480	29.600203563	33.082996862
6	9.884665402	28.883188460	34.163651669
6	10.387599826	29.532606852	35.305371145
6	10.767441025	28.963493315	36.587309486
6	11.464495556	29.944220517	37.266219486
6	11.496838552	31.111285858	36.397712903
6	12.273083792	32.278108916	36.519683728
6	12.044913303	33.422296547	35.727728562
6	12.906180661	34.587100186	35.555176629
6	12.154680695	35.542489546	34.903599154
6	10.832861031	34.966054499	34.701704758
6	9.641599122	35.632330874	34.353575314
6	6.533757622	36.715324649	34.387795651
1	5.595189917	36.930294210	33.864355588
1	7.185547322	37.566469066	34.190785661
6	6.251053088	36.640474322	35.901574841
1	5.821715112	37.585289904	36.257904279
1	5.547455711	35.834014191	36.135291323
1	7.170146850	36.448938769	36.465190443
6	4.897958516	34.440174902	33.030520850
1	4.530844776	35.469774100	33.102866548
1	4.618016015	34.090765968	32.035522898
6	4.169962155	33.583536190	34.085963589
1	3.084141967	33.633837003	33.936250237
1	4.475534925	32.534125188	34.022578225
1	4.394056073	33.929847249	35.100564125
1	9.482360527	27.005132768	31.680329919
1	11.669219165	28.258550104	31.482488767
6	10.343685285	27.637934784	37.173022531
1	10.466275626	26.827999164	36.453056612
1	11.000308627	27.393067130	38.015167125
6	8.884495814	27.649010675	37.671907458
1	8.620078675	26.676303008	38.106640773
1	8.731436008	28.420204613	38.435531230
1	8.189593350	27.856774183	36.851584855
6	11.932499186	29.836082399	38.697656564
1	12.000061355	28.776868174	38.969625669
1	12.938086138	30.240779805	38.818084788
6	10.985864444	30.540666067	39.690599859
1	11.357256536	30.432044241	40.717125024
1	10.904368294	31.609536122	39.468394350
1	9.976624515	30.117792192	39.643929323
6	14.381971069	34.708774723	35.852406354
1	14.646109676	35.770547815	35.907392690
1	14.624874982	34.290705283	36.829012713
6	15.267546370	34.034972819	34.785249273
1	16.329497465	34.152177301	35.034454724
1	15.053597316	32.963138488	34.715746202
1	15.099782827	34.473552777	33.794809735
6	12.693747999	36.840921429	34.350502855
1	12.028581171	37.677588227	34.568277907
1	13.641411102	37.072347180	34.848305234
6	12.942140050	36.782342622	32.830162601
1	13.364415348	37.729395919	32.471834809
1	13.641536367	35.978697175	32.572842752
1	12.010537295	36.600158089	32.284315821
1	4.996898483	30.583929553	32.148099620
6	9.912921190	27.390485242	34.097419300
6	8.719010266	26.659650405	34.140668507
1	7.774395699	27.193361531	34.218743973
6	8.723772522	25.260857985	34.088156479
6	11.165228152	25.303915636	33.926688267
6	11.129361483	26.700292286	33.993524286
1	12.057605779	27.266379959	33.966805296
1	13.233765440	25.152363764	33.293177812
6	13.401159169	32.284696295	37.500874981
6	13.344397096	33.058386694	38.664594426
1	12.465202735	33.670904875	38.852200227
6	14.396902696	33.047663495	39.590515555
6	15.517342952	32.256654104	39.319606411
1	16.342306263	32.244120500	40.031010491
6	15.607581420	31.478202429	38.156505791
6	14.537273692	31.497247488	37.260065624
1	14.576499510	30.890718892	36.358007024
6	16.825849816	30.620277870	37.898171519
1	16.833571768	29.735540054	38.548000013
1	16.854572795	30.268457741	36.861817999
1	17.753652553	31.171012632	38.093808876
6	14.324619034	33.892457210	40.842535539
1	13.332494407	33.837989627	41.304833939
1	15.062171308	33.572130020	41.585662296
1	14.518372955	34.949548370	40.619233945
6	9.616618768	37.123814904	34.397014286
6	9.491246780	37.861692659	33.209966399
1	9.399962205	37.332350596	32.264903569
6	9.476014607	39.258125236	33.226586758
6	9.570772339	39.912256238	34.463489238

6	9.691337114	39.204842798	35.663650819
6	9.717908530	37.804120326	35.615423283
1	9.815558207	37.232165041	36.535312916
1	6.731616014	24.906926597	34.865035890
6	9.814813774	39.929520117	36.985651975
1	10.856302171	39.945248136	37.333907483
1	9.480055018	40.969047937	36.905718959
1	9.221456072	39.441301917	37.767190724
6	9.364925881	40.052988284	31.944833242
1	8.596704068	40.831893820	32.020745107
1	10.310116337	40.556736549	31.704950977
1	9.109767026	39.409072802	31.097671841
1	9.547672770	41.001331070	34.489652652

^{4}1{B3LYP}$_{optBS1}$

6	8.220042154	30.111693903	31.102430380
6	7.728306386	30.101490974	29.675219402
1	7.773289400	29.077426193	29.287502730
1	6.679695439	30.398117873	29.619852842
6	8.556064075	31.018196237	28.744228900
1	8.177225966	30.966139429	27.716274876
1	8.502946167	32.061254075	29.073962198
1	9.611278895	30.722273302	28.736201969
6	9.544168313	27.909302312	30.974743458
1	8.904523641	27.692070542	30.111669978
6	11.001055919	28.073616978	30.481064462
1	11.335452975	27.168510897	29.959604744
1	11.090495551	28.920218927	29.790705829
6	5.971324922	32.187717850	31.197017845
6	5.792948026	33.070290176	30.120293910
1	6.544789426	33.829885610	29.926370306
6	4.659883586	32.983741102	29.292975192
6	3.702570219	31.994355732	29.566022815
1	2.823612512	31.917716998	28.929666198
6	3.851951479	31.098951766	30.640566210
6	4.994954255	31.204707703	31.444566217
6	4.465377563	33.956641318	28.148594435
1	5.421656848	34.233236753	27.691336956
1	3.825961087	33.532444163	27.367237637
1	3.989187513	34.884999782	28.493065998
6	2.797922181	30.047134345	30.917678806
1	1.834931169	30.505907572	31.177109038
1	2.626369720	29.411116861	30.039803043
1	3.091460607	29.398958446	31.748987456
6	9.964317592	24.591355620	33.934539280
1	9.978446668	23.504601667	33.896437767
6	12.500377681	24.550567626	33.843618992
1	12.879497127	24.329876142	34.850770598
1	12.397088121	23.594888133	33.318350799
6	7.427621411	24.486887385	34.031795429
1	7.590519454	23.431462865	34.271509206
1	6.925009305	24.530667522	33.056321646
26	9.591834792	32.259882854	34.466128710
7	8.596122656	33.651869381	33.508191706
7	8.912195320	30.919135268	33.164320972
7	10.757528269	30.879008881	35.225509679
7	10.902539693	33.639476974	35.044718912
17	8.016130509	32.158591360	36.280842149
6	8.549573731	34.970121314	33.965154344
6	7.182386993	35.463730224	33.849841577
6	6.441416662	34.472919482	33.219087748
6	7.355245170	33.375110353	32.927021745
6	7.177134342	32.267650968	32.081543376
6	8.092326686	31.191588099	32.073244389
6	9.022410819	29.137299273	31.681028521
6	9.379464472	29.613803187	33.012657185
6	9.907042188	28.885672753	34.098936135
6	10.388309629	29.533012316	35.251343129
6	10.727358035	28.973619781	36.553857233
6	11.404783038	29.960340756	37.258702552
6	11.498702912	31.121789943	36.384269812
6	12.306839278	32.265121670	36.506077212
6	12.118214271	33.391001342	35.677334092
6	12.998171431	34.536794262	35.480014346
6	12.251942934	35.516512851	34.839141122
6	10.910561372	34.975373269	34.648006706
6	9.719840861	35.660931353	34.328058852
6	6.603900169	36.725618637	34.444470507
1	5.663718931	36.964193904	33.933446251
1	7.261797035	37.579055236	34.275916382
6	6.325205141	36.601246279	35.961768242
1	5.900730682	37.535307908	36.350200707
1	5.621079489	35.788906380	36.169172796
1	7.246491829	36.386849662	36.512619455
6	4.940720041	34.491834174	33.051578407
1	4.585033310	35.525933563	33.126393787
1	4.646949217	34.142633524	32.060528597
6	4.208969493	33.639963210	34.117225911
1	3.123062095	33.703770222	33.975572596
1	4.502966133	32.588179388	34.048467062
1	4.447468317	33.982271698	35.129286308
1	9.488763188	27.031115220	31.619328599
1	11.680259168	28.252899901	31.321451437
6	10.290748498	27.648173143	37.130103318
1	10.422160034	26.842468797	36.406939047
1	10.935059467	27.397864783	37.981066533
6	8.818981911	27.656373722	37.609091831
1	8.547871573	26.679278500	38.027801349
1	8.657934521	28.418850006	38.378186938
1	8.138337747	27.876163178	36.780381273
6	11.787146610	29.874906161	38.716958289
1	11.816190193	28.821648681	39.018993449
1	12.791459438	30.265324969	38.888662425
6	10.794955040	30.625921272	39.637824715
1	11.093569622	30.518718258	40.687950820
1	10.766030079	31.692889155	39.395684898
1	9.778184626	30.236351180	39.524715013
6	14.481603809	34.608986733	35.752369940
1	14.799907614	35.657628175	35.731282262
1	14.716218399	34.243040129	36.753560928
6	15.321944018	33.816843321	34.722926746
1	16.391889379	33.912053223	34.944093182
1	15.063924698	32.752561607	34.743511403
1	15.147939698	34.184764195	33.705502922
6	12.800324377	36.816327480	34.301866326
1	12.130948773	37.648097258	34.525753994
1	13.745320157	37.043890529	34.807938791
6	13.053205586	36.776991306	32.776136021
1	13.463630520	37.732961097	32.428701854
1	13.762378007	35.983428932	32.514485863
1	12.122614987	36.588095540	32.230499772
1	5.135855893	30.520086499	32.275343729
6	9.928971543	27.389426158	34.034813382
6	8.728211641	26.661356275	34.057472252
1	7.785849320	27.198259677	34.115248526
6	8.730841253	25.257572522	34.013676795
6	11.182010353	25.293380564	33.904137260
6	11.149223534	26.694736170	33.959097466
1	12.078274949	27.257254182	33.945155012
1	13.267393359	25.139222375	33.329331126
6	13.384313679	32.298365715	37.544328440
6	13.291754232	33.182088610	38.631031045
1	12.431309158	33.840661118	38.705239325
6	14.285084917	33.215828726	39.624730033
6	15.382626234	32.349891660	39.505333003
1	16.154487937	32.363875889	40.271665024
6	15.506119462	31.458861300	38.423430000
6	14.495456589	31.439897863	37.452604306
1	14.561894888	30.748537977	36.617725441
6	16.709220913	30.546000172	38.315117326
1	16.914921643	30.041762510	39.266924133
1	16.558048137	29.776033228	37.552181843
1	17.613284058	31.107632310	38.044696408
6	14.174316183	34.179329614	40.787717101
1	13.140207330	34.261076897	41.140606226
1	14.795663591	33.861460056	41.631235822
1	14.499646583	35.188684311	40.500912152
6	9.697454515	37.156271786	34.413102643
6	9.557406429	37.939099975	33.253539537
1	9.467455181	37.446086424	32.289934148
6	9.525621995	39.339930718	33.325052175
6	9.631296933	39.950736652	34.586832584
6	9.769054534	39.195223948	35.762530526
6	9.806508178	37.795033556	35.659875460
1	9.912970772	37.190969404	36.555645455
1	6.731924188	24.898201970	34.771829725
6	9.893946785	39.873981898	37.110780951
1	10.946425083	40.040578599	37.378200639
1	9.398045772	40.850595109	37.113702765
1	9.449229797	39.265786608	37.905797923
6	9.346573075	40.178291410	32.076856660
1	8.298870275	40.483912676	31.950426551
1	9.947687062	41.093516725	32.119195165
1	9.636706639	39.624075775	31.178646869
1	9.601174411	41.035868652	34.654771022

^{4}1{B3LYP}$_{optBS3}$

6	8.157967568	30.069189735	31.173103182
6	7.681116007	30.045273894	29.739319460
1	7.685969804	29.009843576	29.381524091
1	6.646713598	30.384582803	29.657705850
6	8.561198993	30.894184747	28.799657230
1	8.191349787	30.838224294	27.767564022
1	8.559588740	31.947292148	29.102001452
1	9.601891263	30.545998613	28.807921344
6	9.419729210	27.836126983	31.085487145
1	8.759980661	27.611748504	30.240678537
6	10.862952196	27.965337457	30.560596120
1	11.173299865	27.042860014	30.055057665
1	10.954442700	28.792088374	29.846206785
6	6.015951167	32.227578765	31.146222889
6	5.963968082	33.070505397	30.030038808
1	6.776959977	33.773136190	29.857411403
6	4.888703694	33.016775260	29.132736769
6	3.856480855	32.105629292	29.382096824
1	3.017585849	32.051507369	28.688831049
6	3.873016252	31.256942448	30.498366984
6	4.965953181	31.322589188	31.367368726
6	4.840628423	33.939648769	27.934345163
1	5.832167188	34.064950025	27.484097130
1	4.163717943	33.558545703	27.161618220
1	4.485805778	34.940355409	28.216657024
6	2.728406622	30.301696983	30.754370654
1	1.850594706	30.830630757	31.150164575
1	2.412670691	29.796454924	29.833411384
1	3.004822458	29.534138329	31.484265046
6	10.008423052	24.596917894	34.034154875
1	10.051142528	23.508386534	34.004033488
6	12.530597783	24.629208427	33.813794044
1	12.968171202	24.435540217	34.803402899
1	12.429613979	23.663531241	33.307951127
6	7.492390379	24.409897004	34.260588595
1	7.699382115	23.363818283	34.518075168
1	6.947481871	24.411385996	33.301599840
26	9.532200662	32.248765777	34.538802209
7	8.566465052	33.647795645	33.531459994
7	8.838394020	30.888172496	33.228101326
7	10.733745721	30.864289384	35.272779210
7	10.848637067	33.645498266	35.140888921
17	7.985382550	32.149936222	36.306651516
6	8.505894561	34.954329722	33.985742540
6	7.157877049	35.470468601	33.814696715
6	6.438590730	34.493816845	33.154206668
6	7.350488458	33.385435056	32.921097494
6	7.175896824	32.268002240	32.089018268
6	8.051991661	31.159830448	32.129471488
6	8.930674552	29.090332695	31.770766928
6	9.290516037	29.590869125	33.090039996
6	9.847145928	28.878696450	34.172220710
6	10.360657759	29.530370637	35.309515326
6	10.752124863	28.951128269	36.583021894
6	11.463747236	29.924137741	37.261367164
6	11.499397124	31.096081956	36.403498573
6	12.291075402	32.252953242	36.530131208
6	12.064150485	33.404221566	35.747652415
6	12.930749463	34.561060658	35.573782391
6	12.176049139	35.531517463	34.943690264
6	10.848581817	34.968096852	34.748671948
6	9.661576633	35.643414482	34.388399120
6	6.568941552	36.740390082	34.382724797
1	5.635247567	36.964247598	33.855999488
1	7.222747541	37.596016185	34.208733012
6	6.273922611	36.638462621	35.894056257
1	5.826433255	37.572084518	36.260000964
1	5.581100013	35.816710450	36.110852147
1	7.190632090	36.453591431	36.463757381
6	4.950686358	34.542176485	32.900211297
1	4.622063672	35.586998933	32.914320863
1	4.704058466	34.163855920	31.906949376
6	4.140425859	33.754945603	33.951875952
1	3.065803508	33.823071917	33.736761883
1	4.420913581	32.696726251	33.955361224
1	4.314941959	34.144579856	34.961225530
1	9.363137799	26.971626296	31.748767012
1	11.562747206	28.153611015	31.380754328
6	10.333045438	27.619709798	37.164108404
1	10.449787346	26.812714269	36.438437200
1	10.998618294	27.371521078	37.998672783
6	8.879896864	27.625623679	37.679648488
1	8.621732706	26.647870861	38.106917684
1	8.735415054	28.388600836	38.452871451
1	8.175255359	27.842015589	36.870829349
6	11.953036884	29.800925156	38.684619859
1	12.028313154	28.738508223	38.943215262
1	12.959206740	30.207099854	38.795841817
6	11.019694135	30.492082421	39.699427406
1	11.405840469	30.368314987	40.719298460
1	10.935951401	31.564442453	39.492493919
1	10.009312354	30.071306145	39.661735128
6	14.411944878	34.665438564	35.851828698
1	14.691392425	35.723558800	35.900256871
1	14.662691321	34.246849041	36.826921205
6	15.273776232	33.978277665	34.773542827
1	16.340822614	34.086669731	35.007304437
1	15.046869792	32.908126024	34.710537926
1	15.096220281	34.415461761	33.783252631
6	12.716193638	36.837456807	34.410953173
1	12.051141038	37.669273090	34.645217889
1	13.664519457	37.060900984	34.911184793
6	12.959749311	36.806024942	32.887855451
1	13.374869578	37.762134247	32.542979875
1	13.663289106	36.010886662	32.614210314
1	12.026521383	36.626859839	32.343305548
1	5.012756630	30.661597126	32.230112459
6	9.897849222	27.384217570	34.113794976
6	8.725162343	26.623000461	34.201890164
1	7.770555226	27.132046737	34.300965816
6	8.766304039	25.223474299	34.170883530
6	11.198781361	25.331197872	33.935288882
6	11.127175846	26.725919956	33.982205478
1	12.037981827	27.316580643	33.920606970
1	13.251358073	25.232266765	33.251279993
6	13.419206978	32.254437870	37.513268719
6	13.362036472	33.041243288	38.669675181
1	12.482574174	33.657039905	38.849413623
6	14.412071295	33.036831763	39.599448704
6	15.530540521	32.237373902	39.339799428
1	16.352672929	32.226147845	40.054814586
6	15.622457101	31.447005015	38.184429137
6	14.552833559	31.457148232	37.285668620
1	14.590866573	30.836628259	36.393054406
6	16.859217504	30.615974165	37.920312136
1	17.203771840	30.107788608	38.829554564
1	16.673960182	29.853804787	37.156076981
1	17.690392715	31.241917317	37.567261793
6	14.334764075	33.890824779	40.846190706
1	13.361549728	33.789746498	41.340719235
1	15.110726903	33.615369915	41.567781338
1	14.465859962	34.955649593	40.609979454
6	9.628043991	37.137588755	34.448460545
6	9.530896349	37.896247475	33.272751174
1	9.473164650	37.382138146	32.317457098
6	9.497099838	39.293222389	33.312997858
6	9.556556099	39.926323410	34.560780801
6	9.649609561	39.197591315	35.750179735
6	9.688645623	37.800577584	35.676583041
1	9.763481661	37.214318028	36.589302088
1	6.815112110	24.815940934	35.020731618
6	9.734346325	39.898509652	37.086985083
1	10.773360914	39.980352500	37.427318880
1	9.329150184	40.914031869	37.034868755
1	9.183663565	39.353423478	37.860985137
6	9.365833757	40.108171899	32.047051802
1	8.335440909	40.455359616	31.904336968
1	10.004252281	40.999246429	32.072517711
1	9.639329044	39.522198020	31.166343655

1	9.527837765	41.014355352	34.604863737

^{6}1{B3LYP}$_{optBS1}$

6	8.167565783	30.070667871	31.128567828
6	7.698986482	30.046579823	29.692699579
1	7.722138368	29.014275483	29.325327931
1	6.660302666	30.370035623	29.610262196
6	8.568986836	30.920699318	28.758739016
1	8.202773679	30.863235753	27.726556960
1	8.545525639	31.970474141	29.070107353
1	9.613827908	30.591208379	28.770517275
6	9.469141700	27.856838498	31.025589529
1	8.829710838	27.638737283	30.162333451
6	10.928247971	28.006936179	30.531853961
1	11.256280929	27.094708848	30.018258316
1	11.024464586	28.846518742	29.833978219
6	5.985143317	32.200566747	31.119644345
6	5.868180641	33.046686781	30.005743460
1	6.647044187	33.778428093	29.811415770
6	4.765130766	32.955975858	29.139535840
6	3.772743023	32.002110454	29.413364927
1	2.916658035	31.922081669	28.747110074
6	3.858758446	31.145540102	30.526035688
6	4.974131015	31.253128873	31.367489803
6	4.642121893	33.886080077	27.950671359
1	5.620627691	34.097986142	27.505977513
1	4.001878419	33.457871414	27.172438057
1	4.202798894	34.849710974	28.243155130
6	2.769872178	30.129228447	30.800053446
1	1.803472684	30.617774463	30.978945512
1	2.634998403	29.448719614	29.949792511
1	3.003827242	29.522646793	31.680097169
6	9.967303274	24.565831400	33.959147173
1	9.988483206	23.479271287	33.918260718
6	12.500555887	24.545256519	33.797447363
1	12.911384595	24.328836956	34.793070404
1	12.389932860	23.588291646	33.276131516
6	7.435648955	24.441342381	34.127035919
1	7.613180034	23.388027703	34.367218645
1	6.909019377	24.476719953	33.163592316
26	9.454148411	32.254842569	34.608175881
7	8.555019506	33.712325265	33.447812239
7	8.841637756	30.885455459	33.191261763
7	10.806124885	30.826639432	35.248957267
7	10.874693406	33.667389750	35.104930121
17	7.910361797	32.160759958	36.373344797
6	8.512363402	35.024808888	33.906988978
6	7.152995438	35.539534634	33.730529563
6	6.425908636	34.555722647	33.073027039
6	7.336409404	33.433813672	32.835984523
6	7.165289168	32.281181789	32.039606673
6	8.043780026	31.167151293	32.087754136
6	8.953973565	29.093289296	31.723810166
6	9.311889354	29.579997416	33.055670404
6	9.885780962	28.860623267	34.132555831
6	10.432015015	29.488204651	35.275521399
6	10.820717667	28.916574799	36.565015942
6	11.517707445	29.901677187	37.253549097
6	11.568465791	31.077154387	36.385083393
6	12.334853039	32.257805094	36.501613164
6	12.102342808	33.409762803	35.709928906
6	12.974094029	34.565343245	35.570294751
6	12.213703438	35.546123286	34.884288161
6	10.870018893	34.999428587	34.702758833
6	9.682063496	35.685605913	34.341897681
6	6.563020038	36.809386967	34.297051121
1	5.643492295	37.051970512	33.751407636
1	7.232951335	37.656863228	34.147610676
6	6.228319227	36.698153118	35.804128237
1	5.791175555	37.635824398	36.168870933
1	5.515011819	35.889053176	35.992293426
1	7.128477708	36.489031779	36.390955617
6	4.935864747	34.605166522	32.830520108
1	4.603428701	35.649533358	32.854544339
1	4.684672681	34.232209712	31.836611641
6	4.126630963	33.806404087	33.880742519
1	3.052064636	33.886509402	33.675694089
1	4.399719165	32.746683395	33.863690817
1	4.313655460	34.180524582	34.892688628
1	9.405723693	26.982392391	31.673928273
1	11.608656451	28.188801774	31.370860375
6	10.410965780	27.585340761	37.148304574
1	10.518761543	26.785378863	36.415124966
1	11.087460170	27.331652909	37.972916087
6	8.958286770	27.581123791	37.681926678
1	8.708765986	26.599973547	38.104342074
1	8.821263811	28.337212501	38.462083663
1	8.245460253	27.800192601	36.880365947
6	11.956007207	29.800916999	38.695059077
1	12.013197394	28.744005515	38.979915178
1	12.960280096	30.203956797	38.831879411
6	10.992218168	30.522193904	39.667958816
1	11.336424899	30.405726751	40.702956019
1	10.936859170	31.592221517	39.444084375
1	9.978079895	30.116120584	39.593450409
6	14.463464550	34.647294914	35.745960933
1	14.769914763	35.699657437	35.744778182
1	14.727918881	34.258994665	36.730590001
6	15.287850593	33.892913404	34.675740795
1	16.361460276	33.992186312	34.876749770
1	15.039958523	32.826055723	34.670597939
1	15.089140071	34.288189129	33.673216111
6	12.756517402	36.847902164	34.345114440
1	12.088738855	37.678724412	34.575984737
1	13.704960590	37.075846883	34.844895710
6	13.000396795	36.813103479	32.817494149
1	13.405690084	37.771341534	32.470637018
1	13.711383356	36.023214842	32.549199755
1	12.067647745	36.622224617	32.276057701
1	5.067580622	30.595672477	32.226466631
6	9.913405818	27.363858692	34.064412957
6	8.719344452	26.626432368	34.119859394
1	7.774760272	27.155918475	34.203853541
6	8.731646343	25.222623051	34.074139839
6	11.178426249	25.277151066	33.896323885
6	11.136334293	26.677828896	33.955459543
1	12.060312871	27.247575948	33.917508155
1	13.246999593	25.139922680	33.260108227
6	13.442976501	32.297420127	37.507982632
6	13.367956614	33.162583009	38.611115061
1	12.497623154	33.802582809	38.723045731
6	14.393528992	33.204930275	39.570827440
6	15.505136840	32.365463251	39.401710283
1	16.302758666	32.386757378	40.140956595
6	15.611190781	31.492922940	38.303241869
6	14.568526291	31.464619800	37.367199355
1	14.622365230	30.787948270	36.519520293
6	16.831112940	30.611371927	38.138868881
1	17.091047189	30.109422316	39.078488901
1	16.666558832	29.840590728	37.379535707
1	17.707905470	31.197054463	37.831810986
6	14.304309363	34.152430543	40.748711576
1	13.277515234	34.227884069	41.123413920
1	14.943352279	33.824683959	41.575139671
1	14.622573481	35.166062402	40.468816311
6	9.670030529	37.179671843	34.457201587
6	9.557178898	37.991854932	33.314649355
1	9.471970462	37.523609771	32.338830986
6	9.547894049	39.390751140	33.417345850
6	9.644387188	39.971081954	34.694128664
6	9.751466489	39.186709892	35.853848134
6	9.768756681	37.788694968	35.719820886
1	9.854378697	37.162864578	36.603146583
1	6.754807005	24.850041748	34.882296755
6	9.869534759	39.833137480	37.218222556
1	10.920449797	39.997050413	37.492383853
1	9.370596249	40.807834871	37.243225923
1	9.424957563	39.204446272	37.996790352
6	9.403947794	40.260055494	32.185704283
1	8.367000922	40.599426963	32.057556975
1	10.031641708	41.156082875	32.252067538
1	9.685365586	39.716446821	31.278233591
1	9.632277593	41.054836452	34.786360108

^{6}1{B3LYP}$_{optBS2}$

6	8.140485437	30.046498789	31.181135125
6	7.672996774	29.997124575	29.745667656
1	7.680631398	28.955801141	29.405759602
1	6.639807960	30.331383065	29.653666479
6	8.556174269	30.828977657	28.794660486
1	8.184374898	30.760146547	27.764743451
1	8.561025846	31.885604725	29.082823890
1	9.593811923	30.476364915	28.806222789
6	9.435974570	27.835140432	31.103940397
1	8.794952403	27.605463808	30.245790751
6	10.886575894	27.989502469	30.605237032
1	11.220428606	27.073238381	30.102790550
1	10.976491635	28.819467457	29.895393283
6	6.028909321	32.225426091	31.095611908
6	5.986953330	33.039803671	29.958446497
1	6.806510608	33.729987671	29.770140352
6	4.912944936	32.974266553	29.061299471
6	3.870497297	32.081731423	29.332304837
1	3.031326822	32.020688969	28.640379936
6	3.877314322	31.261578743	30.469401894
6	4.969781181	31.337405032	31.336664774
6	4.875961738	33.866206274	27.840376722
1	5.862817846	33.942779309	27.369707536
1	4.171851954	33.489893419	27.090811841
1	4.563401698	34.886166274	28.102356043
6	2.720645008	30.329347953	30.752474355
1	1.856440218	30.879093699	31.150011461
1	2.385663850	29.816393018	29.842664500
1	2.992474957	29.567439178	31.490445355
6	9.986096015	24.597715199	34.011359986
1	10.020204598	23.509716624	33.971140959
6	12.507732582	24.606150433	33.768901717
1	12.954752409	24.402856846	34.751299568
1	12.392262624	23.643168958	33.259173025
6	7.468171717	24.438244227	34.256466336
1	7.665323894	23.390229401	34.505172317
1	6.920112199	24.453857425	33.304847798
26	9.408819977	32.260440715	34.673439260
7	8.549079707	33.711571576	33.460239953
7	8.800636443	30.876775063	33.235795038
7	10.799793387	30.838182745	35.269447184
7	10.837280459	33.690611044	35.186522365
17	7.896393378	32.163729493	36.395509424
6	8.491562303	35.009332054	33.914589236
6	7.149292521	35.541211517	33.696936952
6	6.439133733	34.564562536	33.027061737
6	7.350991305	33.440965585	32.833816874
6	7.179829531	32.282092676	32.048152440
6	8.028295750	31.149057975	32.130153462
6	8.916254350	29.077793661	31.787602129
6	9.265036577	29.586067310	33.107748636
6	9.858691708	28.879548519	34.179905091
6	10.424933590	29.513324547	35.306380754
6	10.843911616	28.924421812	36.571493972
6	11.570275589	29.892377894	37.238035327
6	11.574350484	31.074694804	36.381788025
6	12.324015406	32.265544813	36.516880595
6	12.054967683	33.445491746	35.784920037
6	12.908823685	34.619287747	35.632793807
6	12.142120185	35.593253977	35.024174621
6	10.821181691	35.013161103	34.804886627
6	9.642874680	35.677465366	34.387041433
6	6.551057562	36.819916694	34.234819269
1	5.640967985	37.050976645	33.670276124
1	7.219778793	37.667050156	34.081916889
6	6.191660936	36.730536189	35.731735026
1	5.751349828	37.674572670	36.076217376
1	5.471518837	35.927559425	35.921397756
1	7.079001637	36.526888947	36.339813814
6	4.958111276	34.627865257	32.736091023
1	4.642713425	35.677018965	32.722159786
1	4.730672637	34.234447707	31.745443506
6	4.110128313	33.873313773	33.780335445
1	3.043340302	33.953116752	33.536675055
1	4.373709942	32.811192974	33.810574746
1	4.262747228	34.279521022	34.785844354
1	9.378804539	26.965815740	31.759217621
1	11.570813811	28.187756018	31.437195448
6	10.427198821	27.596847981	37.158028272
1	10.525754574	26.790851833	36.430105820
1	11.104532625	27.342138696	37.980201931
6	8.982334114	27.609578245	37.696728933
1	8.727272656	26.634046526	38.129072626
1	8.853212818	28.372493247	38.471889976
1	8.264574892	27.826738211	36.898942773
6	12.092744652	29.754809429	38.648752290
1	12.198077138	28.689953061	38.884727268
1	13.091836296	30.180800486	38.744314881
6	11.168759792	30.401992948	39.699930499
1	11.585529664	30.274042352	40.706831420
1	11.049181807	31.474480390	39.514448055
1	10.170418973	29.951717782	39.683315746
6	14.390795420	34.738955893	35.902364820
1	14.656783393	35.799843892	35.963115455
1	14.655518157	34.310150316	36.868572886
6	15.255079558	34.078476294	34.809477805
1	16.321517380	34.195452144	35.039061519
1	15.041601268	33.007249242	34.731150649
1	15.067374900	34.527920276	33.827957463
6	12.672637232	36.920310303	34.533314084
1	11.976795195	37.731657023	34.747289778
1	13.590168456	37.160935216	35.081304799
6	12.989104077	36.919974481	33.023885121
1	13.381202047	37.896159079	32.712566627
1	13.735748256	36.157792413	32.773956870
1	12.091519582	36.712444313	32.431737587
1	5.007810170	30.698805836	32.216192354
6	9.899775880	27.386240829	34.116001493
6	8.721968529	26.634880474	34.208123159
1	7.771975245	27.152214971	34.318025846
6	8.750020315	25.235796381	34.164445007
6	11.181973316	25.320965216	33.909116283
6	11.122821344	26.716144753	33.970136434
1	12.039193559	27.297901821	33.906957074
1	13.228515661	25.205568224	33.202193294
6	13.480894913	32.251578757	37.464531589
6	13.451942042	32.987132389	38.653844834
1	12.575332416	33.588692534	38.884131154
6	14.525898431	32.947734019	39.553618169
6	15.640468261	32.168023811	39.230126524
1	16.479650712	32.129344840	39.923552189
6	15.705166199	31.430020802	38.039442810
6	14.611618809	31.473207677	37.172134379
1	14.626987748	30.890903097	36.253556019
6	16.936025502	30.617726114	37.704443136
1	17.306648236	30.070591863	38.579244602
1	16.732095127	29.890058443	36.912271431
1	17.754715490	31.262124365	37.357047233
6	14.479258038	33.743760441	40.838153877
1	13.508868711	33.641313966	41.337186732
1	15.255503311	33.419386907	41.538667981
1	14.632238634	34.813941945	40.647519593
6	9.614436466	37.170665062	34.437954449
6	9.574367542	37.918159158	33.251433267
1	9.564542097	37.394830413	32.297986806
6	9.540321769	39.314198242	33.276734828
6	9.539473659	39.959293279	34.521931684
6	9.576757564	39.242924254	35.722657374
6	9.618215857	37.843858769	35.664574358
1	9.653130768	37.265896107	36.585481416
1	6.797415441	24.846895764	35.020863368
6	9.597388856	39.955930163	37.056525927
1	10.610026240	39.973884984	37.482376678
1	9.262098602	40.994047624	36.961478995
1	8.950229195	39.456231115	37.786939741
6	9.473702901	40.115289984	31.995595420
1	8.450534494	40.461490189	31.796449556
1	10.111040863	41.005893141	32.045022145
1	9.792045786	39.519291761	31.134224229
1	9.508198293	41.047700520	34.554491698

^{2}2{B3LYP}$_{optBS1}$

7	5.905971921	6.723910962	11.362968795
7	8.638410947	6.355582230	11.316217274
6	6.148113227	7.109457502	10.040752770
6	4.979654498	7.821291505	9.536562012
6	3.987586725	7.706768208	10.499938344
6	4.554219010	6.949582614	11.609760080
6	3.886016249	6.358685179	12.706153362
6	9.944521378	5.925806015	11.573908278
6	10.438186177	5.183775662	10.418740675

6	9.482439295	5.305603958	9.418841469
6	8.382756514	6.086707435	9.972093411
6	7.261330320	6.634118218	9.320581185
6	11.658774198	4.298090576	10.341403094
1	12.535094337	4.790575102	10.763171890
1	11.895767038	4.106433497	9.288882406
6	11.457710789	2.939177934	11.055709592
1	12.357261212	2.320341332	10.954130529
1	10.612432236	2.389018353	10.630135060
1	11.259825449	3.078956042	12.122995670
6	9.552002269	4.610362331	8.079990238
1	10.601379735	4.392565594	7.849740122
1	9.200402009	5.263236352	7.280566960
6	8.751845030	3.287039326	8.035024371
1	8.886210947	2.796326523	7.063567123
1	7.682286408	3.465812722	8.175591582
1	9.073733091	2.593073220	8.817541405
6	4.886946961	8.665924639	8.288836999
1	3.833313770	8.883841541	8.081911900
1	5.258903440	8.124532266	7.418007590
6	5.651842711	10.005042651	8.410799141
1	5.537734678	10.597191083	7.494817567
1	6.721242832	9.832975662	8.572738366
1	5.275722019	10.599103014	9.251595295
6	2.641933460	8.388458745	10.447933562
1	1.849476537	7.713555154	10.773712317
1	2.410471064	8.645081041	9.408061948
6	2.584333686	9.679047335	11.298828775
1	1.595268704	10.146664660	11.222278023
1	3.333253580	10.405760757	10.963836561
1	2.776773567	9.462030204	12.355087982
6	7.211063471	6.653736329	7.823352414
6	6.337068098	5.786581225	7.143772741
1	5.710345963	5.108760046	7.713851343
6	6.282125372	5.766401947	5.742605450
6	7.108348582	6.650803245	5.026375818
1	7.075932837	6.644176233	3.938931384
6	7.982439897	7.535601658	5.678219326
6	8.032512659	7.518395729	7.082827749
1	8.717214173	8.180585245	7.605061911
6	5.357934945	4.810554998	5.016647456
1	5.902703140	4.224256835	4.266346141
1	4.561259792	5.350161793	4.487953562
1	4.884439262	4.109799654	5.710595471
6	8.839701702	8.499717666	4.884627467
1	9.759528275	8.753208377	5.422192268
1	8.305244449	9.439858390	4.690069756
1	9.120032177	8.078202285	3.913081679
6	2.404224092	6.160711268	12.607607531
6	1.896440616	5.225464070	11.687301686
1	2.586729076	4.652733115	11.077233877
6	0.517078619	5.001702976	11.569639663
6	-0.354471077	5.746990302	12.384005111
1	-1.426696614	5.582374665	12.303011757
6	14.399511695	6.703076236	12.108973493
6	13.010421446	6.890626576	12.005305947
1	12.615309298	7.603611735	11.287361915
6	-0.024232858	3.972722253	10.598911124
1	0.781020365	3.493604059	10.034387090
1	-0.717913927	4.427406748	9.880094670
1	-0.576333323	3.183419790	11.124696168
6	15.353993441	7.510717239	11.254091449
1	16.279098537	6.958041597	11.058061507
1	15.633930219	8.451262615	11.747568482
1	14.904300375	7.771051737	10.289895937
8	6.602426843	1.577200648	10.455537874
6	5.512385000	1.826743431	9.648989654
6	4.830347346	3.056499392	9.641813085
1	5.149676057	3.852776758	10.306557514
6	3.748089283	3.239873244	8.769605236
1	3.232714577	4.196477006	8.758386136
6	3.335428261	2.210797040	7.912119824
1	2.495333466	2.358117055	7.240747660
6	4.019527077	0.985146413	7.934793837
1	3.707584394	0.178059719	7.278394299
6	5.101999739	0.790623141	8.794470031
1	5.644379491	-0.147291579	8.824944386
1	6.806954020	2.322131777	11.071766924
26	7.261503255	6.280238538	12.717710963
17	7.184344063	3.834072653	12.601676723
7	8.621533414	6.713001286	14.062704486
7	5.883659736	6.362728288	14.107773363
6	8.376255156	7.115518569	15.381051733
6	9.542137419	7.834073952	15.877732298
6	10.535206308	7.715545072	14.913791899
6	9.971988943	6.946861333	13.812779753
6	10.639456350	6.355920322	12.715933880
6	4.578935870	5.929393394	13.850470301
6	4.088047472	5.186892271	15.004427825
6	5.045428885	5.310430446	16.004798533
6	6.141004988	6.095650640	15.452994136
6	7.261900441	6.646673521	16.104883158
6	2.871957334	4.295257884	15.081142415
1	1.994514121	4.782283079	14.655528461
1	2.632931018	4.104976872	16.133504794
6	3.082420440	2.936107699	14.369603470
1	2.186752306	2.311659124	14.471643280
1	3.931224750	2.391851134	14.795876230
1	3.280497637	3.075800660	13.302349123
6	4.981198833	4.612459864	17.342579569
1	3.933784951	4.385049893	17.572058084
1	5.327148297	5.267617680	18.143020418
6	5.793611088	3.296448452	17.386223568
1	5.659555813	2.800923944	18.355292020
1	6.862232206	3.486245617	17.251151792
1	5.482044790	2.602527632	16.599404931
6	9.634569417	8.687481315	17.119511967
1	10.688527495	8.899848022	17.330777546
1	9.253643459	8.155186637	17.991969502
6	8.878332188	10.030308820	16.982617544
1	8.989125641	10.628155322	17.895301600
1	7.809104212	9.862755111	16.813867794
1	9.263656166	10.616446331	16.140439422
6	11.879789917	8.399651310	14.963104131
1	12.672745691	7.723897006	14.640305151
1	12.110674381	8.660511903	16.002110142
6	11.937304599	9.686591583	14.106853555
1	12.926012051	10.155041976	14.182610836
1	11.187732743	10.414515717	14.437596581
1	11.746354236	9.464829108	13.051324921
6	7.307667129	6.676600557	17.602186177
6	8.184128956	5.819562415	18.290876969
1	8.816672182	5.141203298	17.727365565
6	8.234165471	5.809333565	19.693034004
6	7.399593159	6.691342894	20.400967244
1	7.427546866	6.691065455	21.488394986
6	6.521719027	7.566277555	19.739422165
6	6.477496945	7.540713543	18.335248826
1	5.790339313	8.194963520	17.806146465
6	9.170807965	4.871731217	20.425385133
1	8.664250576	4.374879002	21.261220299
1	10.029153924	5.414140036	20.843790439
1	9.560397923	4.097374327	19.757683552
6	5.654256804	8.526831277	20.525765756
1	4.771080553	8.828738998	19.953418506
1	6.206260518	9.441058943	20.783950117
1	5.312118222	8.078628631	21.465334741
6	12.121899566	6.163304424	12.813130462
6	12.635897594	5.229162079	13.731136406
1	11.949697711	4.652048154	14.341603657
6	14.016498950	5.010304328	13.845172697
6	14.883463687	5.759645280	13.029703311
1	15.956469006	5.598458708	13.107333742
6	0.123593656	6.691216453	13.306859463
6	1.511721820	6.883961738	13.414102207
1	1.902717321	7.598171442	14.133092858
6	14.563314439	3.982380543	14.814085337
1	13.758737376	3.481329758	15.360413719
1	15.235444941	4.443590127	15.549232964
1	15.140278953	3.210256865	14.289629243
6	-0.835834531	7.495134597	14.159656968
1	-1.759895553	6.939572685	14.352289862
1	-1.116921791	8.435403259	13.666293244
1	-0.389640849	7.755747407	15.125423062
8	7.821767710	1.629311872	14.782507679
6	8.903283267	1.865344386	15.603754261
6	9.583896625	3.095501044	15.642779967
1	9.272022827	3.905486579	14.991043626
6	10.655575733	3.262976038	16.531688218
1	11.170045576	4.219520264	16.567906374
6	11.060232149	2.217846514	17.373360329
1	11.892293703	2.353157307	18.057235068
6	10.378565261	0.991925882	17.318358190
1	10.684437283	0.172174771	17.961779001
6	9.306206350	0.813304804	16.442813496
1	8.765791884	-0.124591286	16.387422001
1	7.621502599	2.379788188	14.171059269

^{2}2{B3LYP}$_{\text{optBS2}}$

7	5.750525427	6.853578759	11.518166835
7	8.499763535	6.537230509	11.173907801
6	5.820116080	7.010885373	10.169341018
6	4.636676652	7.714057375	9.683252830
6	3.810270784	7.886786372	10.771243873
6	4.477166148	7.248485217	11.903362559
6	3.913264561	6.826987749	13.113638350
6	9.830747880	6.281865028	11.283813298
6	10.288945322	5.491351060	10.144610095
6	9.191969426	5.312470753	9.324465001
6	8.094454367	6.046878283	9.937895861
6	6.854510938	6.398853406	9.395128273
6	11.636497054	4.848189413	9.911496740
1	12.445363673	5.515531221	10.204809732
1	11.763551559	4.685797813	8.834926506
6	11.808839761	3.498020537	10.635453614
1	12.805630237	3.084466460	10.438037464
1	11.066598411	2.766869365	10.298700584
1	11.695213949	3.604052202	11.718660434
6	9.156657855	4.378940883	8.137238232
1	10.182856402	4.194026645	7.801983780
1	8.638658861	4.824688515	7.288936504
6	8.498033070	3.022584388	8.465009738
1	8.524241437	2.366894231	7.586067022
1	7.452874200	3.148811386	8.762517654
1	9.016179882	2.516350227	9.285971345
6	4.402870070	8.289277957	8.308019547
1	3.341273751	8.537865991	8.204093478
1	4.612124966	7.548846732	7.534947738
6	5.234384335	9.557011764	8.033284477
1	5.024265544	9.948129621	7.030197124
1	6.308123610	9.348533167	8.096287402
1	5.005696179	10.344959629	8.760400785
6	2.554149144	8.723325150	10.789076570
1	1.733366979	8.199417136	11.279973254
1	2.227552836	8.896989736	9.758402978
6	2.761192445	10.087006863	11.477046611
1	1.838379810	10.679124788	11.444511169
1	3.553475558	10.662867493	10.984664435
1	3.044376706	9.959781013	12.527644241
6	6.527650170	6.053293232	7.978737412
6	5.573979069	5.050901661	7.743431042
1	5.103055296	4.566074754	8.595268648
6	5.245453578	4.662986029	6.441335921
6	5.873770448	5.317938550	5.373030659
1	5.627842180	5.023793011	4.353136788
6	6.812068498	6.335868936	5.578051411
6	7.139829519	6.689482266	6.893399758
1	7.883677553	7.461906888	7.076155736
6	4.232488823	3.567999243	6.187604125
1	4.618970477	2.823577799	5.480303443
1	3.309627777	3.974464477	5.752332584
1	3.963093770	3.047926179	7.112176513
6	7.452080556	7.044680261	4.404870238
1	8.478226797	7.354034192	4.632571734
1	6.892677503	7.949978241	4.133658969
1	7.480985487	6.402475931	3.517737097
6	2.428067410	6.855331809	13.255611556
6	1.659026085	5.963024610	12.494362183
1	2.166896539	5.291125402	11.807336799
6	0.270435119	5.910465443	12.638134289
6	-0.339650101	6.787456763	13.546333420
1	-1.421109813	6.750204453	13.672756733
6	14.044533545	7.872513750	11.147491970
6	12.656822636	7.803157726	11.314892927
1	12.010437177	8.472784660	10.751180467
6	-0.554670493	4.934304736	11.829332864
1	0.063207895	4.118112546	11.441391324
1	-1.028756137	5.429336589	10.970804562
1	-1.358374173	4.494383105	12.431494781
6	14.661958235	8.899346874	10.224042678
1	15.626298148	8.557132571	9.832549549
1	14.840313233	9.849317384	10.746244155
1	14.007920281	9.115850297	9.371934355
8	9.455074583	3.830867547	14.547405388
6	10.195108280	2.696510022	14.540114028
6	9.823764305	1.544567755	13.827900665
1	8.909855596	1.552356525	13.241918705
6	10.629356692	0.407735002	13.880639528
1	10.330451253	-0.478930358	13.324986632
6	11.805685035	0.393923421	14.634035783
1	12.426393307	-0.497795650	14.670588073
6	12.174116793	1.543420174	15.337208339
1	13.089017919	1.550151876	15.926108002
6	11.380747639	2.688982957	15.293362175
1	11.661404021	3.590138277	15.832841369
1	8.669766659	3.778108965	13.924886524
26	7.257273382	6.493172360	12.739750382
17	7.181558118	3.940481034	12.715811454
7	8.766898338	6.812480093	13.966623366
7	6.011142447	6.534477166	14.306350483
6	8.698871449	6.943918594	15.318100183
6	9.891919736	7.621366525	15.818936199
6	10.719277545	7.809173849	14.734903250
6	10.045464526	7.198924978	13.589188577
6	10.602549570	6.806807002	12.367509268
6	4.681035745	6.275475160	14.185253622
6	4.226075907	5.439164614	15.293173828
6	5.322912050	5.239638440	16.108428630
6	6.414997914	6.002454479	15.523614672
6	7.655146220	6.333684736	16.079518915
6	2.889295295	4.761713608	15.487718254
1	2.068200472	5.417661639	15.201565726
1	2.751244729	4.561038561	16.556520850
6	2.765290984	3.433281483	14.715312545
1	1.767939386	2.999476045	14.856751002
1	3.504851015	2.702778041	15.058774836
1	2.922794379	3.580889017	13.642761218
6	5.382978350	4.249936235	17.247959774
1	4.362599250	4.015114950	17.570276788
1	5.886547084	4.669473377	18.119117455
6	6.087279527	2.935971318	16.845324330
1	6.126539257	2.247717933	17.698736752
1	7.111619255	3.119653165	16.507482627
1	5.557692243	2.437156316	16.026873757
6	10.134473379	8.162322092	17.206551380
1	11.201248673	8.383683353	17.318786911
1	9.905194586	7.410998593	17.963164612
6	9.330533637	9.442056182	17.505576553
1	9.548513241	9.808496914	18.516266793
1	8.252698064	9.257128062	17.438819431
1	9.576259139	10.238970703	16.793820808
6	11.979377064	8.640123515	14.738461730
1	12.798549000	8.129506547	14.232070974
1	12.307594079	8.782285227	15.773273404
6	11.776573258	10.024509560	14.092336936
1	12.702404153	10.610426787	14.140564916
1	10.988473604	10.589013713	14.603961667
1	11.490969166	9.930424084	13.039334527
6	7.952881964	5.978252393	17.499706132
6	8.890268459	4.967340195	17.757507842
1	9.364593182	4.470767410	16.916505425
6	9.184682180	4.575799824	19.066585511
6	8.535382530	5.232217382	20.120715951
1	8.752508777	4.933544131	21.145968101
6	7.607103965	6.255255073	19.893873011
6	7.315805737	6.614752814	18.571703514
1	6.579422274	7.390409401	18.372225170
6	10.183954059	3.472287348	19.337907349
1	9.794987876	2.749708665	20.065781722
1	11.117771965	3.874836994	19.752898993
1	10.435289056	2.927724469	18.422281140
6	6.938620264	6.959688381	21.053689269
1	5.941287094	7.322677007	20.782712027
1	7.523686086	7.828924600	21.381842560
1	6.833604841	6.294221595	21.917791366
6	12.084133488	6.880190083	12.198838336
6	12.912350698	6.003580386	12.911844943

1	12.461794761	5.283826416	13.591560813
6	14.300762174	6.033128985	12.743853266
6	14.847656095	6.977570210	11.865374624
1	15.928349478	7.013124487	11.732361490
6	0.401684007	7.700551512	14.305208077
6	1.794941460	7.715015790	14.159402301
1	2.394661055	8.392558606	14.763785730
6	15.185610005	5.045321241	13.470519247
1	14.768298475	4.775108580	14.446414620
1	16.192749633	5.446681717	13.627495563
1	15.288359414	4.115138902	12.896720920
6	-0.282654835	8.651433709	15.261992966
1	-1.251332606	8.259723794	15.590637799
1	-0.466547715	9.627042017	14.791737703
1	0.329248232	8.832375067	16.152866590
8	4.475027590	3.970543156	11.037282210
6	3.967676665	2.731557665	10.788327913
6	4.570458767	1.557152148	11.259250116
1	5.481074324	1.623881883	11.847860581
6	3.992445564	0.322083692	10.966365038
1	4.466476166	-0.584962244	11.335478904
6	2.820725357	0.238817894	10.211051737
1	2.377184080	-0.727705665	9.988013165
6	2.226544640	1.414039896	9.744004800
1	1.314475585	1.364503294	9.152680168
6	2.792127755	2.657290554	10.026945471
1	2.342666166	3.578228347	9.663704155
1	5.303178306	3.913288255	11.569121245

^{4}2{B3LYP}$_{optBS1}$

7	5.904156535	6.649153561	11.333873619
7	8.641341637	6.297826929	11.315709397
6	6.165775532	7.050515739	10.020036647
6	5.024746250	7.805928197	9.524588265
6	4.027337867	7.721443145	10.490478016
6	4.561852351	6.933616573	11.590116426
6	3.875107856	6.358599810	12.681475544
6	9.956148064	5.892082598	11.575328616
6	10.452165228	5.137179390	10.433333091
6	9.488245951	5.224981734	9.435196191
6	8.386896347	6.012253669	9.971130211
6	7.278520672	6.563582763	9.304478177
6	11.685134036	4.268570682	10.369707446
1	12.548891338	4.775670235	10.799820434
1	11.936332302	4.078563534	9.319944017
6	11.494893492	2.909877435	11.087449486
1	12.404307225	2.303341905	10.999871735
1	10.662416972	2.345901370	10.654780734
1	11.280804706	3.051888402	12.151449780
6	9.542867187	4.490314338	8.117332866
1	10.584174706	4.223419855	7.903807200
1	9.222416678	5.133082118	7.295470725
6	8.689138250	3.199802129	8.106071191
1	8.812184232	2.673100978	7.152312408
1	7.626488113	3.426928766	8.230119809
1	8.976021515	2.519036981	8.913467036
6	4.960151125	8.660311309	8.282246612
1	3.913251124	8.902429132	8.067483298
1	5.329277248	8.116476890	7.411893521
6	5.753963370	9.980886700	8.420787135
1	5.658868745	10.582853740	7.509172228
1	6.818164438	9.782471553	8.587565528
1	5.386095467	10.576190514	9.264299814
6	2.707368831	8.452004549	10.442739371
1	1.892150343	7.805230395	10.769896938
1	2.482817087	8.718928002	9.403855467
6	2.698689689	9.741619409	11.297224660
1	1.727612606	10.245814898	11.222157222
1	3.474026881	10.441225419	10.964432984
1	2.882560980	9.513680280	12.352761280
6	7.255916609	6.597671005	7.807941038
6	6.359120722	5.780284022	7.098404727
1	5.691756998	5.122438529	7.645947143
6	6.330584944	5.784654478	5.695118158
6	7.212609919	6.636525628	5.008789962
1	7.202124183	6.645835598	3.920777100
6	8.114555081	7.470203809	5.691407336
6	8.131709207	7.434133003	7.095164449
1	8.833820077	8.057528603	7.641598814
6	5.362264765	4.898393351	4.939540228
1	5.847081538	4.416267879	4.082749224
1	4.516736640	5.479358509	4.547358888
1	4.954637299	4.114042030	5.584245951
6	9.035319453	8.397268067	4.925901894
1	9.884191388	8.714809782	5.540029322
1	8.506636090	9.303831241	4.601380465
1	9.430885706	7.913632954	4.025338576
6	2.384925447	6.233785860	12.607898614
6	1.805690449	5.343326464	11.685426057
1	2.448410374	4.745693980	11.047735987
6	0.413410199	5.195126502	11.600238796
6	-0.397527092	5.969192650	12.449593115
1	-1.478671700	5.862948934	12.392478469
6	14.367482253	6.868325193	11.997792171
6	12.968959533	6.985849472	11.930642227
1	12.519516377	7.667748401	11.214557908
6	-0.204504305	4.207668390	10.632630562
1	0.550749838	3.770806603	9.972923111
1	-0.968850700	4.684973245	10.006548937
1	-0.693959622	3.382896573	11.167039639
6	15.257632034	7.709592047	11.107047442
1	16.197732913	7.194605483	10.881388291
1	15.515038214	8.663460627	11.586851330
1	14.764449611	7.945628721	10.158159965
8	6.433431008	1.483210038	10.572801883
6	5.383720259	1.822025313	9.747118113
6	4.797835364	3.100853704	9.735363996
1	5.164718365	3.867719808	10.409911431
6	3.749541681	3.371194700	8.844020752
1	3.308971119	4.364572294	8.829630159
6	3.274464119	2.381593294	7.972658159
1	2.460792233	2.596586433	7.286961355
6	3.861978892	1.107029274	7.999799782
1	3.501307184	0.329568457	7.332729543
6	4.910159555	0.825529802	8.877691421
1	5.378226964	-0.151454975	8.911728317
1	6.690723905	2.207449093	11.194997209
26	7.261340235	6.202144701	12.693895443
17	7.262031108	3.691013200	12.692214066
7	8.619690328	6.639125690	14.056657577
7	5.880902833	6.291790392	14.072026259
6	8.356674474	7.037745646	15.371451532
6	9.498055891	7.790819560	15.869777708
6	10.496893174	7.706474193	14.905340952
6	9.962652579	6.923209700	13.802057096
6	10.648226303	6.354245113	12.706536309
6	4.565223743	5.889699811	13.810952533
6	4.067773528	5.130392480	14.949582141
6	5.033451079	5.209839101	15.946873848
6	6.135053365	5.999587550	15.414871432
6	7.242403750	6.550414474	16.084627037
6	2.830344062	4.268048574	15.012963740
1	1.972034364	4.776088953	14.573637228
1	2.571678103	4.088356189	16.062831163
6	3.017656940	2.902709699	14.307317414
1	2.103919209	2.302381559	14.393069050
1	3.843353094	2.336979979	14.750523928
1	3.240215375	3.035382982	13.243785527
6	4.980303796	4.465082469	17.258939563
1	3.940946252	4.185425191	17.465444551
1	5.289567547	5.105839608	18.086313730
6	5.848246459	3.184062272	17.266680181
1	5.723803264	2.649108433	18.215802436
1	6.909295424	3.423693483	17.152674824
1	5.575408035	2.505983995	16.452055156
6	9.560555261	8.644155190	17.113195889
1	10.606404676	8.891224932	17.327114804
1	9.194680089	8.097659240	17.983490168
6	8.760085282	9.960735719	16.977213672
1	8.851934489	10.561111216	17.890167886
1	7.697053190	9.756964488	16.810222863
1	9.124733920	10.559553501	16.134944167
6	11.818846725	8.432973708	14.958868170
1	12.631752553	7.787269665	14.624382619
1	12.045318327	8.688824614	16.000186208
6	11.830390974	9.730890366	14.117216639
1	12.803083208	10.231307841	14.195911762
1	11.057696301	10.429558992	14.457862315
1	11.644365888	9.513766519	13.059809425
6	7.254446766	6.589547688	17.581275042
6	8.148929114	5.780100237	18.302952582
1	8.825039125	5.123373242	17.764815639
6	8.163209381	5.790634414	19.706620976
6	7.269674720	6.639990398	20.380746125
1	7.269092604	6.653351345	21.468663213
6	6.369686468	7.465719868	19.686066437
6	6.366751553	7.423924515	18.282523427
1	5.666312872	8.041074540	17.726873785
6	9.129058191	4.914072273	20.475941112
1	8.642181932	4.445238851	21.338873185
1	9.975566442	5.499096665	20.861212060
1	9.536085522	4.120408813	19.842751772
6	5.435629884	8.389768806	20.439074108
1	4.594580984	8.705152808	19.813359571
1	5.957022887	9.297697034	20.771709926
1	5.028744020	7.904484755	21.333690687
6	12.139195013	6.229125405	12.773475933
6	12.722283161	5.335595358	13.690175795
1	12.082436066	4.735795949	14.328768382
6	14.114820628	5.187146507	13.768342392
6	14.921717581	5.965187200	12.919200295
1	16.003061791	5.859289031	12.971656812
6	0.152657881	6.868537936	13.377415197
6	1.550964800	6.986453679	13.450793796
1	1.996967853	7.665149071	14.172306089
6	14.736916992	4.193078001	14.726180099
1	13.993353662	3.787981565	15.418819617
1	15.538537734	4.653308162	15.316442742
1	15.179274689	3.346013135	14.185294750
6	-0.741438090	7.704740657	14.269104243
1	-1.676394041	7.182726970	14.499417536
1	-1.008411907	8.655046188	13.787767838
1	-0.247158771	7.947306484	15.215644883
8	8.082516807	1.491040834	14.822998196
6	9.128151884	1.831159895	15.653512651
6	9.722427681	3.106214210	15.657059033
1	9.365331810	3.868848674	14.972621652
6	10.765373831	3.378727942	16.553758513
1	11.212270347	4.369310855	16.561380366
6	11.226681364	2.395006810	17.439035119
1	12.036073496	2.611630310	18.129110457
6	10.630824848	1.124140157	17.420294997
1	10.980546378	0.351319484	18.098524951
6	9.588045826	0.840420962	16.536768714
1	9.113367744	-0.133602205	16.509422889
1	7.830838769	2.212924401	14.195784480

^{4}2{B3LYP}$_{optBS2}$

7	5.719626860	6.798962739	11.529694376
7	8.457016087	6.566163386	11.138182020
6	5.779980967	7.109838978	10.184189866
6	4.566976060	7.800354183	9.793190784
6	3.728288243	7.787558991	10.892580231
6	4.433510833	7.083066167	11.948776492
6	3.902189546	6.547529630	13.140756695
6	9.800798967	6.236577803	11.217766179
6	10.184937843	5.467527279	10.049047707
6	9.078701029	5.417065377	9.221165188
6	8.029258372	6.172992685	9.878140975
6	6.803862595	6.612292784	9.353468258
6	11.464808566	4.700199466	9.818365314
1	12.334931644	5.291774893	10.099869527
1	11.571330358	4.499887205	8.746414357
6	11.499355747	3.359646286	10.581170903
1	12.455810470	2.849764652	10.413402145
1	10.696447407	2.693399488	10.248476441
1	11.375651046	3.512826011	11.657439994
6	8.970352372	4.563650800	7.979562263
1	9.977553505	4.344397466	7.608581336
1	8.461523545	5.093254656	7.174389924
6	8.238011026	3.230677447	8.243861830
1	8.157137159	2.650131769	7.316699265
1	7.228235180	3.401801072	8.629078376
1	8.773482361	2.623563205	8.981525368
6	4.302409565	8.533975983	8.501429055
1	3.231382592	8.748840820	8.425417708
1	4.545122195	7.909644438	7.641025342
6	5.081806781	9.859556411	8.397096952
1	4.849230231	10.370540621	7.454823561
1	6.162592764	9.684486977	8.429164880
1	4.828216025	10.534829607	9.222682860
6	2.409739631	8.515775705	10.983669227
1	1.641800338	7.886572326	11.433977757
1	2.058078224	8.741902599	9.971603478
6	2.511737134	9.833956376	11.776208605
1	1.540497863	10.343039684	11.805722735
1	3.239713475	10.514945685	11.319889521
1	2.829025308	9.650346902	12.808511236
6	6.539754366	6.480452024	7.889170106
6	5.605507074	5.533142398	7.446553376
1	5.092932281	4.924317529	8.186113838
6	5.364122393	5.343705316	6.082396138
6	6.070829128	6.131147244	5.163526342
1	5.900510029	5.984315171	4.097350733
6	7.000130411	7.093144900	5.577025560
6	7.230399735	7.254048084	6.948926588
1	7.968753957	7.976072392	7.291277425
6	4.359321139	4.315549450	5.610758856
1	4.711035727	3.791751620	4.714115287
1	3.399901302	4.785502770	5.354168616
1	4.162854638	3.566833584	6.385105995
6	7.738686975	7.939264691	4.563652517
1	8.740743735	8.205619831	4.917748140
1	7.204772786	8.877673226	4.362610283
1	7.845769601	7.415025789	3.607475138
6	2.416571361	6.415970948	13.245562116
6	1.756998414	5.469433584	12.446719380
1	2.340070169	4.856100354	11.764821803
6	0.373578631	5.294434147	12.528168472
6	-0.349450716	6.105613855	13.415374346
1	-1.428977814	5.978307842	13.490140532
6	14.144797983	7.504605937	11.084699031
6	12.752208782	7.535470401	11.215863684
1	12.167472141	8.226781432	10.612713094
6	-0.330373163	4.255455671	11.682862245
1	0.385699344	3.621373637	11.150515933
1	-0.983846791	4.725088994	10.935527695
1	-0.963959114	3.604503901	12.298428042
6	14.854900925	8.450675334	10.141680071
1	15.820810471	8.045154604	9.821247521
1	15.050043736	9.420323686	10.620059163
1	14.256021138	8.646975918	9.245172834
8	10.102611920	3.832831267	14.146699357
6	10.374700802	2.634091121	14.731800951
6	9.559251361	1.505890732	14.564715321
1	8.667619604	1.577125849	13.947955927
6	9.901289307	0.308440443	15.193015106
1	9.261929318	-0.561331455	15.058486020
6	11.047077189	0.216272153	15.985144850
1	11.306904012	-0.720879272	16.470376212
6	11.855492993	1.344612888	16.146152305
1	12.752499283	1.287652978	16.759139994
6	11.525835604	2.549474541	15.527509413
1	12.145942352	3.433590085	15.649457813
1	9.252901007	3.799648017	13.649254979
26	7.262465949	6.390730735	12.697402593
17	7.343816870	3.909618242	12.594590580
7	8.794337795	6.792152302	13.883248298
7	6.063058878	6.415113557	14.262199059
6	8.731425083	7.009474252	15.245285739
6	9.916854615	7.729640318	15.675157768
6	10.739306894	7.839060178	14.569186481
6	10.062068771	7.167522554	13.476136176
6	10.609902101	6.718857403	12.259467274
6	4.724242260	6.060042444	14.171242591
6	4.355463754	5.255712805	15.324531342
6	5.465862207	5.199098877	16.147222871
6	6.505055347	5.987934463	15.507556217
6	7.726469976	6.426370685	16.046240007
6	3.076819415	4.488344802	15.568688617
1	2.206127250	5.071310246	15.274212641
1	2.969901662	4.316037171	16.645648551
6	3.034416647	3.126593259	14.845736147
1	2.082724140	2.619514576	15.047379798
1	3.845534422	2.471317779	15.180185935
1	3.130836800	3.245270557	13.762007087

6	5.571312033	4.333654197	17.381513818
1	4.562015744	4.117958473	17.748631527
1	6.079190315	4.855678572	18.192055502
6	6.293340492	2.995634834	17.119085691
1	6.332599118	2.399607855	18.039288088
1	7.320073688	3.154614065	16.775513383
1	5.776715299	2.407088956	16.353526015
6	10.174456074	8.380251422	17.012502429
1	11.244364421	8.596390506	17.102204810
1	9.937427140	7.700578063	17.831006776
6	9.387438857	9.691981501	17.202019460
1	9.612239745	10.139596986	18.178006613
1	8.307429160	9.514324710	17.151838270
1	9.641710830	10.422954022	16.425474869
6	12.012684485	8.646508897	14.508752623
1	12.814849041	8.082893695	14.031942483
1	12.353403027	8.851798077	15.528899249
6	11.832549115	9.987199692	13.769763016
1	12.771725715	10.553811577	13.766377584
1	11.063717015	10.604317637	14.249173798
1	11.531022579	9.826418913	12.729264980
6	8.017923008	6.216977093	17.496967049
6	8.970845064	5.260813768	17.874623819
1	9.486016993	4.694869749	17.101662867
6	9.252626359	5.017043004	19.223679341
6	8.569699090	5.762845833	20.192497310
1	8.776417213	5.578855973	21.246569813
6	7.623355734	6.735506313	19.844929950
6	7.350795120	6.947771863	18.488729310
1	6.603926082	7.682915106	18.196037274
6	10.277710174	3.979870641	19.625862683
1	9.909481782	3.350765584	20.445250156
1	11.204440818	4.453976509	19.976306647
1	10.536290380	3.326678451	18.786423949
6	6.919794462	7.539830826	20.915098187
1	6.027505261	8.037234126	20.520955005
1	7.577650294	8.317978292	21.323846093
1	6.610772962	6.906229108	21.754817526
6	12.094994944	6.685259466	12.113910757
6	12.843246357	5.783236463	12.880586613
1	12.319782417	5.119978381	13.564188911
6	14.234769540	5.715454016	12.753886939
6	14.867435389	6.587016815	11.858406336
1	15.951297191	6.546668717	11.756170305
6	0.277642103	7.070222130	14.211358879
6	1.669996519	7.207563145	14.124344011
1	2.180993254	7.935131196	14.751818088
6	15.028857777	4.699254540	13.544140057
1	14.625740646	4.574801391	14.555487083
1	16.081442708	4.989323210	13.630854905
1	14.996870913	3.712467491	13.063287606
6	-0.525406442	7.956404875	15.138042571
1	-1.492820147	7.506180034	15.384815222
1	-0.726295277	8.934064215	14.679053095
1	0.009323399	8.145001928	16.076066392
8	5.031101845	3.841699187	10.434441392
6	4.341458064	2.670776229	10.367254476
6	4.692342386	1.538482941	11.115562737
1	5.547257604	1.583580602	11.784871326
6	3.939246303	0.370740351	10.992735712
1	4.217738971	-0.502790144	11.578394249
6	2.841479877	0.312598990	10.132023024
1	2.260904588	-0.601736548	10.041591194
6	2.498729004	1.444772850	9.388067423
1	1.646614792	1.414030358	8.712164216
6	3.238631519	2.620884080	9.502244812
1	2.976198808	3.509565755	8.934254211
1	5.755058402	3.794346869	11.100745960

^{6}2{B3LYP}$_{optBS1}$

7	5.882052255	6.664799118	11.288647352
7	8.714570642	6.399877826	11.265274794
6	6.157767857	6.992664235	9.960518393
6	5.024732491	7.737401329	9.422313828
6	4.034318883	7.742523421	10.398876407
6	4.552783911	7.000898356	11.543180627
6	3.844868638	6.510725824	12.671661409
6	10.034287434	6.053495065	11.563461447
6	10.570943175	5.291259434	10.419236113
6	9.590129097	5.273856229	9.434027027
6	8.453413300	6.037713433	9.945115559
6	7.296525967	6.495995136	9.275986856
6	11.861763148	4.509093498	10.357900117
1	12.687225207	5.077567937	10.787116238
1	12.126630224	4.339535923	9.307621784
6	11.773107388	3.138347702	11.071490896
1	12.728027127	2.605155009	10.991876403
1	10.992811363	2.510678419	10.628917795
1	11.539324836	3.259877029	12.134002274
6	9.653398231	4.450648397	8.169942680
1	10.698905951	4.197965216	7.960213493
1	9.302235977	5.021592704	7.309053086
6	8.838300554	3.137742686	8.265744052
1	8.948750494	2.554273871	7.343997409
1	7.773107031	3.341160066	8.408463139
1	9.170997133	2.518869120	9.105092645
6	4.965651562	8.511631810	8.127829434
1	3.920079529	8.739740307	7.892861704
1	5.336423530	7.912320891	7.295963472
6	5.760547067	9.837576141	8.183232704
1	5.677387979	10.375189958	7.231240675
1	6.822029362	9.650049109	8.375757494
1	5.384826064	10.490114572	8.979109940
6	2.744853140	8.523538934	10.318542596
1	1.901660049	7.924440767	10.664465466
1	2.534663907	8.762921785	9.270146060
6	2.787583826	9.842271233	11.126571923
1	1.839093315	10.383957779	11.027866858
1	3.593444028	10.495706887	10.773396215
1	2.957554944	9.645399603	12.190539661
6	7.249251762	6.425483094	7.779288417
6	6.355618434	5.540796417	7.149303964
1	5.713549754	4.911167051	7.756580448
6	6.298531007	5.442277659	5.750773303
6	7.144867507	6.263292139	4.985123059
1	7.110107489	6.195943586	3.899914738
6	8.041602295	7.162549909	5.586570190
6	8.091121488	7.226606827	6.989134160
1	8.788955013	7.903873906	7.473210218
6	5.344763000	4.474812173	5.080610289
1	5.866729171	3.841185628	4.353637004
1	4.555245865	5.007934449	4.535150106
1	4.861682831	3.819882623	5.811531755
6	8.921934718	8.055175481	4.736737101
1	9.786004476	8.419871836	5.301542373
1	8.368744552	8.934137757	4.377977804
1	9.294664325	7.524561181	3.853108147
6	2.351151735	6.430311735	12.578763265
6	1.761729233	5.520706073	11.681194276
1	2.398531400	4.883253275	11.077072167
6	0.368262752	5.404549840	11.579061136
6	-0.434342002	6.232030962	12.385245269
1	-1.516929654	6.151190321	12.316141704
6	14.377750083	7.200311780	12.060128131
6	12.975770441	7.267243016	11.986526726
1	12.505092152	7.957723242	11.292595775
6	-0.260949853	4.402505351	10.633491540
1	0.499820582	3.846058053	10.077771716
1	-0.917406236	4.896767932	9.905960840
1	-0.873962717	3.673522810	11.178249815
6	15.240067509	8.108203654	11.208088891
1	16.201359072	7.639302586	10.971772351
1	15.455838306	9.053247154	11.724788784
1	14.744427191	8.359636005	10.264297250
8	6.343509548	1.433503550	10.652826674
6	5.275064623	1.766794277	9.847363294
6	4.711526296	3.054495989	9.811824201
1	5.109063428	3.837099891	10.449946559
6	3.641973894	3.315611606	8.943389808
1	3.220814036	4.316682764	8.907470451
6	3.124786261	2.307540740	8.118647597
1	2.295351520	2.515616247	7.450085309
6	3.690889422	1.023799924	8.169567450
1	3.297425901	0.232285204	7.538554610
6	4.759460680	0.751466742	9.025259765
1	5.211977195	-0.232046068	9.077226107
1	6.631826503	2.170046371	11.243224592
26	7.260774792	6.107134122	12.696348490
17	7.268386316	3.668962819	12.698234426
7	8.640347847	6.665000654	14.102334517
7	5.805261113	6.397445490	14.125399175
6	8.365173197	6.988406882	15.430957442
6	9.499927096	7.728475523	15.973006273
6	10.490244845	7.736256456	14.996469405
6	9.970674062	7.000045789	13.848569833
6	10.675747725	6.514933326	12.717460802
6	4.484390632	6.052944113	13.844299699
6	3.945933288	5.295317122	14.973988245
6	4.925375883	5.280194939	15.960381701
6	6.065412808	6.038484529	15.446369014
6	7.224738371	6.492944101	16.113896204
6	2.653911710	4.515283830	15.037118592
1	1.829713013	5.082902570	14.604628240
1	2.387463076	4.350081797	16.087662867
6	2.741548927	3.141676570	14.328876857
1	1.785539882	2.610572000	14.408952272
1	3.519946328	2.514452709	14.775216699
1	2.976966492	3.258973785	13.266341426
6	4.854992943	4.465121714	17.229615806
1	3.807078912	4.223083418	17.440203966
1	5.210150732	5.038622442	18.086958723
6	5.658328666	3.144382698	17.143724370
1	5.540728217	2.568131574	18.069179437
1	6.725559922	3.336939641	17.002022214
1	5.322092489	2.522742107	16.307948840
6	9.562537662	8.494878749	17.272182522
1	10.608902392	8.722626379	17.504657765
1	9.195691145	7.889707607	18.101745050
6	8.767032878	9.820929279	17.229087609
1	8.857215803	10.353069503	18.183646326
1	7.703890813	9.634522766	17.042873958
1	9.137425042	10.478494867	16.434395666
6	11.781257830	8.514359606	15.082124661
1	12.621637783	7.920691030	14.721037490
1	11.998240832	8.735446962	16.133129223
6	11.734998596	9.846785117	14.297070703
1	12.685054039	10.385153387	14.398217038
1	10.932940368	10.495380882	14.667107357
1	11.557144540	9.668239770	13.231215050
6	7.274061714	6.419048099	17.609752686
6	8.161818340	5.526672627	18.237237205
1	8.799744810	4.894604178	17.628249127
6	8.217947269	5.423960694	19.635503134
6	7.376548921	6.248082175	20.402766385
1	7.410337031	6.177216956	21.487916808
6	6.486226906	7.155186457	19.804184113
6	6.437560552	7.223564894	18.401974829
1	5.743727308	7.906054230	17.919604643
6	9.164216299	4.448078810	20.304172944
1	8.634483547	3.807551950	21.019978210
1	9.949688537	4.974326310	20.862630358
1	9.652791256	3.799927214	19.570767360
6	5.611406726	8.050276904	20.656798142
1	4.751443502	8.424409389	20.092110584
1	6.170575739	8.922975823	21.021159480
1	5.232865251	7.518074565	21.536965659
6	12.170326860	6.449436306	12.795063761
6	12.783170392	5.543971115	13.680118801
1	12.162521317	4.897097809	14.291458571
6	14.179157275	5.444753038	13.763891291
6	14.961135684	6.284460592	12.950429042
1	16.045442026	6.217585344	13.006144568
6	0.125716075	7.152835062	13.285548693
6	1.525669472	7.236618749	13.378290696
1	1.978663038	7.930610752	14.080485777
6	14.832122225	4.444963946	14.695024375
1	14.087428355	3.917206628	15.298684057
1	15.537044025	4.935424598	15.378223840
1	15.398324140	3.690816432	14.133270955
6	-0.759054612	8.046500425	14.129713476
1	-1.717671975	7.565066166	14.351108232
1	-0.979352116	8.991146327	13.614580361
1	-0.279385957	8.299555344	15.081147171
8	8.126065262	1.464692163	14.805732050
6	9.212150601	1.785473555	15.592900594
6	9.813664813	3.056470961	15.590809713
1	9.432833812	3.834264446	14.936776868
6	10.898760366	3.307335699	16.442692762
1	11.348921264	4.296320940	16.450436189
6	11.393681270	2.305298652	17.288270319
1	12.235340202	2.505144790	17.943939989
6	10.789399434	1.038152774	17.275145431
1	11.165227905	0.251460044	17.922787394
6	9.705031314	0.776292817	16.436116351
1	9.223023243	-0.194232075	16.413503740
1	7.857240843	2.193277155	14.196438236

^{6}2{B3LYP}$_{optBS2}$

7	5.881481410	6.623246260	11.275933964
7	8.748255310	6.470755004	11.232751321
6	6.124836304	7.002845151	9.969390431
6	4.928799698	7.635380971	9.427798664
6	3.940836371	7.510900666	10.387478544
6	4.542184071	6.830830535	11.527180525
6	3.891684735	6.336694407	12.686976115
6	10.050783578	6.080653018	11.485373390
6	10.594987611	5.417554771	10.305610260
6	9.635208840	5.515711271	9.316578446
6	8.492857970	6.208482413	9.906319613
6	7.305668334	6.647993147	9.275136128
6	11.871306301	4.618945482	10.180792524
1	12.718059412	5.151004839	10.613951155
1	12.106464245	4.491829913	9.118721079
6	11.772834477	3.223425972	10.830548735
1	12.713406802	2.675564783	10.696809800
1	10.966633623	2.631305464	10.385322998
1	11.574605123	3.297333390	11.904052059
6	9.747996198	4.868475086	7.955608631
1	10.808962071	4.771419298	7.699088107
1	9.312399094	5.498276596	7.181030581
6	9.096845066	3.471494285	7.893840329
1	9.196276962	3.048989760	6.886383639
1	8.030494093	3.516034995	8.137491827
1	9.567582074	2.779122882	8.599676220
6	4.791272208	8.443476034	8.158268308
1	3.727714221	8.571741513	7.931925941
1	5.217403238	7.920692619	7.302263474
6	5.439832207	9.838173364	8.268791893
1	5.303217930	10.399545899	7.336331943
1	6.515020004	9.760173539	8.461749782
1	4.996151617	10.420433510	9.084485397
6	2.566870029	8.129790380	10.283303818
1	1.801021399	7.452474642	10.658497639
1	2.330051268	8.290318817	9.226145717
6	2.454424694	9.477868389	11.022355464
1	1.443743905	9.890963403	10.916525202
1	3.164426221	10.211081471	10.623248453
1	2.661720027	9.361598687	12.091396000
6	7.277450263	6.691859103	7.780710467
6	6.515160691	5.754303433	7.068287415
1	5.949240017	5.003764746	7.614761209
6	6.487640101	5.761150089	5.671141777
6	7.223672873	6.744311190	4.993382071
1	7.206123591	6.762149553	3.904104732
6	7.983912270	7.699560831	5.677077988
6	8.011476805	7.653286886	7.077216685
1	8.612433613	8.373503013	7.628652703
6	5.698145359	4.722218387	4.906348073
1	6.365599386	4.027985112	4.379186770
1	5.053549982	5.185780129	4.149312548
1	5.066849053	4.129034896	5.575348010
6	8.745067054	8.770679548	4.927242846
1	9.679562429	9.029849261	5.437592747
1	8.155293239	9.693907577	4.844188510
1	8.992609127	8.449308613	3.909758771
6	2.409011566	6.152337818	12.626318967
6	1.873525552	5.142875129	11.812615317
1	2.546251466	4.508516039	11.240677497
6	0.494307024	4.932050990	11.737928719
6	-0.349318715	5.770476834	12.480855236
1	-1.427164456	5.620963202	12.426142285
6	14.467823487	6.890934932	12.233158146
6	13.084220846	7.061293559	12.089335722
1	12.701493478	7.830609338	11.421953391
6	-0.074701110	3.816931372	10.889351664
1	0.687625554	3.385746355	10.232985641

1	-0.903047111	4.172086459	10.264133657
1	-0.468912300	3.005800694	11.515582558
6	15.432796732	7.795333841	11.498909889
1	16.425415865	7.340217771	11.417036032
1	15.553237478	8.754533282	12.020507049
1	15.079394373	8.021525904	10.486401045
8	6.274637337	1.342962784	10.822529901
6	5.313897180	1.719889996	9.945136182
6	4.879957114	3.049176176	9.814530437
1	5.320970952	3.821803066	10.438282851
6	3.887342446	3.366014693	8.883870608
1	3.562027739	4.400494882	8.791528942
6	3.317385383	2.375127792	8.080261923
1	2.544401362	2.626536321	7.358434347
6	3.755146593	1.053902249	8.219048557
1	3.320821077	0.270707678	7.601519449
6	4.744061756	0.722614063	9.140871794
1	5.091730247	-0.299902677	9.257291486
1	6.587470616	2.119707960	11.332030822
26	7.301383229	6.099265636	12.676550598
17	7.332735003	3.708376060	12.631926049
7	8.699757416	6.680763695	14.079384187
7	5.852252947	6.426082228	14.127921690
6	8.441068164	7.076467921	15.377295496
6	9.604697428	7.774305239	15.903595210
6	10.600192691	7.664940940	14.950380515
6	10.028915136	6.934717307	13.828406181
6	10.703577291	6.439071793	12.681255530
6	4.564542449	5.994505565	13.879002440
6	4.044645672	5.312680262	15.059335299
6	4.999452355	5.449192357	16.049318363
6	6.115332157	6.180199564	15.457897100
6	7.282544314	6.672859105	16.082404624
6	2.806014562	4.456255318	15.179890803
1	1.935010764	4.950031813	14.750592571
1	2.577612465	4.311140957	16.241063967
6	2.971665683	3.071686958	14.520740433
1	2.058605516	2.478433963	14.650650097
1	3.805087816	2.515665932	14.962417197
1	3.165318208	3.162113611	13.447492160
6	4.906466978	4.807871590	17.414422776
1	3.848784714	4.665806680	17.663010441
1	5.306442631	5.464522773	18.186431834
6	5.619756905	3.443454051	17.496428807
1	5.490481235	3.007894486	18.494604651
1	6.693246801	3.542091942	17.309403951
1	5.222987441	2.734793215	16.762321415
6	9.699169185	8.622966126	17.149544218
1	10.753645348	8.822716778	17.365836110
1	9.310313214	8.097583150	18.021104668
6	8.964995662	9.971483781	17.005802779
1	9.072461837	10.566902002	17.920863622
1	7.895456836	9.822320274	16.823485053
1	9.366955759	10.556059309	16.170251763
6	11.948756350	8.338456311	15.041599133
1	12.742655228	7.678207929	14.694867924
1	12.171462192	8.543828141	16.093941115
6	12.014995142	9.663920703	14.257852784
1	13.007917515	10.120023095	14.355155808
1	11.273873965	10.381456598	14.628022615
1	11.818925991	9.504163407	13.192168298
6	7.333609616	6.711986550	17.575581066
6	8.162980559	5.814281357	18.264280768
1	8.754399211	5.097718585	17.699366226
6	8.224724750	5.814972509	19.659867076
6	7.450318894	6.751313752	20.361049127
1	7.490895340	6.763197715	21.449807153
6	6.623102128	7.666983078	19.701291517
6	6.565132659	7.627713293	18.301737284
1	5.913894459	8.317896633	17.769365606
6	9.098204326	4.825871592	20.399665775
1	8.500787977	4.189378024	21.064727947
1	9.840359678	5.337825257	21.025799625
1	9.634323150	4.170162592	19.706423567
6	5.820247839	8.689370006	20.475481510
1	4.857525811	8.892073652	19.992729799
1	6.355142172	9.646570658	20.544284084
1	5.621660257	8.352409179	21.498597475
6	12.183472736	6.258192825	12.796358359
6	12.681118075	5.262395143	13.650848834
1	11.980126514	4.634080652	14.195343931
6	14.054587503	5.055356766	13.799808828
6	14.932704543	5.882819927	13.084795560
1	16.006675320	5.736341905	13.194640250
6	0.154786242	6.793177147	13.292095411
6	1.543408507	6.966143254	13.364828205
1	1.957668616	7.743939799	14.002967774
6	14.584553056	3.965461783	14.705452537
1	13.771783384	3.434447009	15.210979293
1	15.250996305	4.374886269	15.475465357
1	15.164255155	3.225154090	14.139498284
6	-0.774508110	7.708330474	14.058457309
1	-1.762466517	7.255303545	14.192474014
1	-0.918691042	8.660876397	13.530684839
1	-0.374173760	7.947108100	15.050439346
8	7.614625716	1.420241834	14.786517297
6	8.703857000	1.596304160	15.575821947
6	9.453099009	2.784225094	15.587715681
1	9.171154177	3.603142325	14.931986147
6	10.552386054	2.901420532	16.440876856
1	11.120411028	3.830069803	16.447500848
6	10.921247030	1.849160803	17.282810196
1	11.779125083	1.944758057	17.943293144
6	10.171630631	0.668944681	17.261185265
1	10.447166962	-0.160592629	17.908604440
6	9.071575957	0.537497807	16.417855794
1	8.482263094	-0.374456822	16.392381942
1	7.501062695	2.195322657	14.197604174

^{2}4{B3LYP}$_{optBS1}$

26	5.529621095	10.276694368	17.871577342
17	7.174006686	8.769927441	18.758416403
7	5.726600865	11.541304033	19.349682107
7	3.879140144	9.589232548	18.711100653
7	5.208391408	9.080213737	16.343905785
7	6.575629044	11.476361040	16.718546650
6	6.844064132	12.367771820	19.483001833
6	7.236182316	12.419117497	20.885313760
6	6.281094096	11.706280957	21.600554237
6	5.291926905	11.238756542	20.641452769
6	4.002993867	10.725773397	20.874041451
6	3.260209191	10.110744868	19.848247889
6	1.855571406	9.715128910	19.848783493
6	1.684847465	8.828945934	18.794977571
6	2.981723603	8.688915254	18.142613249
6	3.407861339	7.694552477	17.235082928
6	4.595383699	7.832412405	16.498777652
6	5.301936947	6.847979068	15.688819839
6	6.260334941	7.536167579	14.957109809
6	6.147767685	8.944746854	15.320844694
6	6.742924629	10.066271607	14.714817237
6	6.755363250	11.331459430	15.341246139
6	7.129783973	12.622950470	14.774759472
6	7.301344142	13.500897364	15.836265570
6	7.033509625	12.753469993	17.060316977
6	7.340324424	13.104708670	18.390946766
6	8.502224029	12.991453647	21.477233051
1	8.742406939	13.954894208	21.028413103
1	8.339862104	13.188722027	22.543664628
6	6.381289891	11.348471884	23.064350516
1	7.097962358	12.021358735	23.548598472
1	5.429657532	11.500725985	23.577080114
6	6.845670651	9.889317412	23.293421398
1	6.929687101	9.679697216	24.366772701
1	6.137586561	9.175686602	22.860363770
1	7.820999108	9.707162493	22.830534215
6	0.745195241	10.278467057	20.703222957
1	-0.130995407	9.624319162	20.629126864
1	1.024402175	10.289995188	21.758180446
6	0.330474140	11.708170987	20.281547558
1	-0.491712421	12.071262710	20.909996316
1	1.169127249	12.405272404	20.382965367
1	-0.001037860	11.730613689	19.237148623
6	0.362344837	8.266610471	18.330754944
1	0.441465342	7.199327538	18.115523424
1	-0.369658246	8.356963988	19.141414069
6	-0.194338743	8.987793069	17.080398844
1	-1.161799514	8.561485817	16.788302948
1	-0.335708222	10.057549282	17.272619360
1	0.491715132	8.888034041	16.232116291
6	5.168092912	5.343895859	15.732006088
1	4.120561493	5.039696004	15.729066414
1	5.607203263	4.921091568	14.821055550
6	5.869779257	4.708297025	16.956334100
1	5.762188182	3.617077120	16.933267818
1	6.937452929	4.947868941	16.972443736
1	5.439971217	5.074002321	17.893949938
6	7.313353282	6.886162365	14.091397107
1	6.952888295	5.902887229	13.767273984
1	7.482165557	7.459045905	13.179491464
6	8.665654174	6.700067556	14.822339772
1	9.394666454	6.221489667	14.157148173
1	9.077576832	7.661655793	15.144081174
1	8.551732777	6.074086447	15.713405238
6	7.172144943	13.009577928	13.331513333
1	7.725695882	13.949505728	13.210674563
1	7.719161976	12.271116126	12.727681652
6	5.765100584	13.195990915	12.699977712
1	5.842940983	13.491284153	11.646667152
1	5.188439056	12.266190994	12.750594716
1	5.201009557	13.970567197	13.231813796
6	7.565156562	14.981417520	15.699702052
1	8.314275874	15.315760322	16.418323803
1	7.988889233	15.179052357	14.708609309
6	6.289893723	15.839690177	15.870256793
1	6.524253298	16.903555251	15.745593550
1	5.528779374	15.567705985	15.130282838
1	5.855709424	15.702541412	16.866058363
6	3.410841341	10.827935755	22.244875556
6	3.081631158	12.083616177	22.787991448
1	3.265071754	12.978916220	22.202119145
6	2.526631178	12.194299218	24.069962621
6	2.640104002	9.754069086	24.302802915
6	3.181947520	9.672594008	23.008267791
1	3.434461646	8.702594833	22.590252256
6	2.591392543	6.442767521	17.136469903
6	2.515242951	5.585696140	18.251753918
1	3.052860665	5.851571930	19.156955549
6	1.761100817	4.406788455	18.211620596
6	1.061162841	4.099639165	17.029509116
1	0.465365476	3.190801801	16.986925348
6	1.112479188	4.935667195	15.904405596
6	1.889006539	6.107380739	15.969777276
1	1.944815190	6.766159297	15.107619638
6	7.420625466	9.903782686	13.386896528
6	6.675383433	9.656083334	12.222503265
1	5.593454838	9.581534874	12.289441717
6	7.310450390	9.498060115	10.978378808
6	8.711248827	9.579551664	10.924763377
1	9.212913289	9.448560331	9.968952655
6	9.482991468	9.819163331	12.075698514
6	8.822574934	9.985184246	13.302287551
1	9.401953749	10.161339074	14.203213602
6	8.213885083	14.290761036	18.667125597
6	9.585474066	14.256316216	18.375618238
1	10.016037030	13.361733507	17.938492715
6	9.829973705	16.508980260	19.213358751
6	8.458299204	16.570290954	19.515528006
6	7.662552544	15.448650723	19.243197655
1	6.603425655	15.469190137	19.479974667
1	2.061838451	7.674762672	24.520882832
6	1.692993644	3.480445446	19.408061232
1	2.101433863	2.490054837	19.169351211
1	2.260648245	3.879355987	20.254396516
1	0.657617663	3.331330370	19.739846627
6	0.357759652	4.586507781	14.638614832
1	-0.307141873	3.731096125	14.793563127
1	-0.252673534	5.429116933	14.291833980
1	1.045827129	4.328992944	13.822655146
6	6.497049054	9.234676331	9.727681111
1	7.136715083	9.174848454	8.841545618
1	5.941843943	8.290767711	9.802855838
1	5.759974075	10.028943168	9.555492739
6	10.992552824	9.911683235	11.995541457
1	11.320721642	10.951359137	11.859870391
1	11.463272834	9.545416116	12.913543859
1	11.384677158	9.331813861	11.153060788
6	7.862807892	17.819088645	20.129858684
1	8.080210280	18.706314050	19.523337387
1	6.776203445	17.736289153	20.224892531
1	8.271884760	18.005794768	21.130468139
7	10.301981655	9.170213316	18.594489348
1	9.283047388	9.172154543	18.558198434
6	11.243549925	11.587063226	15.952867805
1	10.748360322	12.252043619	15.249518338
6	10.461265426	10.805047105	16.808414549
1	9.377743622	10.863117780	16.771628240
7	7.078431821	5.881932113	20.502066099
1	7.111246728	6.733890083	19.956074921
1	6.182722962	5.446254264	20.636077561
6	8.142127274	4.116944682	21.790977410
1	7.172149580	3.657881790	21.966881683
6	8.217548241	5.301595495	21.021598159
6	9.722142685	12.050844372	21.327310269
1	10.617182904	12.517331795	21.756909182
1	9.552549742	11.098349904	21.840877342
1	9.925282881	11.826099813	20.275768432
6	10.410173226	15.360593784	18.654281484
1	10.458108260	17.372435070	19.422503115
6	11.897731347	15.292671905	18.383866589
1	12.120841305	14.622929062	17.548146295
1	12.309537714	16.281634244	18.155784890
1	12.438768709	14.906643758	19.257144950
1	10.731637278	8.550806136	19.261066849
6	11.070676299	9.929370219	17.742208315
6	12.485785384	9.871714545	17.775446629
6	13.252561019	10.659159373	16.915157584
1	14.336479902	10.593873343	16.964104883
6	12.644896016	11.526449917	15.994404103
1	13.246430403	12.134361967	15.325987479
6	9.294201308	3.539865850	22.325215098
6	10.552430415	4.118517890	22.112674200
1	11.445611137	3.668320586	22.531021514
6	10.634936390	5.289799843	21.347333726
1	11.602872679	5.750846263	21.167927575
6	9.492125778	5.877293615	20.805172262
6	2.314738511	11.019150945	24.814630722
1	1.890447745	11.093402976	25.813357243
6	2.154002675	13.544648724	24.644629122
1	1.064485584	13.663304918	24.710807716
1	2.554392638	13.671436801	25.657632450
1	2.539380158	14.361262342	24.026419250
6	2.440133069	8.502509926	25.131229560
1	1.731453521	8.671943649	25.948170558
1	3.385824545	8.168442717	25.578564253
1	9.206719372	2.630537605	22.913743659
1	9.573339216	6.780917240	20.208021289
1	12.973069605	9.205283000	18.483082090

^{2}4{B3LYP}$_{optBS2}$

26	5.754148012	10.297996828	17.794522293
17	7.358491692	8.761858989	18.592305067
7	5.961960622	11.607011048	19.258034428
7	4.230971040	9.483611550	18.754642888
7	5.390562253	9.095684994	16.251901235
7	6.643978006	11.569507450	16.579227863
6	6.917438549	12.604922729	19.291619736
6	7.418765711	12.764837286	20.648048825
6	6.718056576	11.872243659	21.436814417
6	5.740852251	11.230422731	20.573697790
6	4.595745871	10.509694273	20.946269797
6	3.775855105	9.843194164	20.005149795
6	2.439031247	9.299356381	20.208048161
6	2.150436000	8.527972573	19.098513069
6	3.326118874	8.571033154	18.241791613
6	3.659143307	7.677979819	17.201403748
6	4.779975395	7.862864633	16.370994317
6	5.433866892	6.900370413	15.494727055
6	6.430194578	7.584768862	14.828660542
6	6.332105859	8.974581539	15.242453261
6	6.918096493	10.100513763	14.647441681
6	6.838710034	11.387593079	15.228186589
6	7.061320160	12.672364400	14.584807636
6	7.094376887	13.619387350	15.589380137
6	6.929448846	12.902402136	16.844364198
6	7.229610682	13.358841306	18.141333132
6	8.547165253	13.634841750	21.152560084

1	8.535891649	14.607617759	20.662705917
1	8.376328669	13.837178133	22.216314407
6	7.078947610	11.522606724	22.860843652
1	7.718474244	12.313107414	23.267055172
1	6.201155437	11.479232203	23.506545836
6	7.838523374	10.182155955	22.949937779
1	8.145280446	9.988546231	23.985149489
1	7.209832255	9.348972355	22.620328687
1	8.734392646	10.191748386	22.319332769
6	1.460042713	9.611245568	21.315164408
1	0.698246620	8.824777882	21.345262262
1	1.948638977	9.589810390	22.288432478
6	0.758302070	10.970655540	21.133677659
1	0.051108651	11.150787778	21.952270018
1	1.484131274	11.790729150	21.127888564
1	0.203007304	11.008799754	20.189482273
6	0.797948364	7.934273613	18.783551772
1	0.879879609	6.896780314	18.456736879
1	0.197726475	7.914443851	19.698999471
6	0.031989552	8.738759418	17.714674607
1	-0.953251055	8.292700662	17.530555757
1	-0.117973279	9.777360967	18.031857148
1	0.577934575	8.756570005	16.765465422
6	5.203921362	5.412196053	15.361325936
1	4.150335544	5.166495091	15.476734982
1	5.467850716	5.109991869	14.340675116
6	6.029217383	4.567886477	16.352887255
1	5.813777660	3.501094019	16.215686361
1	7.104463273	4.717738250	16.207657827
1	5.795857872	4.829026606	17.390040942
6	7.494284129	6.928756261	13.980813746
1	7.153445250	5.925766030	13.703309272
1	7.652188803	7.463781040	13.044325590
6	8.844956039	6.800903163	14.715954507
1	9.575131051	6.285052383	14.080147503
1	9.253631544	7.782323605	14.973846418
1	8.738256530	6.233577232	15.646614649
6	7.084884141	12.979097510	13.106970237
1	7.523999255	13.971217911	12.957652732
1	7.731651475	12.284074631	12.572096897
6	5.683138498	12.961221786	12.466818362
1	5.744510074	13.195896774	11.397029668
1	5.212175706	11.977670118	12.571146617
1	5.022331849	13.697651801	12.938624996
6	7.118126444	15.113648317	15.369827949
1	7.833625950	15.604703137	16.029939742
1	7.460593595	15.317454460	14.350005817
6	5.732755532	15.761729642	15.560286023
1	5.782715414	16.840151247	15.366938720
1	4.993697564	15.327018583	14.877432691
1	5.367053106	15.618339188	16.582767531
6	4.223274349	10.451661957	22.394572232
6	3.728666569	11.588372822	23.049121812
1	3.622278229	12.518523323	22.494573296
6	3.374686818	11.542806293	24.401485718
6	4.046822620	9.191544455	24.472145468
6	4.377307620	9.259456607	23.112820033
1	4.775887786	8.386063862	22.601265114
6	2.849066588	6.424952401	17.076418981
6	2.998635233	5.416263819	18.041367953
1	3.709500160	5.559408002	18.850804166
6	2.265407577	4.229715193	17.968579597
6	1.357230445	4.074419288	16.910323500
1	0.772247302	3.157510374	16.845473663
6	1.185021935	5.060939742	15.934907997
6	1.946774555	6.235495718	16.026313227
1	1.833447299	7.012428266	15.273111577
6	7.702455765	9.966333367	13.382143104
6	7.097859321	9.673662228	12.156288683
1	6.021684505	9.517974685	12.115943810
6	7.859043423	9.583120951	10.981634519
6	9.241121251	9.777105653	11.065329036
1	9.841385532	9.702383133	10.159323899
6	9.877590348	10.063683763	12.283042558
6	9.092492227	10.164513660	13.433507565
1	9.560223951	10.383911542	14.389592092
6	7.984224483	14.643567339	18.283554914
6	9.334500205	14.694918214	17.910458167
1	9.815886693	13.796022158	17.535337099
6	9.427022741	17.020622587	18.510309921
6	8.079616501	17.000782495	18.891057914
6	7.367750801	15.800426282	18.775067770
1	6.321123065	15.763371046	19.070215768
1	4.229951139	7.034638318	24.595826564
6	2.437469900	3.138198333	19.002180372
1	2.689585963	2.180207213	18.529449627
1	3.232085379	3.380280097	19.714809284
1	1.511352187	2.980191959	19.570879969
6	0.215777767	4.863107987	14.790840333
1	-0.474065896	4.036249775	14.989065825
1	-0.380889951	5.765004398	14.610889678
1	0.744865080	4.634940840	13.856056142
6	7.191070479	9.282472768	9.657596209
1	7.923781433	9.207107025	8.847498774
1	6.635206140	8.337039713	9.694944373
1	6.472224470	10.066106257	9.385324865
6	11.376605970	10.259358855	12.341030035
1	11.693483258	11.104059530	11.715508734
1	11.715889598	10.451075173	13.363772126
1	11.907442086	9.371777408	11.974085270
6	7.418475241	18.244776916	19.441623978
1	7.698195936	19.135788496	18.867783696
1	6.327206897	18.160008497	19.423380416
1	7.717551531	18.425038065	20.482929460
7	10.239614943	9.471294791	19.700299924
1	9.263311738	9.288267165	19.455138800
6	11.173530817	11.341392941	16.642206822
1	10.679655568	11.908294639	15.854315185
6	10.395244056	10.742217767	17.631353728
1	9.312750432	10.828748947	17.606364302
7	5.357402842	7.036163824	20.695737332
1	5.963554188	7.490405991	20.014040071
1	4.374116701	7.182715767	20.504020622
6	4.799569956	4.943488863	21.802393648
1	3.781390313	5.297973439	21.956936971
6	5.719685917	5.765741765	21.122911325
6	9.943484529	13.004869629	20.987077941
1	10.709064426	13.673114463	21.400475075
1	10.015725026	12.040264488	21.498471013
1	10.179879560	12.829488930	19.933534001
6	10.072845200	15.876963507	18.023102073
1	9.988403985	17.950468878	18.598065589
6	11.531656243	15.920167294	17.622941007
1	11.890893286	14.931510666	17.319000955
1	11.695603571	16.611061674	16.785174076
1	12.162612718	16.265211758	18.452440196
1	10.668616556	8.688982971	20.179910678
6	11.003693788	10.010585274	18.672095718
6	12.406783485	9.891598481	18.668554002
6	13.172162063	10.490569028	17.671916306
1	14.254706814	10.381206970	17.696446053
6	12.567647568	11.225774685	16.649752995
1	13.168715132	11.696489535	15.875740114
6	5.187420425	3.694438160	22.284522698
6	6.492548335	3.232321874	22.104371624
1	6.791000767	2.257599737	22.480437944
6	7.407594126	4.044959805	21.430393827
1	8.428285920	3.700852906	21.276374430
6	7.033844202	5.293716970	20.943376348
6	3.542131820	10.337393013	25.096732468
1	3.276512244	10.293758084	26.152728469
6	2.808593174	12.758774629	25.100651405
1	1.719896818	12.678190351	25.222030397
1	3.235976385	12.878993275	26.103166374
1	3.009683790	13.675223786	24.536142505
6	4.260629935	7.911946197	25.250407461
1	3.503715177	7.784797290	26.032843720
1	5.241777280	7.910879114	25.743572792
1	4.459362938	3.078744060	22.808613312
1	7.752508602	5.918133198	20.417869027
1	12.893537461	9.327844667	19.462889588

^{4}4{B3LYP}$_{optBS1}$

26	5.532763739	10.203700677	17.922876271
17	7.323085749	8.749069527	18.810953863
7	5.714689799	11.490478081	19.379220315
7	3.852426017	9.555802810	18.746802559
7	5.203868934	9.021857087	16.395648133
7	6.597398646	11.397368374	16.759223704
6	6.835158609	12.314136835	19.515199423
6	7.197529673	12.391341688	20.924907258
6	6.223974111	11.701401695	21.636622477
6	5.248059960	11.221501990	20.670121356
6	3.948409945	10.734089091	20.890186048
6	3.216516368	10.118071751	19.855843882
6	1.804654285	9.764208511	19.824144951
6	1.633278865	8.872850658	18.770487654
6	2.937204674	8.686729080	18.152858686
6	3.346778893	7.681530586	17.251669738
6	4.556839350	7.788798961	16.546012127
6	5.257172423	6.787934018	15.752868239
6	6.237970740	7.450267333	15.027566577
6	6.150874353	8.863158952	15.378288961
6	6.759906411	9.972793425	14.764133998
6	6.777493442	11.240882957	15.383550043
6	7.163642740	12.522369335	14.809314144
6	7.335329731	13.409656339	15.865664790
6	7.053299918	12.676436574	17.093821492
6	7.348046088	13.039855635	18.424021725
6	8.457357535	12.961137354	21.530745644
1	8.723105098	13.907637685	21.061886507
1	8.274008023	13.189488539	22.587553389
6	6.294772522	11.379967480	23.110644409
1	7.022124856	12.047424830	23.586235134
1	5.340848403	11.570201380	23.606215436
6	6.718874400	9.916640604	23.384881604
1	6.801113810	9.739716532	24.464173665
1	5.987309159	9.211225707	22.978292294
1	7.686148908	9.691639904	22.924151453
6	0.692640602	10.364942923	20.650932599
1	-0.199042068	9.732949863	20.568528625
1	0.954217897	10.381447798	21.710548387
6	0.323407135	11.800695695	20.207389150
1	-0.500194547	12.191527190	20.817147134
1	1.178506910	12.476255183	20.317455544
1	0.011362062	11.821297337	19.156916367
6	0.308022890	8.339738560	18.280005267
1	0.369052408	7.270369873	18.066722011
1	-0.438499837	8.446539014	19.075126835
6	-0.206227216	9.071105105	17.017282130
1	-1.175710813	8.664299041	16.704959913
1	-0.329585637	10.143854918	17.205263946
1	0.495910454	8.955384677	16.184373024
6	5.098214244	5.287455662	15.808172616
1	4.050083867	4.996185638	15.717602883
1	5.612667735	4.843652121	14.948333333
6	5.681552293	4.665987651	17.100158994
1	5.592966005	3.573219682	17.069870575
1	6.737028878	4.924061581	17.229296262
1	5.143057911	5.025884889	17.982569786
6	7.284910806	6.765574672	14.182439582
1	6.896385841	5.796615429	13.846567869
1	7.493471689	7.334802468	13.277397408
6	8.610898898	6.529254040	14.946198949
1	9.340678466	6.031647767	14.296118573
1	9.044684241	7.474885550	15.286466210
1	8.453365367	5.901452894	15.829508987
6	7.222924794	12.890529351	13.345687269
1	7.783910482	13.825149934	13.233942537
1	7.770694569	12.139723072	12.773400557
6	5.823236918	13.076983811	12.713522882
1	5.913602500	13.357966515	11.657329053
1	5.240945495	12.151061678	12.770427759
1	5.258274207	13.861791632	13.229404422
6	7.615225253	14.886330693	15.719212506
1	8.356483002	15.219943651	16.446489123
1	8.055003761	15.072042617	14.732910257
6	6.345002649	15.757012738	15.863977286
1	6.590258180	16.817561156	15.733129262
1	5.592147382	15.484756027	15.115744527
1	5.896151342	15.632367361	16.854800723
6	3.330563132	10.863820149	22.246940150
6	3.015016353	12.131854833	22.768950904
1	3.230376038	13.015994846	22.176123587
6	2.432422407	12.267420584	24.036421959
6	2.485957207	9.827760206	24.295151837
6	3.058153331	9.721538658	23.015488396
1	3.298817252	8.742303222	22.612179795
6	2.483262552	6.464702149	17.113417633
6	2.329112719	5.602021895	18.216648075
1	2.846029600	5.835387815	19.142667844
6	1.524396414	4.458583142	18.138274654
6	0.852977337	4.193320447	16.929603803
1	0.218664080	3.312746898	16.857294484
6	0.980648193	5.036470751	15.816018390
6	1.807009265	6.171136603	15.920001509
1	1.920690250	6.835144865	15.067662533
6	7.428267748	9.810173976	13.431868161
6	6.668867276	9.564473629	12.275692571
1	5.587597180	9.491803884	12.356037523
6	7.287393275	9.408462858	11.023523927
6	8.687366357	9.491776284	10.952666135
1	9.177161426	9.362890295	9.990185068
6	9.473250117	9.732219256	12.093918247
6	8.828698161	9.893639383	13.330259514
1	9.419015270	10.073249939	14.223736878
6	8.217572826	14.228888509	18.702169448
6	9.593480211	14.196530881	18.428080160
1	10.032594504	13.301049343	17.999790479
6	9.816652205	16.458226579	19.249319907
6	8.439345984	16.518894684	19.528989853
6	7.651955105	15.391770369	19.256172329
1	6.587636964	15.412958837	19.472286112
1	1.850350189	7.766004287	24.516146421
6	1.372771216	3.525776943	19.321850231
1	1.738096626	2.518321531	19.084851306
1	1.933149312	3.887685373	20.189357122
1	0.321387029	3.425508836	19.619995521
6	0.251983014	4.735497692	14.522657495
1	-0.444429829	3.899612741	14.642061127
1	-0.321208936	5.603833926	14.175514486
1	0.954292548	4.471089231	13.721298091
6	6.457798474	9.145876374	9.782956714
1	7.085812515	9.087343792	8.888179682
1	5.904556882	8.201462593	9.864522345
1	5.717625693	9.939925097	9.621348987
6	10.980846522	9.832168644	11.993813601
1	11.305299284	10.878927283	11.920734600
1	11.467794436	9.408543691	12.878257428
1	11.358582077	9.307931539	11.109750725
6	7.828889204	17.773187091	20.119474523
1	8.053278714	18.654364763	19.505413421
1	6.740506545	17.688030025	20.197554391
1	8.220683824	17.972665116	21.125409298
7	10.428284745	9.303741469	18.606653212
1	9.411250487	9.226539269	18.562927272
6	11.234527233	11.761186413	15.955223862
1	10.705107729	12.363200736	15.221242437
6	10.497580808	10.918690484	16.794292092
1	9.415335220	10.871764116	16.716886447
7	7.571536704	5.694747899	20.221690551
1	7.530689678	6.593371915	19.756279706
1	6.713892422	5.176580300	20.307454451
6	8.760005823	3.959419098	21.443075622
1	7.829428563	3.407073922	21.562974717
6	8.746773770	5.190010711	20.744649366
6	9.663172406	11.995888924	21.433722510
1	10.557726727	12.461143094	21.865195412
1	9.466999763	11.063981882	21.974882658
1	9.880897590	11.732965588	20.394105383
6	10.409978418	15.306698390	18.708426967
1	10.438517555	17.326033142	19.459253931
6	11.902238334	15.240804542	18.459033661
1	12.138364851	14.569640443	17.626932954
1	12.315350279	16.230610913	18.235372897
1	12.431509977	14.856180678	19.341733098
1	10.896678495	8.727497157	19.286475590
6	11.150798413	10.120767786	17.767422845
6	12.562989957	10.201531945	17.853754524
6	13.284371008	11.048958170	17.011457258
1	14.367093796	11.089415231	17.103466669
6	12.632890491	11.842246461	16.054169959
1	13.199741304	12.494703900	15.397487625
6	9.947375700	3.455264979	21.978945985
6	11.154800991	4.153599860	21.837077971
1	12.076094875	3.759411461	22.256518293
6	11.149974608	5.370341072	21.140173985

1	12.078207953	5.923724904	21.014103004
6	9.970873453	5.887608845	20.597888748
6	2.176794342	11.104677491	24.786931947
1	1.729704225	11.198124955	25.774072049
6	2.076130887	13.630723844	24.590415760
1	0.988222524	13.768113110	24.643499945
1	2.467651226	13.763276666	25.606234169
1	2.481848608	14.433135642	23.966809755
6	2.235825065	8.588764028	25.128626592
1	1.513812590	8.782696517	25.928247363
1	3.162436824	8.234409273	25.599434979
1	9.927391378	2.508650218	22.512910177
1	9.980882220	6.828354090	20.054701338
1	13.084609938	9.595543960	18.590281226

^{4}4{B3LYP}$_{optBS2}$

26	5.829931163	10.256090384	17.813456927
17	7.512696042	8.740745188	18.722546565
7	6.004034824	11.563837198	19.277752601
7	4.259365192	9.459343835	18.741145924
7	5.468059026	9.098745011	16.261340345
7	6.777427654	11.535447779	16.604253755
6	7.005651773	12.519264590	19.333654838
6	7.493008668	12.639597389	20.697268115
6	6.730225904	11.784618029	21.471574362
6	5.742429599	11.186308673	20.588873258
6	4.575200976	10.488987148	20.937435075
6	3.769705263	9.837032183	19.976618496
6	2.426828123	9.306630905	20.143297595
6	2.157427418	8.538239803	19.023215444
6	3.350917603	8.569308504	18.195001304
6	3.705968064	7.674437906	17.160874174
6	4.856902480	7.859139764	16.377119302
6	5.540570404	6.897371279	15.527491635
6	6.533964941	7.588169714	14.860340114
6	6.415416328	8.978434759	15.254287291
6	6.984109868	10.104135497	14.638501930
6	6.940784361	11.378105375	15.241409861
6	7.186232382	12.670896579	14.627536358
6	7.254977190	13.595087786	15.654160973
6	7.080334450	12.856196237	16.894894820
6	7.363400097	13.284265817	18.206342806
6	8.682391211	13.420508231	21.206760875
1	8.711431800	14.417287535	20.767443011
1	8.561584180	13.574711154	22.284690942
6	7.032070073	11.441109522	22.911427099
1	7.640941465	12.241204875	23.345783427
1	6.125911730	11.391873263	23.514749998
6	7.801105753	10.110448386	23.048720653
1	8.035112732	9.914164982	24.102318811
1	7.211009642	9.270043454	22.669750918
1	8.740620196	10.133503049	22.486527084
6	1.424915994	9.620336625	21.228875889
1	0.659342103	8.837103340	21.241785495
1	1.892742561	9.595728678	22.212227952
6	0.732238564	10.982864478	21.033555727
1	0.008371175	11.165479306	21.837149479
1	1.461201423	11.800358768	21.043107547
1	0.197049206	11.023652805	20.077953574
6	0.812139605	7.937004240	18.694039766
1	0.908207823	6.898375443	18.376675172
1	0.198966664	7.920140050	19.601043301
6	0.057981546	8.728425203	17.607639184
1	-0.922528470	8.276342814	17.415043086
1	-0.100267487	9.769482121	17.912450974
1	0.616999961	8.737239061	16.665888087
6	5.352008185	5.400989203	15.448948910
1	4.297886627	5.134987029	15.506915066
1	5.693706403	5.055544233	14.466213855
6	6.126721372	4.639084760	16.542812959
1	5.941208955	3.561186118	16.460664899
1	7.205570118	4.806249605	16.454363044
1	5.821764964	4.963868424	17.542389912
6	7.634005958	6.948381694	14.047539843
1	7.329555667	5.930484929	13.781309379
1	7.794568072	7.471804266	13.104059259
6	8.969998245	6.876928867	14.816119947
1	9.732426588	6.378116792	14.205105418
1	9.338721430	7.875128840	15.071220591
1	8.859929559	6.318521142	15.751807732
6	7.200029995	13.006937041	13.155779805
1	7.668902479	13.987255698	13.018758933
1	7.815687000	12.300421773	12.598984089
6	5.789341012	13.042715868	12.535448535
1	5.843424258	13.300332833	11.470601825
1	5.294856452	12.069260167	12.622782618
1	5.154949580	13.785345080	13.033074278
6	7.319427927	15.092226704	15.464561166
1	8.034143021	15.551161236	16.148200301
1	7.686716695	15.306499453	14.455251587
6	5.946588622	15.771690850	15.643787845
1	6.027840903	16.852481213	15.474561767
1	5.208965875	15.371287525	14.938476175
1	5.558832670	15.616792834	16.656572741
6	4.173679554	10.422512153	22.377601194
6	3.669047478	11.554387225	23.030964815
1	3.568372814	12.486034511	22.478373429
6	3.302801079	11.505325244	24.380571637
6	3.984921462	9.155685251	24.454371608
6	4.320378306	9.228297540	23.096298727
1	4.722016437	8.350296316	22.596649760
6	2.888677245	6.431364230	16.990334675
6	2.982550429	5.417150309	17.956928459
1	3.664016734	5.555339157	18.792662521
6	2.238668423	4.241315469	17.841079688
6	1.377939838	4.101409184	16.741614309
1	0.782567296	3.193821260	16.646144990
6	1.265998183	5.093257150	15.762426399
6	2.035698253	6.258427211	15.896235707
1	1.965985015	7.040704505	15.142732875
6	7.657493948	9.950329234	13.312632667
6	6.926148813	9.643614834	12.160736933
1	5.848541605	9.510809993	12.233401432
6	7.561493668	9.499124091	10.918982685
6	8.949545815	9.653826851	10.857588830
1	9.453864779	9.535246013	9.899134657
6	9.710820448	9.953714956	11.997364628
6	9.048968619	10.107542034	13.216886349
1	9.619407665	10.339956071	14.111829369
6	8.105757925	14.567332837	18.402995960
6	9.458555693	14.664640883	18.049863939
1	9.965327158	13.794855067	17.639266730
6	9.487878082	16.966409154	18.751348461
6	8.136279517	16.900005973	19.111339219
6	7.457281870	15.688976839	18.936054045
1	6.408180546	15.614171488	19.214624172
1	4.307969078	7.014521635	24.583790826
6	2.365043667	3.139043830	18.869624751
1	2.865913882	2.258139508	18.446419309
1	2.948903453	3.465565114	19.736011319
1	1.381450719	2.807918222	19.225995309
6	0.351246687	4.911267880	14.571603690
1	-0.360996084	4.095975044	14.735744293
1	-0.221247321	5.822594875	14.361750147
1	0.921662452	4.672972273	13.663850685
6	6.753956376	9.188802707	9.677786767
1	7.401154483	8.939249525	8.830466999
1	6.074951066	8.343291163	9.843374391
1	6.133846539	10.044945865	9.380571664
6	11.213897345	10.095678040	11.905246767
1	11.500648858	10.823091478	11.135201004
1	11.643052141	10.421440222	12.857972756
1	11.686262979	9.141645348	11.637189836
6	7.435610352	18.106884687	19.694622067
1	7.645982845	19.012764811	19.113804025
1	6.350301911	17.966846407	19.718310009
1	7.766679217	18.300182897	20.723377540
7	10.590300729	9.293646682	19.282977466
1	9.586936917	9.124714863	19.169781569
6	11.072903917	11.488458553	16.340554757
1	10.469015857	12.127222520	15.698565410
6	10.447830372	10.781260280	17.366538095
1	9.374693528	10.858729948	17.512135345
7	5.267764138	6.722661162	20.222501588
1	6.038419786	7.272475822	19.842577020
1	4.435468474	7.280702341	20.363769414
6	4.536964909	5.277631683	22.037138755
1	3.514956189	5.629698021	21.903182546
6	5.559438584	5.805811707	21.223451528
6	10.033205175	12.720462200	20.956308537
1	10.851103925	13.318700193	21.377220426
1	10.065835097	11.726444469	21.413331109
1	10.225868035	12.589338876	19.887461101
6	10.166317600	15.858910637	18.226910358
1	10.026229096	17.904695225	18.883726881
6	11.633850285	15.947732243	17.871316767
1	11.961262100	15.067330419	17.309982406
1	11.847234005	16.837539205	17.266193002
1	12.256512594	16.014525573	18.773065051
1	11.083815672	8.474283847	19.615207310
6	11.198302150	9.947409927	18.220421328
6	12.584316125	9.842923526	17.993817874
6	13.195856266	10.552242094	16.963755507
1	14.268840907	10.450673180	16.814095895
6	12.451213441	11.386024393	16.127129427
1	12.933212860	11.942356357	15.326938716
6	4.824030385	4.307231237	22.996925834
6	6.127633139	3.840357992	23.174229586
1	6.348131850	3.084533992	23.923562715
6	7.144457572	4.363583210	22.370607957
1	8.167720234	4.014760327	22.495301188
6	6.872351172	5.332371891	21.408804928
6	3.471876658	10.300903143	25.075363599
1	3.203229969	10.255502466	26.130471472
6	2.727630715	12.719071801	25.075776515
1	1.635302580	12.646833434	25.164590581
1	3.126822223	12.825058176	26.091295405
1	2.952545370	13.639206727	24.526530762
6	4.203983282	7.882591191	25.241912303
1	3.375983172	7.692871307	25.935348682
1	5.120224166	7.946851865	25.844586744
1	4.016261765	3.912056064	23.609777690
1	7.670644843	5.732440675	20.787588741
1	13.180259416	9.201302826	18.640527249

^{6}4{B3LYP}$_{optBS1}$

26	5.535060874	10.206283217	17.938952353
17	7.323851815	8.733275257	18.828385701
7	5.711570713	11.490380905	19.415198562
7	3.868431301	9.532681196	18.764580243
7	5.219836210	9.011224796	16.393798789
7	6.639845629	11.388477598	16.800264306
6	6.840765835	12.321518873	19.573352094
6	7.171397842	12.390340176	20.998732730
6	6.169383496	11.720805985	21.683416644
6	5.209009264	11.254021093	20.688706714
6	3.895550046	10.780200266	20.879338441
6	3.180610913	10.156769361	19.818848482
6	1.783779863	9.854410099	19.713911210
6	1.643344683	8.936764408	18.645244932
6	2.950201617	8.707701323	18.126015176
6	3.410019293	7.631540565	17.259202848
6	4.590183369	7.754159291	16.547222678
6	5.327736955	6.764614630	15.761343300
6	6.285569455	7.451659110	15.032996898
6	6.157724142	8.865744446	15.379375462
6	6.757015117	9.988085418	14.772302028
6	6.765223774	11.264944649	15.405821929
6	7.099978053	12.545224225	14.852009100
6	7.288184340	13.429280937	15.939682262
6	7.057543634	12.674625337	17.131134957
6	7.374445315	13.025689184	18.505562716
6	8.436447052	12.924083305	21.624824084
1	8.728956563	13.870132867	21.169957949
1	8.248867247	13.141820913	22.682807121
6	6.189475860	11.392585733	23.156970165
1	6.917828488	12.041926035	23.654150446
1	5.226511730	11.604901665	23.625273215
6	6.568582429	9.918478241	23.438288898
1	6.615174753	9.736863069	24.518739535
1	5.831202894	9.232804869	23.009231141
1	7.542813192	9.670513079	23.004758444
6	0.637236026	10.477026718	20.472308225
1	-0.256475592	9.854740141	20.347289532
1	0.839338400	10.501624613	21.544397126
6	0.307376962	11.912967195	19.997810633
1	-0.550098583	12.314414777	20.551661986
1	1.159986860	12.581656203	20.158020625
1	0.063573441	11.929959423	18.929595350
6	0.331457452	8.409158628	18.116567272
1	0.401910766	7.342620281	17.893668876
1	-0.436142671	8.502592209	18.894222602
6	-0.156006950	9.149016951	16.848202011
1	-1.118663180	8.746546860	16.509406555
1	-0.282244175	10.220980429	17.039245753
1	0.566259934	9.038941627	16.030930050
6	5.206967955	5.262583515	15.831402627
1	4.167338266	4.944465396	15.730271726
1	5.744980199	4.823247410	14.984563416
6	5.784371307	4.672546648	17.140084904
1	5.729831003	3.577908470	17.117804412
1	6.828747637	4.963823723	17.284237390
1	5.220324835	5.023423907	18.009248813
6	7.361276245	6.807750224	14.191613606
1	7.013451070	5.823898795	13.856007937
1	7.549080845	7.384820384	13.286195486
6	8.694178541	6.629253446	14.958868610
1	9.443824699	6.156458516	14.313238454
1	9.090903758	7.593188269	15.292878368
1	8.560160809	6.001987823	15.846270027
6	7.117911640	12.957519023	13.399589526
1	7.665365052	13.901901479	13.301551677
1	7.657126625	12.232766982	12.787435212
6	5.700079192	13.149014603	12.808503435
1	5.760407015	13.475009658	11.762960900
1	5.133799086	12.211903712	12.841481082
1	5.135505750	13.902109435	13.369699410
6	7.574479653	14.905951436	15.803051011
1	8.303413156	15.233126898	16.544691176
1	8.037496267	15.093450030	14.827162166
6	6.309777086	15.787774517	15.926584217
1	6.564180193	16.847075692	15.799682409
1	5.564243614	15.522549544	15.168468555
1	5.844576705	15.665714298	16.910887694
6	3.234712683	10.926716109	22.209228961
6	2.962980237	12.202572902	22.739729782
1	3.248124237	13.083967774	22.173334763
6	2.330578923	12.349942363	23.981799001
6	2.248810301	9.907131137	24.202954006
6	2.867343025	9.789012109	22.946694723
1	3.070327117	8.804676805	22.535281674
6	2.598198729	6.379187355	17.252782402
6	2.436768621	5.656127854	18.452334574
1	2.926202463	6.013873615	19.353275463
6	1.658273867	4.494757302	18.498286749
6	1.014259226	4.070139469	17.319573531
1	0.396598142	3.175375990	17.345573447
6	1.143815234	4.775127236	16.115040425
6	1.947550059	5.930329604	16.093137646
1	2.056478389	6.494963655	15.171211736
6	7.423764706	9.845700927	13.442132978
6	6.671373361	9.558346947	12.290716900
1	5.596088736	9.428569266	12.376956763
6	7.289845612	9.442509776	11.034260485
6	8.682384426	9.602801216	10.955423540
1	9.172279426	9.506026966	9.989503196
6	9.460806770	9.883352141	12.092528006
6	8.817038588	10.010171903	13.331804281
1	9.401736026	10.222880996	14.219941886
6	8.278537802	14.186411497	18.767965307
6	9.645280457	14.134177782	18.454291201
1	10.059257995	13.237023206	18.006074266
6	9.927845714	16.385657858	19.286226435
6	8.560483930	16.467345015	19.603287369
6	7.748183129	15.355426626	19.345630387
1	6.690979800	15.392163595	19.590518286
1	1.505232446	7.876167532	24.363825130
6	1.502317332	3.708075787	19.783008856
1	1.896341067	2.688983158	19.677318053
1	2.034100173	4.186743976	20.611166877
1	0.447048461	3.617576114	20.069630776
6	0.439537597	4.309701790	14.857146341
1	-0.236834878	3.474583767	15.064430127
1	-0.151152292	5.117708862	14.408189068
1	1.157866782	3.974947744	14.097436636
6	6.467595349	9.145622411	9.797046560

1	7.093590501	9.118467559	8.899665149
1	5.957735754	8.177241226	9.878164949
1	5.692106414	9.906155579	9.641162001
6	10.959454639	10.069281479	11.982949728
1	11.221888733	11.132944680	11.899220588
1	11.474704267	9.682894577	12.868268481
1	11.361948360	9.560881787	11.100773948
6	7.986456817	17.727279797	20.217184629
1	8.205091131	18.607657429	19.600539216
1	6.900029464	17.657624775	20.327611563
1	8.411301088	17.916470697	21.211516127
7	10.430324638	9.255834661	18.583854705
1	9.410405850	9.209570624	18.565020005
6	11.249050618	11.665434207	15.895826528
1	10.720860989	12.290228798	15.180669172
6	10.506725311	10.857907788	16.763328352
1	9.421948194	10.855427200	16.722139120
7	7.536851134	5.704588791	20.305918094
1	7.485493828	6.618805327	19.870816072
1	6.674048877	5.212358846	20.458503583
6	8.789645485	3.826237475	21.216461802
1	7.860812026	3.295037337	21.409350895
6	8.741611603	5.114126955	20.632338516
6	9.619500461	11.929800154	21.524110068
1	10.520727399	12.367750990	21.969393435
1	9.395405383	10.996475994	22.051650089
1	9.840131848	11.675672577	20.483182550
6	10.486232308	15.228133556	18.721764351
1	10.569711227	17.241281538	19.484181875
6	11.969586282	15.140557089	18.431758871
1	12.171211867	14.490029825	17.574537897
1	12.395583029	16.128776372	18.226723504
1	12.513708341	14.719652081	19.288666400
1	10.899593201	8.664424649	19.248425153
6	11.158176507	10.036741091	17.716760915
6	12.574422767	10.058005935	17.756035724
6	13.300471245	10.868738979	16.882838984
1	14.386018983	10.864685504	16.936673909
6	12.650818431	11.683632768	15.943582325
1	13.221224866	12.309881900	15.265149174
6	10.012274665	3.237013237	21.544788618
6	11.220304809	3.904095837	21.303028095
1	12.168311438	3.441175691	21.557486784
6	11.179694626	5.180329812	20.722914500
1	12.105754333	5.713893128	20.525477890
6	9.964988461	5.782567686	20.393347214
6	1.983327708	11.191619117	24.701653421
1	1.497438641	11.294236898	25.669476776
6	2.017124061	13.722261630	24.540131354
1	0.935704511	13.912704992	24.546811173
1	2.368485809	13.821859694	25.574569496
1	2.489223496	14.511930606	23.947174185
6	1.900242271	8.671442667	25.005872060
1	1.150515943	8.890617302	25.773081759
1	2.785225992	8.266795487	25.515317905
1	10.019758703	2.246543907	21.992859242
1	9.945020569	6.769531265	19.942905102
1	13.094187303	9.435282037	18.479388831

^{6}4{B3LYP}$_{optBS2}$

26	5.838458260	10.133745803	17.871373744
17	7.518120827	8.710443595	18.718103376
7	5.837446985	11.622130497	19.317626594
7	4.132966558	9.392183056	18.769315420
7	5.334137485	9.027465831	16.188244707
7	6.735076324	11.498335534	16.605254776
6	6.863510927	12.539077898	19.379517349
6	7.300090036	12.689878236	20.763797443
6	6.474706108	11.889137565	21.530504772
6	5.515981511	11.278439843	20.616781589
6	4.343648837	10.554942219	20.921101522
6	3.595832079	9.825134830	19.962857449
6	2.246077545	9.295972525	20.099330529
6	2.024305596	8.476616280	19.007459117
6	3.248821520	8.485381957	18.217475143
6	3.605601729	7.608933932	17.162687608
6	4.726805100	7.797790703	16.322707130
6	5.383458625	6.833201417	15.443916079
6	6.361349458	7.523710163	14.754291665
6	6.273714902	8.915357508	15.181361463
6	6.906333835	10.048837269	14.630803004
6	6.922311615	11.324713759	15.252371788
6	7.244722787	12.608582652	14.642849988
6	7.323489091	13.530749344	15.670901886
6	7.077721267	12.803064340	16.909110738
6	7.304889383	13.242631432	18.233281182
6	8.506469512	13.426297210	21.298742973
1	8.602910641	14.409196514	20.839202207
1	8.357857169	13.611610918	22.368179929
6	6.689174326	11.598139604	22.997494368
1	7.293456073	12.400643431	23.433821721
1	5.748274500	11.600541631	23.546139884
6	7.409137605	10.254927806	23.240124764
1	7.556178320	10.090737027	24.314872914
1	6.829454938	9.414286012	22.846161224
1	8.391540745	10.238499351	22.755732637
6	1.194876155	9.658590789	21.121639545
1	0.421415634	8.883243980	21.125823810
1	1.611548040	9.666690717	22.127613206
6	0.525511953	11.018720487	20.842797390
1	-0.234121462	11.235832753	21.603776787
1	1.260189009	11.830629645	20.857607330
1	0.036617733	11.028627451	19.861921517
6	0.690232574	7.859829552	18.657375697
1	0.799208885	6.822445038	18.342656691
1	0.063237717	7.835228826	19.554609486
6	-0.058478228	8.641291797	17.559170130
1	-1.026625839	8.171629402	17.345702384
1	-0.242701130	9.677606461	17.864559973
1	0.518112877	8.666346104	16.628270631
6	5.190528353	5.335908353	15.364534550
1	4.136327106	5.069607277	15.425230334
1	5.527525763	4.990496784	14.380526527
6	5.967223226	4.561865883	16.448952815
1	5.780707054	3.484733113	16.356899675
1	7.046051406	4.727903662	16.362945661
1	5.665594062	4.873110244	17.454156547
6	7.411205227	6.873448747	13.883893258
1	7.050103189	5.888621848	13.568809253
1	7.577723856	7.439264782	12.967663471
6	8.760315582	6.690721068	14.609675215
1	9.483001194	6.196638063	13.948698435
1	9.182651478	7.653259091	14.913189408
1	8.648499898	6.079964007	15.511729374
6	7.317060148	12.955488140	13.173905570
1	7.840819838	13.910638355	13.060920288
1	7.909373560	12.225270278	12.622799920
6	5.928228020	13.076725958	12.515814801
1	6.026587922	13.337029628	11.454724676
1	5.374868845	12.133812766	12.582899084
1	5.324554149	13.852146088	13.001166063
6	7.455220378	15.023350222	15.477009669
1	8.165601962	15.457290013	16.180409135
1	7.862594269	15.218703775	14.479628463
6	6.107310028	15.759841584	15.608356271
1	6.240408180	16.835146157	15.439595004
1	5.377523095	15.388352226	14.879912656
1	5.680369000	15.625572582	16.607604025
6	3.871819535	10.527062673	22.340529691
6	3.331858230	11.672628395	22.942801012
1	3.260881453	12.593403884	22.367626510
6	2.883326193	11.645156144	24.266218377
6	3.555419528	9.296439786	24.418724323
6	3.974335479	9.343806201	23.082180061
1	4.402925452	8.462307970	22.607947993
6	2.779639527	6.375116284	16.972437441
6	2.837118606	5.342419641	17.920660165
1	3.487380836	5.453935561	18.785536797
6	2.089010439	4.173211212	17.766306259
6	1.252281097	4.062132206	16.646202088
1	0.653008838	3.161131929	16.521028170
6	1.169885131	5.073917621	15.684282479
6	1.947777195	6.227139874	15.857633739
1	1.905543618	7.023381566	15.117301769
6	7.645563821	9.908994180	13.338767677
6	6.978051465	9.685885278	12.130147292
1	5.893052255	9.602488834	12.127710296
6	7.686755362	9.574483549	10.925611676
6	9.081387297	9.672068209	10.959553553
1	9.641268823	9.580635234	10.029537091
6	9.779226791	9.886569226	12.157583064
6	9.045606510	10.012114183	13.339196215
1	9.563140625	10.188353438	14.279201874
6	8.075378075	14.508491074	18.441130386
6	9.443266849	14.572472557	18.140848262
1	9.945667750	13.693136038	17.748626502
6	9.498924790	16.872481868	18.850231912
6	8.132484440	16.839075374	19.155473505
6	7.433300734	15.645279209	18.950037566
1	6.373093962	15.593391815	19.188164384
1	3.602066517	7.136970803	24.606606313
6	2.191509449	3.041938107	18.764959848
1	2.826539684	2.232162302	18.380787673
1	2.629727993	3.378607727	19.710339214
1	1.208953837	2.606632471	18.981730264
6	0.279967126	4.924235231	14.470713997
1	-0.439995621	4.109643530	14.600002001
1	-0.282045154	5.843802446	14.270000167
1	0.869885024	4.704158210	13.570998401
6	6.950845119	9.369318195	9.620077380
1	7.640002004	9.121319872	8.806543158
1	6.215180477	8.559999600	9.697139366
1	6.403126846	10.274001904	9.325658721
6	11.289482513	9.969150610	12.168578741
1	11.658080532	10.659065229	11.399883229
1	11.663831375	10.309054533	13.139327980
1	11.740931239	8.989667269	11.964433904
6	7.439288154	18.059028580	19.719881245
1	7.728700847	18.970219938	19.183161703
1	6.350165993	17.963994685	19.660826302
1	7.700360015	18.210381320	20.775845983
7	10.611875572	9.219266549	19.231919808
1	9.612026794	9.050730923	19.104999884
6	11.106587442	11.533665635	16.383403199
1	10.501514012	12.177413361	15.747575626
6	10.475786644	10.775531520	17.368799801
1	9.397966250	10.827116119	17.493350988
7	5.525382320	7.068612643	20.882201683
1	6.041628550	7.669496972	20.241315023
1	4.643755584	6.777419399	20.476256057
6	5.670558430	4.838517150	21.860642512
1	4.600177551	4.703648934	21.708214336
6	6.280100401	6.033455123	21.439684635
6	9.826749184	12.652072632	21.111739472
1	10.662028767	13.219009593	21.541748288
1	9.792726023	11.672618597	21.599514335
1	10.045274189	12.479640238	20.053680883
6	10.171368394	15.749606181	18.352073448
1	10.054574672	17.796609262	19.007107993
6	11.657193715	15.801191438	18.071109678
1	11.965349659	14.995092539	17.397887790
1	11.947508950	16.756857314	17.618700472
1	12.237700125	15.691991833	18.997149352
1	11.109356361	8.392609226	19.538545220
6	11.226402263	9.928105931	18.209143557
6	12.620105774	9.865623293	18.015047142
6	13.238011682	10.627442729	17.027565916
1	14.316825800	10.558983439	16.902810547
6	12.492123473	11.472034983	16.201950298
1	12.979136038	12.067851327	15.434161756
6	6.420729948	3.834079351	22.469264026
6	7.792359920	3.993827600	22.672392492
1	8.376351614	3.208120259	23.144704300
6	8.401951414	5.180621573	22.255824042
1	9.470538260	5.322790308	22.404227145
6	7.662149776	6.191457032	21.648182969
6	3.007548814	10.450051660	24.989830678
1	2.669907588	10.421225434	26.025585884
6	2.262062178	12.866689414	24.906007845
1	1.169609683	12.771322995	24.968259807
1	2.630418552	13.014486179	25.928186806
1	2.481134868	13.774139100	24.333829179
6	3.724535566	8.029920001	25.228928322
1	3.001749050	7.977399421	26.050640563
1	4.728763169	7.975530303	25.670516698
1	5.926751985	2.916663683	22.783562060
1	8.144075533	7.111442493	21.326787149
1	13.216283558	9.216971857	18.654573104

^{2}1{BP86}$_{optBS1}$

6	8.126632829	30.129976642	31.146585538
6	7.579257045	30.143231906	29.736449850
1	7.619977175	29.117392179	29.323885454
1	6.516740970	30.433615474	29.728493795
6	8.365179668	31.091454713	28.792093259
1	7.946998615	31.056741794	27.769348747
1	8.311482325	32.134702783	29.150198911
1	9.431158613	30.806187081	28.740710399
6	9.555485409	27.989198456	30.896267911
1	8.911549657	27.793421035	30.018213237
6	11.008414074	28.228596165	30.405423423
1	11.372094773	27.359457466	29.826722811
1	11.067128754	29.123926066	29.760662878
6	5.856290444	32.214219490	31.330264221
6	5.689038617	33.160520842	30.292718587
1	6.461063967	33.922175285	30.137254645
6	4.547694081	33.135836743	29.460168026
6	3.567040290	32.144321090	29.688790014
1	2.678144749	32.113965908	29.046250557
6	3.702324082	31.189732969	30.723804397
6	4.858839398	31.230785276	31.532910756
6	4.367532011	34.170357706	28.362615770
1	5.335929248	34.462135530	27.919384158
1	3.720275817	33.791230861	27.552515858
1	3.896818716	35.092881503	28.755541054
6	2.614417297	30.158899034	30.969640061
1	1.785308253	30.587321351	31.566097968
1	2.179339291	29.795680297	30.020965173
1	3.002134347	29.287291831	31.524492030
6	10.251529683	24.567326928	33.705166788
1	10.329518391	23.476356936	33.619688427
6	12.797971464	24.680502482	33.631699506
1	13.190710109	24.443635344	34.639791147
1	12.752682769	23.732555084	33.067066129
6	7.713689865	24.311141404	33.776642819
1	7.938411304	23.249931979	33.979769810
1	7.215549911	24.362702074	32.789078714
26	9.604262886	32.233059251	34.465106929
7	8.555396552	33.628501045	33.589145965
7	8.886721280	30.920556776	33.216637296
7	10.717259125	30.849465323	35.265325443
7	10.876042048	33.595843407	35.058341535
17	8.140274447	32.211760515	36.264524267
6	8.541596496	34.971030622	33.996154024
6	7.180545311	35.496512133	33.907523263
6	6.391027241	34.485556039	33.345442838
6	7.282508248	33.361469778	33.055546656
6	7.071059192	32.249666673	32.209487723
6	7.998412426	31.181363766	32.158033571
6	8.996444168	29.166237350	31.665295157
6	9.381421486	29.615737468	33.005145216
6	9.948075055	28.859568295	34.056442508
6	10.403934405	29.480693830	35.243691126
6	10.753765361	28.879536729	36.529154765
6	11.378466915	29.876551779	37.289359869
6	11.438221333	31.075569950	36.452639410
6	12.223866099	32.237186112	36.614191457
6	12.063660988	33.364274024	35.768835219
6	12.972191996	34.500119906	35.580873946
6	12.269913830	35.470891602	34.861725579
6	10.921380232	34.942438148	34.638045679
6	9.747632717	35.649253118	34.292728796
6	6.655112646	36.803797348	34.458554822
1	5.688562945	37.037689203	33.972763789
1	7.330393620	37.637241297	34.209095945
6	6.444996200	36.768220228	35.996162233
1	6.050699601	37.736421850	36.356756783
1	5.733333024	35.973513707	36.281025404
1	7.396400027	36.564953541	36.517770395
6	4.882071558	34.513764367	33.244432034
1	4.533649923	35.558735197	33.347700737
1	4.538933459	34.170861466	32.254718861
6	4.196735470	33.649757912	34.338064459
1	3.096492163	33.726255102	34.257438005
1	4.479521398	32.588048534	34.234469239
1	4.498153739	33.976619361	35.348513417

1	9.533448555	27.070326115	31.503512731
1	11.690709571	28.382433956	31.260080914
6	10.384282060	27.504412323	37.040269468
1	10.553488259	26.737562384	36.268332362
1	11.050860989	27.241791264	37.883902110
6	8.911385178	27.416937835	37.522698076
1	8.685995987	26.402185480	37.899923456
1	8.715325120	28.141606231	38.332380159
1	8.214074633	27.642937494	36.697219180
6	11.741842027	29.753878074	38.752679679
1	11.774393807	28.682584493	39.027125089
1	12.749783752	30.153031645	38.950615590
6	10.727471962	30.475123636	39.681781906
1	11.009094018	30.336097155	40.742079203
1	10.700458178	31.557517576	39.468737733
1	9.706235596	30.082170086	39.535570454
6	14.443923110	34.566460116	35.926426040
1	14.770586482	35.623367310	35.908962671
1	14.627362673	34.207262895	36.951562828
6	15.331502659	33.755219336	34.945209607
1	16.398164059	33.842046528	35.221831178
1	15.058788144	32.685468672	34.962211876
1	15.210704564	34.118499139	33.909158322
6	12.868418281	36.735378682	34.285144225
1	12.220369628	37.605914391	34.474273400
1	13.825369626	36.947053513	34.798016494
6	13.133069606	36.632581296	32.759684460
1	13.599535141	37.561098201	32.382242516
1	13.806718017	35.787637552	32.530092281
1	12.190074544	36.473515277	32.208044305
1	4.992526750	30.494961734	32.333145946
6	10.050906496	27.368155280	33.925428617
6	8.886590504	26.566788032	33.908864237
1	7.906598757	27.050965623	33.981563655
6	8.972507554	25.160121876	33.803986246
6	11.433909490	25.343602916	33.713858500
6	11.318237536	26.745838630	33.829252107
1	12.219666736	27.368221781	33.847799718
1	13.536405898	25.337325558	33.139539798
6	13.269710228	32.281495411	37.689007339
6	13.132925906	33.171012274	38.777809944
1	12.253531072	33.822429776	38.825490440
6	14.109305248	33.227439393	39.799542479
6	15.233997226	32.379132109	39.706049291
1	15.998042520	32.416437214	40.492718674
6	15.400134905	31.480766935	38.624617799
6	14.406565389	31.439473357	37.624552759
1	14.507796032	30.745646951	36.782927203
6	16.611589915	30.567240069	38.561916477
1	16.542701672	29.757347620	39.314016733
1	16.706365263	30.092670652	37.570488118
1	17.546782361	31.120563703	38.765882269
6	13.958132761	34.199774415	40.956252600
1	12.908753577	34.262402707	41.295030113
1	14.578569161	33.900790638	41.818714671
1	14.267377616	35.222142566	40.664035212
6	9.761603578	37.148397954	34.234929019
6	9.590710048	37.806227422	32.992425356
1	9.455160255	37.206837901	32.086037931
6	9.581819847	39.214590248	32.908374506
6	9.733642225	39.963164085	34.099919112
6	9.901117049	39.335312016	35.354454305
6	9.918042385	37.922214166	35.406991489
1	10.044513614	37.413436517	36.368791456
1	6.980240548	24.654644130	34.528099839
6	10.078839589	40.158907648	36.618432843
1	11.150250898	40.344864426	36.829517594
1	9.585876821	41.143492663	36.530716658
1	9.657464625	39.642102005	37.499039059
6	9.402759490	39.917328559	31.573325925
1	8.505781857	40.564809558	31.575782049
1	10.268836979	40.564569478	31.338352178
1	9.292147425	39.192378141	30.749125135
1	9.715931960	41.059231798	34.049746660

^{2}1{BP86}$_{optBS3}$

6	8.140465100	30.034336958	31.180570271
6	7.663264979	29.989385323	29.746382097
1	7.673351994	28.939848458	29.399144813
1	6.619470084	30.327242084	29.659639705
6	8.544815630	30.833341871	28.797057249
1	8.181252392	30.755813452	27.755727558
1	8.529138792	31.898979027	29.085924675
1	9.596256880	30.494699889	28.820927206
6	9.376941573	27.777273664	31.129198624
1	8.701120879	27.546708737	30.285054922
6	10.823381001	27.878221947	30.593032935
1	11.122117507	26.937374457	30.094961337
1	10.922813199	28.701084784	29.862453674
6	6.028140701	32.231537310	31.110057762
6	6.003435066	33.110969155	30.008914832
1	6.833708808	33.814964594	29.870584593
6	4.935929598	33.097223165	29.090423960
6	3.882448061	32.186997572	29.301610560
1	3.045636628	32.164259985	28.589678304
6	3.871726941	31.302480217	30.399163289
6	4.959537001	31.329007099	31.291233961
6	4.910973132	34.057850464	27.917414689
1	5.926549666	34.247776377	27.527040151
1	4.293237960	33.669216949	27.088705722
1	4.486959899	35.038129582	28.210588437
6	2.705893512	30.358359796	30.619081918
1	1.877319587	30.862930279	31.153298885
1	2.296755695	29.988733723	29.661605027
1	3.001209600	29.484372490	31.225312781
6	9.932554948	24.569451359	34.078072777
1	9.956870862	23.471181634	34.051589702
6	12.462346186	24.553416126	33.812075520
1	12.920685200	24.359923287	34.801134875
1	12.333812948	23.575349247	33.315411408
6	7.405120557	24.434992646	34.346420090
1	7.596394662	23.381144768	34.613407033
1	6.848474807	24.438446579	33.389418905
26	9.592168677	32.249220012	34.457082056
7	8.549049206	33.644723405	33.537589232
7	8.874224159	30.903942124	33.221258551
7	10.722180433	30.872947092	35.277180293
7	10.855044591	33.634745982	35.062738608
17	8.140297602	32.225473746	36.190849797
6	8.475077257	34.961080839	33.986709699
6	7.124245977	35.477966291	33.804277588
6	6.409183633	34.487547566	33.135587979
6	7.334784753	33.379896159	32.915364444
6	7.171102045	32.253112173	32.076712114
6	8.052306390	31.148624762	32.122448835
6	8.909069562	29.052392492	31.794771474
6	9.292508059	29.578525746	33.102257217
6	9.842455389	28.868614267	34.193518124
6	10.369282411	29.526568556	35.327304962
6	10.785777185	28.948727079	36.598088281
6	11.499483762	29.939031230	37.269273292
6	11.508423909	31.110832962	36.400240504
6	12.298477971	32.278225843	36.510762954
6	12.066976197	33.423946856	35.715753947
6	12.922533787	34.601601007	35.563588117
6	12.155945223	35.570404964	34.927248491
6	10.837404219	34.982153492	34.711300050
6	9.637559308	35.655352940	34.384583334
6	6.526953631	36.754731860	34.352080974
1	5.602387319	36.986617685	33.791211639
1	7.199532971	37.611439875	34.194156580
6	6.184800862	36.656063289	35.857023307
1	5.735738951	37.601019408	36.215339188
1	5.470649200	35.836931952	36.052551319
1	7.090302040	36.455870028	36.455655599
6	4.922137214	34.530110960	32.865205887
1	4.582340301	35.581678444	32.902173259
1	4.687500362	34.169798579	31.851668051
6	4.105940167	33.709252756	33.891208546
1	3.023963926	33.786304969	33.675899145
1	4.388269785	32.642772122	33.859286225
1	4.281163331	34.068042833	34.920636762
1	9.308973547	26.918715918	31.814613363
1	11.536103179	28.071257928	31.414080578
6	10.408160517	27.608286683	37.187534994
1	10.517169582	26.800780899	36.448211713
1	11.110989997	27.366975128	38.006474480
6	8.967140062	27.587242696	37.747974674
1	8.735997299	26.599357170	38.187918845
1	8.830895800	28.355154277	38.529702733
1	8.230137787	27.790964316	36.951864486
6	12.000835139	29.825496169	38.690998496
1	12.077255752	28.755421936	38.958387063
1	13.016762224	30.236228650	38.791890603
6	11.070541733	30.524708246	39.709674568
1	11.460074206	30.402910137	40.737447855
1	10.990579038	31.604732054	39.496811944
1	10.050316649	30.103917869	39.673356938
6	14.399395125	34.730746541	35.862030446
1	14.657521447	35.804384056	35.922023735
1	14.648762998	34.303555370	36.845135697
6	15.288246347	34.066370040	34.785600363
1	16.359041713	34.201147008	35.025824457
1	15.086389727	32.982743573	34.721274489
1	15.101961715	34.502642484	33.787795106
6	12.676500082	36.887828950	34.397460488
1	11.993583868	37.717568572	34.638852879
1	13.632122617	37.123212706	34.901265335
6	12.915366691	36.863850047	32.870083125
1	13.324073970	37.831088552	32.523711790
1	13.628687172	36.068140433	32.589280213
1	11.973602673	36.676577842	32.324913051
1	4.986545595	30.637462544	32.142546815
6	9.871071711	27.373086270	34.148478171
6	8.679393205	26.631237536	34.263582500
1	7.728224947	27.166374117	34.376094825
6	8.695261391	25.223146306	34.235392701
6	11.141342544	25.283743010	33.953876019
6	11.094860284	26.688938969	33.996317890
1	12.022580148	27.269144052	33.915573002
1	13.189842112	25.141961216	33.225826031
6	13.440546859	32.276522104	37.479428464
6	13.401445720	33.047891247	38.655569892
1	12.521565604	33.672015118	38.855773954
6	14.470666694	33.022812704	39.575132637
6	15.588752339	32.220595587	39.283732241
1	16.428049913	32.197348015	39.992728095
6	15.661756076	31.444117841	38.107982380
6	14.575092779	31.477772381	37.218399384
1	14.597406757	30.870352668	36.305050475
6	16.874610977	30.578376227	37.830164566
1	16.909595922	29.705395049	38.509757718
1	16.867997705	30.194171827	36.795726739
1	17.815467753	31.139666028	37.978273756
6	14.417428428	33.857637868	40.839185577
1	13.442837211	33.755555932	41.349963311
1	15.207478737	33.564039069	41.551779982
1	14.553320246	34.932818149	40.615630667
6	9.603967375	37.148055406	34.478899545
6	9.500874720	37.933687132	33.310672839
1	9.442646463	37.432341560	32.336542521
6	9.460509994	39.336367870	33.378419699
6	9.507786702	39.946827870	34.649642466
6	9.608534670	39.191867360	35.832593640
6	9.664963288	37.786399256	35.732511366
1	9.746569207	37.175364865	36.639888624
1	6.733970265	24.864594975	35.111618783
6	9.674426863	39.870236773	37.186683880
1	10.716949588	39.931497798	37.553543388
1	9.279194397	40.900134597	37.143508431
1	9.096645354	39.313944779	37.946208666
6	9.355494448	40.178187339	32.122056304
1	8.452371433	40.817002475	32.137332419
1	10.225396930	40.853097508	32.013106167
1	9.305763773	39.546377274	31.219045955
1	9.461171482	41.042693170	34.718309843

^{4}1{BP86}$_{optBS1}$

6	8.257779351	30.126080291	31.091185422
6	7.830356595	30.139983391	29.640173070
1	7.918782119	29.117325704	29.228015998
1	6.768717799	30.418973526	29.542644646
6	8.682674622	31.104223888	28.771488259
1	8.363176939	31.060817438	27.714025385
1	8.574218828	32.146164756	29.121006890
1	9.754311995	30.840520815	28.821394404
6	9.589260790	27.906681925	31.006037548
1	8.987389321	27.701817067	30.101130994
6	11.077836897	28.065681012	30.593398232
1	11.436171657	27.160158905	30.069562731
1	11.212429333	28.932621880	29.922095154
6	5.992422861	32.205189391	31.132858615
6	5.823335636	33.150672632	30.095415718
1	6.562967709	33.951397170	29.977410823
6	4.719388912	33.075245349	29.213095828
6	3.781980606	32.032918837	29.390169851
1	2.923613751	31.964883536	28.708613678
6	3.921762630	31.074270782	30.423602917
6	5.034964507	31.170774807	31.284869725
6	4.536682805	34.111698673	28.116643780
1	5.505963250	34.410657427	27.677802609
1	3.895124795	33.730602022	27.302681505
1	4.059867362	35.030630281	28.510054694
6	2.894933603	29.966038129	30.590965354
1	1.882073379	30.377009005	30.764952181
1	2.836022264	29.331765840	29.685866229
1	3.143737916	29.313129174	31.445341096
6	9.943158410	24.558729274	34.008647616
1	9.964978383	23.462050992	33.976228543
6	12.491763042	24.532505045	34.084600254
1	12.810286526	24.322230707	35.124282769
1	12.425997510	23.562887557	33.560083394
6	7.395063467	24.439964606	33.949321228
1	7.566553361	23.361163885	34.108046417
1	6.894609616	24.559030235	32.969080616
26	9.570371669	32.262096501	34.482715113
7	8.559855022	33.648400103	33.541022629
7	8.886412646	30.923712133	33.197433948
7	10.717471664	30.883864125	35.261672634
7	10.862327058	33.639148296	35.076506340
17	7.996050802	32.155746023	36.289858946
6	8.494035995	34.970515746	34.013647917
6	7.121350736	35.458559497	33.894554314
6	6.389833454	34.465758065	33.227964775
6	7.320427915	33.376759356	32.933499816
6	7.166406057	32.273180487	32.063922688
6	8.093688331	31.199012519	32.072087174
6	9.040575197	29.135142419	31.697777555
6	9.353879486	29.606583427	33.046892655
6	9.859427356	28.872700458	34.149005477
6	10.336574055	29.531286488	35.305939555
6	10.677463037	28.975767744	36.615100060
6	11.388812291	29.966346130	37.306398026
6	11.480750493	31.122782998	36.417556532
6	12.307479028	32.264591416	36.519226715
6	12.104119581	33.393726550	35.686087364
6	12.986868703	34.539803157	35.461430654
6	12.218656184	35.530223227	34.836223587
6	10.868427351	34.986775474	34.680327364
6	9.665813342	35.673639739	34.376606556
6	6.527411265	36.700162509	34.522832809
1	5.557812721	36.921841494	34.037962712
1	7.166106085	37.580367327	34.347113098
6	6.297721068	36.543074396	36.051082848
1	5.837761139	37.458021459	36.468429861
1	5.634614633	35.687339000	36.266844988
1	7.252331807	36.363499048	36.575058849
6	4.890214212	34.475440841	33.029339349
1	4.519414360	35.512472511	33.134834657
1	4.620899377	34.155070859	32.009726979
6	4.144891523	33.572714046	34.050489071
1	3.051698016	33.638005633	33.896792965
1	4.448672366	32.517753773	33.937226435
1	4.372439064	33.874843391	35.087612428
1	9.492606437	27.015706998	31.646573939
1	11.715257551	28.223612634	31.480980543
6	10.210332726	27.664647396	37.207657148
1	10.348532985	26.834583999	36.496277924
1	10.835205576	27.423025519	38.088245385
6	8.721116560	27.707035929	37.647009746
1	8.423784784	26.742543926	38.099114589
1	8.547928631	28.509006324	38.385579934
1	8.062848270	27.904452371	36.783297555
6	11.804692542	29.891286712	38.758664533
1	11.815721801	28.831802663	39.077078260
1	12.832180527	30.264670106	38.898821262

6	10.850729740	30.684954113	39.692860970
1	11.166515718	30.578718092	40.747306953
1	10.852387394	31.758298031	39.435765425
1	9.811962144	30.323796188	39.599278738
6	14.482106452	34.604048610	35.683847250
1	14.809902575	35.659501690	35.632036694
1	14.749375252	34.249343147	36.692202681
6	15.281041287	33.781316051	34.636393896
1	16.368186052	33.874789896	34.814983142
1	15.014221123	32.711087113	34.692416085
1	15.066246878	34.130580138	33.610435522
6	12.754794292	36.830184746	34.276370096
1	12.089586228	37.673605601	34.522951310
1	13.727102887	37.055384534	34.754333388
6	12.953574423	36.782881037	32.736145585
1	13.371064765	37.738478595	32.367806014
1	13.644304851	35.969461537	32.449631400
1	11.991615151	36.606450570	32.222182110
1	5.170443610	30.436645200	32.087142163
6	9.886155513	27.374198633	34.092550771
6	8.682079744	26.635542780	34.040216828
1	7.727768819	27.173998041	34.032935243
6	8.695876797	25.222225200	34.004288468
6	11.164711428	25.271080379	34.054706455
6	11.120743786	26.680873262	34.099905571
1	12.052748930	27.255408059	34.143379322
1	13.293851012	25.124960732	33.610573350
6	13.425309019	32.276785894	37.517829834
6	13.421365311	33.194203986	38.592697822
1	12.582795101	33.891911759	38.694396882
6	14.477250297	33.219212634	39.532972478
6	15.543941506	32.307396913	39.375098832
1	16.368167182	32.319519742	40.099700869
6	15.573649006	31.374450495	38.310492496
6	14.503255442	31.366574081	37.392600241
1	14.497427874	30.646347795	36.566981670
6	16.733826761	30.404200865	38.167577980
1	16.877238180	29.809492684	39.089220418
1	16.568409384	29.700359412	37.334268604
1	17.684266533	30.937009650	37.974238053
6	14.470442808	34.223706800	40.672785740
1	13.448699137	34.387563968	41.059335035
1	15.103389665	33.886724875	41.511982775
1	14.856066486	35.208145659	40.342219370
6	9.639154629	37.170050607	34.456069039
6	9.398835394	37.948903724	33.299001925
1	9.223396003	37.442134532	32.343243630
6	9.378523609	39.359291813	33.360709673
6	9.602364107	39.985003725	34.609393754
6	9.841396904	39.234500102	35.783016709
6	9.857970955	37.824760746	35.691252101
1	10.043376620	37.221735791	36.586919095
1	6.683774519	24.788196062	34.720811071
6	10.103446868	39.930736219	37.107946899
1	11.176952539	40.178254543	37.225387066
1	9.539450330	40.877209670	37.185278092
1	9.818274622	39.292159822	37.961983623
6	9.091771330	40.189171631	32.121265138
1	8.034428077	40.517847048	32.096008367
1	9.716603421	41.100092690	32.090370210
1	9.280539630	39.613902993	31.198480937
1	9.588160161	41.080669495	34.669274730

^{4}1{BP86}$_{\mathrm{optBS3}}$

6	8.186602437	30.092281522	31.115266266
6	7.700555421	30.069734768	29.684427060
1	7.753522067	29.033507553	29.302710629
1	6.641498149	30.366283920	29.618317002
6	8.536421073	30.982403894	28.758063422
1	8.173630622	30.916138212	27.715865853
1	8.468704718	32.036806799	29.078552850
1	9.603057188	30.694642296	28.771514150
6	9.463779106	27.853310580	31.035988803
1	8.825857551	27.643017151	30.157798409
6	10.932713892	27.969713403	30.568548935
1	11.252273634	27.044467359	30.055009923
1	11.063168917	28.815757552	29.870162798
6	5.994367535	32.203604478	31.151020221
6	5.843181215	33.071459852	30.052974605
1	6.609897420	33.833373194	29.864005407
6	4.728079521	32.970909876	29.196974784
6	3.760042418	31.985975818	29.466444483
1	2.888235027	31.898690397	28.803174290
6	3.878490242	31.107435775	30.563290887
6	5.005428288	31.225570930	31.393036701
6	4.566757028	33.921789159	28.027012555
1	5.538124894	34.148025483	27.552174168
1	3.898119852	33.503252593	27.254557856
1	4.131488980	34.886882371	28.351613465
6	2.816714265	30.058343727	30.830383955
1	1.823996033	30.519972602	30.988938814
1	2.715262160	29.360377804	29.977900856
1	3.056502744	29.461668563	31.727116112
6	9.971146959	24.594057579	34.021277098
1	10.000276858	23.495945463	33.999223011
6	12.510049699	24.584428058	33.879036505
1	12.916153073	24.387298949	34.889965000
1	12.408945339	23.608368895	33.372183523
6	7.435680547	24.450944748	34.180668459
1	7.629387988	23.375961308	34.339253035
1	6.853469232	24.551003653	33.244870568
26	9.594222902	32.272308721	34.447929698
7	8.593392656	33.664392390	33.491345585
7	8.885781102	30.923859403	33.177649961
7	10.745067707	30.889796123	35.226706926
7	10.881045022	33.652250287	35.063894154
17	8.035700447	32.178639028	36.232181522
6	8.524037036	34.974006813	33.961136806
6	7.166091938	35.482185344	33.816830333
6	6.437698094	34.496680930	33.156002481
6	7.360296842	33.399666245	32.896580257
6	7.182457276	32.280282272	32.055438873
6	8.085397315	31.186946240	32.072462790
6	8.969550450	29.109487128	31.714607570
6	9.332580239	29.613400986	33.033827124
6	9.867252977	28.892322159	34.128765360
6	10.368018542	29.549930517	35.274889464
6	10.727272141	28.979350120	36.566058919
6	11.427412404	29.964509634	37.257508462
6	11.496433609	31.122423506	36.376325154
6	12.308947951	32.271881434	36.496234306
6	12.102701982	33.415168871	35.685895018
6	12.973085897	34.573283661	35.522096278
6	12.213625572	35.557893538	34.896319361
6	10.885502802	34.991478685	34.689379325
6	9.687582818	35.671252406	34.357014185
6	6.576074578	36.738579639	34.414741402
1	5.619870830	36.963634248	33.906860247
1	7.227837087	37.608543314	34.240047369
6	6.312683003	36.603869490	35.933080739
1	5.856476486	37.529843469	36.329376547
1	5.633034513	35.760720185	36.146896099
1	7.252233860	36.418419815	36.481367635
6	4.944004630	34.528126471	32.929077630
1	4.590640731	35.572839493	33.008612622
1	4.686924270	34.193453595	31.911864715
6	4.170077600	33.663610867	33.952089069
1	3.081160880	33.742759267	33.777561429
1	4.457157979	32.601579408	33.868814111
1	4.382418340	33.983990967	34.986835567
1	9.369278332	26.979650652	31.699278615
1	11.606579461	28.134970311	31.427444862
6	10.290315697	27.656303192	37.151134758
1	10.430051329	26.835509777	36.430919117
1	10.933605155	27.417017426	38.018204160
6	8.814993402	27.672610405	37.615679369
1	8.539928810	26.699124916	38.062178247
1	8.641889267	28.462507189	38.367143900
1	8.136557791	27.869137115	36.767688324
6	11.855230070	29.872132457	38.704050671
1	11.882027069	28.807940316	39.003120012
1	12.877894417	30.255439118	38.845131459
6	10.897831984	30.630605883	39.653255716
1	11.222495509	30.512698696	40.703623172
1	10.878288636	31.708061040	39.415649080
1	9.864238504	30.253660811	39.562617965
6	14.455803047	34.661297445	35.800391311
1	14.763152639	35.723221931	35.784591875
1	14.694964700	34.287875906	36.808619926
6	15.301294936	33.884463943	34.765144153
1	16.379642784	33.994853776	34.981953310
1	15.056339222	32.808140183	34.785221892
1	15.115115832	34.253514584	33.740563154
6	12.744953489	36.878987600	34.390161497
1	12.058945066	37.705163410	34.634776826
1	13.696284983	37.105986615	34.905814701
6	12.995785844	36.871847763	32.864663031
1	13.411335514	37.841380209	32.533905940
1	13.707508491	36.075829311	32.581017208
1	12.056061336	36.695547574	32.312460272
1	5.129216134	30.547735968	32.246519932
6	9.897530275	27.397747544	34.081518898
6	8.703810419	26.652002942	34.136395014
1	7.746448742	27.183708098	34.202891314
6	8.726256068	25.243701647	34.115666076
6	11.182138217	25.311915496	33.954880840
6	11.129546728	26.716125960	33.991320800
1	12.058210498	27.299163292	33.952576104
1	13.265871193	25.176767789	33.333991839
6	13.412576792	32.275304071	37.504584506
6	13.371876096	33.142506017	38.612857721
1	12.518196982	33.822087758	38.726858336
6	14.401638140	33.140785499	39.574620595
6	15.481465891	32.255964840	39.400439813
1	16.287734456	32.243970442	40.147029417
6	15.555043077	31.379508826	38.297663188
6	14.508925717	31.397660163	37.360053379
1	14.533122081	30.715024175	36.501548321
6	16.733880944	30.439173553	38.138169712
1	16.868153130	29.804860696	39.034342978
1	16.602546554	29.771410975	37.269412478
1	17.678628614	30.996688614	37.993339462
6	14.351438577	34.087959989	40.757631523
1	13.319054414	34.215897215	41.128862051
1	14.972889605	33.724152645	41.594519047
1	14.724530247	35.093719899	40.482827039
6	9.653684212	37.162462373	34.456214098
6	9.522756755	37.958954683	33.299877433
1	9.440453646	37.468445084	32.321907923
6	9.492027418	39.362783801	33.383783524
6	9.589824781	39.961927298	34.655296062
6	9.718264265	39.194509645	35.828728592
6	9.751776646	37.792376160	35.713178169
1	9.854837676	37.172098751	36.612086766
1	6.787080079	24.804078794	35.002978595
6	9.845789260	39.865334369	37.182552763
1	10.900916447	40.113211982	37.409641074
1	9.273926226	40.809622247	37.222121220
1	9.481467979	39.210817912	37.993465243
6	9.324841715	40.213866354	32.140264089
1	8.274191111	40.541242114	32.017266630
1	9.945091542	41.127132987	32.187051822
1	9.604865220	39.657288391	31.229220566
1	9.563240353	41.057668243	34.733618314

^{6}1{BP86}$_{\mathrm{optBS1}}$

6	8.192455633	30.065308499	31.116458167
6	7.776428691	30.063766504	29.661526707
1	7.838992135	29.031304409	29.268769763
1	6.723855923	30.369170568	29.550001151
6	8.661135683	30.986142587	28.780867734
1	8.339323759	30.939696582	27.724103667
1	8.589439282	32.035685672	29.116458544
1	9.723136138	30.686470328	28.833603531
6	9.510748588	27.842512485	31.043467324
1	8.888664861	27.622443027	30.155181833
6	10.986010746	28.007505367	30.589928709
1	11.337441457	27.096694067	30.070829056
1	11.096283646	28.863834143	29.900687968
6	6.001221826	32.215478105	31.071252224
6	5.884000618	33.134882790	30.004007217
1	6.650343493	33.908225986	29.880045957
6	4.801515683	33.063855848	29.096209448
6	3.828181928	32.057340119	29.282297196
1	2.985598232	31.993514448	28.582013850
6	3.912582387	31.128752453	30.348121650
6	5.008490689	31.216481328	31.230886489
6	4.679275623	34.067047577	27.961835367
1	5.665388373	34.302317614	27.522630705
1	4.027803305	33.687239387	27.155389421
1	4.244228654	35.021945959	28.316037731
6	2.846742494	30.060745003	30.526779820
1	1.843611097	30.509509112	30.654633630
1	2.792933645	29.393101798	29.645951628
1	3.050118199	29.434327186	31.412120635
6	9.955601188	24.529114361	34.000910812
1	9.979720497	23.433362119	33.954748522
6	12.505584400	24.510735251	34.018007006
1	12.862830232	24.316681822	35.048250618
1	12.424672261	23.533943586	33.509299574
6	7.407477850	24.402749514	34.003257232
1	7.584434492	23.325203584	34.161545826
1	6.891254207	24.517091779	33.030912353
26	9.426097150	32.257020087	34.645295644
7	8.550931295	33.714589144	33.445299385
7	8.806055126	30.869434132	33.221659928
7	10.810310730	30.831516693	35.254235386
7	10.849472648	33.690737325	35.155024865
17	7.901065492	32.159846648	36.402690533
6	8.482441959	35.026481435	33.933148987
6	7.108277544	35.524550340	33.780136717
6	6.386965955	34.532854487	33.102975456
6	7.319779504	33.428115378	32.839792137
6	7.156206601	32.275639195	32.025195165
6	8.037465836	31.156454032	32.086614436
6	8.968572678	29.076019701	31.732959997
6	9.289344269	29.560366143	33.081562971
6	9.864053160	28.841680465	34.168314729
6	10.416354454	29.487884778	35.307475543
6	10.791307059	28.927403973	36.612804226
6	11.507034186	29.920734440	37.292823462
6	11.571410696	31.087005993	36.402283590
6	12.352193539	32.269294011	36.513579432
6	12.101015396	33.433139812	35.732411278
6	12.977488767	34.589457966	35.506923259
6	12.200961848	35.577504456	34.888329752
6	10.848401253	35.026813066	34.734154443
6	9.651082279	35.708693745	34.367157348
6	6.502420534	36.766710935	34.396852915
1	5.536906949	36.981429247	33.900775336
1	7.139131349	37.648711639	34.222430494
6	6.257348638	36.619429150	35.922673189
1	5.789188483	37.534866561	36.329279136
1	5.594870203	35.762871172	36.137731964
1	7.206952099	36.449268377	36.458711353
6	4.890888846	34.551801271	32.875255236
1	4.527348577	35.593548454	32.956746268
1	4.639343972	34.217976822	31.855822082
6	4.116100101	33.673679715	33.894059465
1	3.027176834	33.743695857	33.715172467
1	4.413074559	32.614559448	33.806442976
1	4.321230421	33.993477486	34.930454217
1	9.436317985	26.957638626	31.695631592
1	11.645218127	28.185249887	31.457814248
6	10.339226711	27.616799307	37.219254570
1	10.475434866	26.783914689	36.511265343
1	10.977393352	27.384690667	38.093034407
6	8.856546545	27.648247476	37.678576888
1	8.574125784	26.684256596	38.140920313
1	8.685720574	28.452525913	38.415535976
1	8.183662951	27.832243310	36.823061110
6	11.943272793	29.841048805	38.739651112
1	11.967472119	28.779489433	39.050860542
1	12.969619542	30.221283724	38.867701840
6	10.997332747	30.618179654	39.694299263
1	11.330160760	30.507837109	40.743005333
1	10.985116745	31.693646995	39.446400576
1	9.960675016	30.246487061	39.615751785
6	14.475562151	34.661586167	35.710929764
1	14.794892301	35.720178128	35.671880895
1	14.758617614	34.293647178	36.710399135
6	15.267351256	33.862426884	34.641283643
1	16.355780007	33.961125657	34.809100243
1	15.009007116	32.789761833	34.682209933
1	15.038921040	34.226614382	33.623591765
6	12.735217537	36.876182186	34.323302451
1	12.063930341	37.717789631	34.558034217

1	13.702005445	37.110676459	34.808243131
6	12.948476477	36.820073896	32.786615280
1	13.359926776	37.777087961	32.415908176
1	13.649870265	36.011869331	32.511952456
1	11.993604496	36.630592173	32.265018265
1	5.101890032	30.503825477	32.057506672
6	9.892784693	27.343784219	34.114651930
6	8.689802175	26.601050947	34.085656078
1	7.734290012	27.136477286	34.106625096
6	8.706650233	25.188612573	34.034685819
6	11.175917889	25.244565510	34.026074776
6	11.128939315	26.653403575	34.087636170
1	12.059789192	27.230508378	34.114133821
1	13.287956381	25.099224343	33.507144007
6	13.489213190	32.280528271	37.489386424
6	13.494471191	33.176514126	38.582447421
1	12.652305574	33.865197653	38.710220639
6	14.559957317	33.185924452	39.511766610
6	15.629213280	32.282712994	39.322891796
1	16.459601968	32.281048725	40.039992048
6	15.653170375	31.375122967	38.236653295
6	14.572638490	31.381551050	37.331155966
1	14.561422632	30.679700942	36.490365890
6	16.818450893	30.417416032	38.057396194
1	16.984988926	29.809924772	38.966782704
1	16.643118315	29.724703261	37.216748096
1	17.760388757	30.961461736	37.853401932
6	14.560491387	34.163290662	40.674379351
1	13.539868629	34.327955527	41.062589002
1	15.188508749	33.800424728	41.506608411
1	14.956518984	35.151308621	40.368161277
6	9.624053679	37.204453872	34.460636723
6	9.409834983	37.999730185	33.309087264
1	9.255327912	37.506199731	32.343116508
6	9.391177725	39.409211176	33.389534129
6	9.585243889	40.017572297	34.651842606
6	9.794147125	39.251028385	35.820973434
6	9.812762967	37.842903057	35.709951008
1	9.971867486	37.227192217	36.601959793
1	6.707423941	24.750884194	34.784448740
6	10.023100263	39.928128165	37.161611788
1	11.090123824	40.191954497	37.300875552
1	9.442056731	40.863826079	37.246494181
1	9.734941120	39.269777235	37.999713144
6	9.137256715	40.257332051	32.155346254
1	8.081324881	40.588361312	32.107197705
1	9.764014816	41.167698458	32.153726636
1	9.349198824	39.695168774	31.229357373
1	9.571499323	41.112396185	34.725778721

^{6}1{BP86}$_{\text{optBS2}}$

6	8.112425708	30.049235007	31.138708213
6	7.647344323	30.022947611	29.699827405
1	7.681018940	28.981172438	29.331092560
1	6.597496323	30.342859187	29.611516807
6	8.520807884	30.901865232	28.774661744
1	8.167479396	30.837571824	27.728922315
1	8.483247139	31.960918443	29.084434555
1	9.578076129	30.582226732	28.801480585
6	9.386407784	27.812825248	31.051487326
1	8.730110221	27.593904868	30.189018581
6	10.842171448	27.947288063	30.547029370
1	11.160911522	27.024925759	30.027327966
1	10.945236344	28.792769580	29.843390988
6	5.981776844	32.213325283	31.108929149
6	5.871785237	33.047850023	29.979808920
1	6.663037331	33.780999591	29.778216659
6	4.769128078	32.947551874	29.107093210
6	3.769033545	31.999192228	29.393410116
1	2.906510014	31.912615434	28.717858087
6	3.844149306	31.155986885	30.522210140
6	4.961428431	31.270962668	31.360066905
6	4.655785230	33.859192052	27.900644963
1	5.637972309	34.021185814	27.421624731
1	3.970536348	33.442825717	27.141745348
1	4.265903039	34.855670208	28.185523412
6	2.748652128	30.146666647	30.806803466
1	1.765442096	30.639937239	30.923989286
1	2.646538131	29.417523097	29.980835984
1	2.952582343	29.580253768	31.731636447
6	10.042037901	24.562329382	33.982281173
1	10.089501017	23.465075551	33.948241799
6	12.580065793	24.599845667	33.809045818
1	13.015522676	24.426178931	34.812197948
1	12.486637015	23.614650539	33.318804173
6	7.510825892	24.374634076	34.173774600
1	7.723794455	23.303410492	34.333063436
1	6.922319533	24.462626082	33.240549804
26	9.433562419	32.262442621	34.621921338
7	8.573384416	33.727173034	33.409299188
7	8.790159536	30.869218127	33.208957461
7	10.823418532	30.831226767	35.223404819
7	10.853859977	33.693358198	35.156883206
17	7.927439951	32.174689901	36.347159860
6	8.512164847	35.027601939	33.885057981
6	7.158998276	35.558429895	33.702614767
6	6.432021561	34.575186556	33.036732237
6	7.350162541	33.455197959	32.814259270
6	7.157271601	32.287748748	32.031093830
6	8.012518921	31.149846795	32.098613194
6	8.890513705	29.063808536	31.741273667
6	9.256872148	29.570734647	33.064428829
6	9.868042899	28.860302694	34.133493273
6	10.439786454	29.500739255	35.266939422
6	10.836074519	28.912344940	36.548573096
6	11.537252159	29.898242409	37.238057976
6	11.571186089	31.073256919	36.365075235
6	12.330999247	32.265193557	36.508223346
6	12.080926176	33.442203183	35.750809668
6	12.953738956	34.604832806	35.583251659
6	12.190200329	35.589718395	34.961317246
6	10.857715414	35.019102863	34.756705049
6	9.673950200	35.697686492	34.351570257
6	6.569637729	36.828212690	34.274958596
1	5.624349970	37.054333510	33.747532403
1	7.231275139	37.690487236	34.099167574
6	6.277547856	36.720526170	35.790131951
1	5.822966273	37.656649277	36.163798765
1	5.584915541	35.888157807	36.005441199
1	7.204613642	36.536953135	36.359938376
6	4.942932981	34.629974407	32.778338043
1	4.611424303	35.684239932	32.815593578
1	4.699047630	34.268168023	31.767561349
6	4.126551251	33.818858886	33.812160910
1	3.043850881	33.910134875	33.607157020
1	4.393962735	32.748920469	33.777177325
1	4.316381742	34.175703450	34.839563453
1	9.318988597	26.937365906	31.714735283
1	11.536116358	28.123359747	31.387638956
6	10.421552107	27.579645202	37.131586648
1	10.550020931	26.766551844	36.400458140
1	11.088239940	27.336641512	37.980025961
6	8.958137052	27.576930404	37.632313300
1	8.702536435	26.596332846	38.074798234
1	8.795503995	28.355728480	38.397850428
1	8.255896544	27.775015933	36.804040036
6	11.990860604	29.791947068	38.676810040
1	12.044977314	28.723533501	38.957082296
1	13.007951168	30.193328903	38.806772434
6	11.036884179	30.514156752	39.657499618
1	11.385400371	30.388948730	40.699409025
1	10.987742687	31.594737561	39.438360378
1	10.010361771	30.114186863	39.584550771
6	14.441349904	34.696534407	35.840597186
1	14.741728221	35.760591127	35.839032881
1	14.699529130	34.308418030	36.837979684
6	15.277543438	33.944290583	34.779040913
1	16.358286432	34.055246771	34.984470522
1	15.038420992	32.866442850	34.778853048
1	15.077989432	34.332905648	33.764282939
6	12.728835283	36.905539340	34.445613095
1	12.043332719	37.736357511	34.673645290
1	13.674348895	37.137030372	34.969828862
6	12.998564570	36.882665467	32.922812139
1	13.414857075	37.850113055	32.586281876
1	13.716766937	36.086729438	32.655819237
1	12.067295926	36.696548261	32.359563475
1	5.052991456	30.618832359	32.243458056
6	9.920994218	27.365745153	34.074451297
6	8.741436085	26.597234844	34.138122597
1	7.776245906	27.111091131	34.223618048
6	8.787163757	25.189752985	34.101063127
6	11.239901668	25.301955310	33.907515930
6	11.162973191	26.704914649	33.959347693
1	12.080627142	27.304135459	33.915338863
1	13.310353483	25.198997050	33.237060345
6	13.449555784	32.277374790	37.500758622
6	13.400894347	33.118526757	38.629855889
1	12.531798589	33.773596372	38.769044892
6	14.440957943	33.121069120	39.580912378
6	15.541092727	32.267852610	39.374473519
1	16.355384371	32.258490357	40.112497859
6	15.625199336	31.420641564	38.249190405
6	14.567318969	31.432345272	37.324245900
1	14.599424444	30.770883090	36.449529745
6	16.828263495	30.518698813	38.052638612
1	17.001231401	29.877493367	38.937142393
1	16.698888461	29.858324026	37.177963844
1	17.752383939	31.106643672	37.894984917
6	14.380690304	34.039312707	40.786342248
1	13.347369040	34.147009877	41.161270205
1	15.007307883	33.663358098	41.614054913
1	14.742217965	35.055608504	40.535422219
6	9.657916492	37.190205493	34.446937636
6	9.560094443	37.986821572	33.286738296
1	9.488392299	37.495210679	32.308404660
6	9.552498820	39.391288906	33.366507788
6	9.637699602	39.991445872	34.639004295
6	9.732939537	39.225338665	35.817335868
6	9.745798746	37.821789795	35.705543328
1	9.824018901	37.201679143	36.607054408
1	6.859788850	24.717950640	34.998230411
6	9.848688008	39.899198302	37.170903423
1	10.901794467	40.148161304	37.406777157
1	9.276673983	40.843752223	37.203589129
1	9.477327189	39.246719062	37.980156315
6	9.423820129	40.242083588	32.117977854
1	8.381691819	40.587770223	31.974006520
1	10.059005748	41.144515374	32.174293980
1	9.711209145	39.678528573	31.213514804
1	9.628797802	41.087798942	34.714293506

^{2}2{BP86}$_{\text{optBS1}}$

7	5.931630000	6.696981000	11.374545000
7	8.643472000	6.279930000	11.337363000
6	6.179904000	7.069997000	10.037005000
6	5.028496000	7.811222000	9.522896000
6	4.022998000	7.724754000	10.491831000
6	4.569962000	6.952097000	11.608862000
6	3.878173000	6.368078000	12.700250000
6	9.970922000	5.886587000	11.590634000
6	10.487839000	5.151260000	10.436740000
6	9.515963000	5.234384000	9.429752000
6	8.399087000	5.999423000	9.979386000
6	7.283244000	6.556381000	9.314531000
6	11.747662000	4.318081000	10.359227000
1	12.606549000	4.852806000	10.793590000
1	11.998799000	4.143018000	9.296187000
6	11.600114000	2.944385000	11.069308000
1	12.532005000	2.358417000	10.969845000
1	10.772902000	2.357612000	10.633583000
1	11.388677000	3.075083000	12.144572000
6	9.592100000	4.527916000	8.094689000
1	10.649858000	4.285656000	7.879344000
1	9.257603000	5.188595000	7.279340000
6	8.761078000	3.217586000	8.052280000
1	8.901529000	2.706672000	7.082346000
1	7.684770000	3.425938000	8.172876000
1	9.056643000	2.523036000	8.856922000
6	4.965605000	8.656479000	8.270088000
1	3.910457000	8.915193000	8.063183000
1	5.320414000	8.092362000	7.393154000
6	5.785978000	9.968708000	8.389102000
1	5.689649000	10.571663000	7.467670000
6	6.856997000	9.750016000	8.545523000
1	5.437437000	10.580072000	9.240896000
6	2.695145000	8.446493000	10.434543000
1	1.875646000	7.790766000	10.767703000
1	2.472055000	8.706104000	9.382692000
6	2.678226000	9.746667000	11.282283000
1	1.698569000	10.252175000	11.199319000
1	3.460488000	10.450612000	10.945496000
1	2.860737000	9.523723000	12.348379000
6	7.227948000	6.565782000	7.814192000
6	6.309593000	5.725357000	7.140002000
1	5.660273000	5.063484000	7.722221000
6	6.238722000	5.711056000	5.729907000
6	7.093831000	6.571402000	5.001596000
1	7.047056000	6.569740000	3.905201000
6	8.013613000	7.427667000	5.648388000
6	8.078582000	7.406798000	7.060748000
1	8.796150000	8.050001000	7.582369000
6	5.268419000	4.790623000	5.009506000
1	5.791976000	4.157496000	4.268080000
1	4.500577000	5.367301000	4.457943000
1	4.749558000	4.122068000	5.717531000
6	8.897920000	8.364253000	4.842931000
1	9.808010000	8.641476000	5.402896000
1	8.365654000	9.303055000	4.593607000
1	9.210404000	7.903796000	3.888674000
6	2.386938000	6.230230000	12.601959000
6	1.831672000	5.317938000	11.673480000
1	2.499636000	4.706978000	11.057790000
6	0.433265000	5.165969000	11.551465000
6	-0.405937000	5.961711000	12.366350000
1	-1.494448000	5.856211000	12.276819000
6	14.401809000	6.922005000	12.055300000
6	12.994255000	7.020481000	11.965179000
1	12.540467000	7.705325000	11.240437000
6	-0.157461000	4.158631000	10.580856000
1	0.624562000	3.700730000	9.951585000
1	-0.903543000	4.629157000	9.913959000
1	-0.672154000	3.340347000	11.118972000
6	15.300280000	7.786979000	11.187928000
1	16.215988000	7.245821000	10.890438000
1	15.621094000	8.699360000	11.727609000
1	14.782666000	8.113229000	10.269503000
8	6.543618000	1.547697000	10.491033000
6	5.455746000	1.823451000	9.676203000
6	4.795886000	3.076496000	9.665008000
1	5.133723000	3.874283000	10.334845000
6	3.712326000	3.281330000	8.786188000
1	3.214331000	4.257709000	8.771323000
6	3.274988000	2.252730000	7.926245000
1	2.431212000	2.417525000	7.248710000
6	3.936480000	1.005257000	7.953351000
1	3.605166000	0.196575000	7.293334000
6	5.020440000	0.787307000	8.818790000
1	5.546669000	-0.170206000	8.851924000
1	6.763509000	2.321013000	11.090454000
26	7.268680000	6.239841000	12.724573000
17	7.162988000	3.824038000	12.623537000
7	8.613015000	6.671038000	14.062814000
7	5.893497000	6.295742000	14.099180000
6	8.365643000	7.062165000	15.397380000
6	9.516378000	7.809370000	15.901228000
6	10.518916000	7.723299000	14.927905000
6	9.974532000	6.939118000	13.819975000
6	10.663805000	6.359123000	12.727963000
6	4.563219000	5.903384000	13.843747000
6	4.044944000	5.171765000	14.997659000
6	5.018222000	5.251223000	16.005036000
6	6.135912000	6.015554000	15.458016000
6	7.257262000	6.564747000	16.123250000
6	2.784185000	4.340540000	15.076791000
1	1.928909000	4.870964000	14.630492000
1	2.525960000	4.177122000	16.139984000
6	2.936928000	2.959202000	14.382406000
1	2.003309000	2.375500000	14.479043000
1	3.759419000	2.375843000	14.831493000
1	3.158929000	3.079424000	13.308181000
6	4.941568000	4.540432000	17.337948000
1	3.884448000	4.291070000	17.548244000
1	5.269248000	5.201081000	18.156563000
6	5.780022000	3.234493000	17.380478000
1	5.633801000	2.717451000	18.346435000

1	6.856309000	3.450098000	17.271099000
1	5.496495000	2.542495000	16.569006000
6	9.584789000	8.657862000	17.151649000
1	10.641732000	8.909057000	17.358903000
1	9.224977000	8.098072000	18.029283000
6	8.774035000	9.975834000	17.029364000
1	8.873132000	10.578914000	17.950440000
1	7.701776000	9.764553000	16.871089000
1	9.128793000	10.583767000	16.177687000
6	11.844161000	8.450709000	14.978546000
1	12.664602000	7.797412000	14.642958000
1	12.070798000	8.712543000	16.029111000
6	11.853849000	9.749492000	14.128640000
1	12.832439000	10.257757000	14.207361000
1	11.070980000	10.451912000	14.466995000
1	11.668432000	9.524008000	13.063643000
6	7.306682000	6.583458000	17.623389000
6	8.222324000	5.748201000	18.307126000
1	8.875199000	5.083765000	17.731642000
6	8.282764000	5.739517000	19.717836000
6	7.420075000	6.599133000	20.436525000
1	7.457455000	6.599696000	21.532981000
6	6.503051000	7.450929000	19.779949000
6	6.448769000	7.425186000	18.368139000
1	5.732528000	8.063254000	17.838929000
6	9.258906000	4.831438000	20.444916000
1	8.772615000	4.314826000	21.292330000
1	10.109762000	5.406659000	20.859759000
1	9.671449000	4.064374000	19.768015000
6	5.609427000	8.385149000	20.576630000
1	4.711781000	8.672398000	20.002626000
1	6.142407000	9.318364000	20.844725000
1	5.276686000	7.917638000	21.520413000
6	12.158159000	6.245296000	12.802250000
6	12.743596000	5.349753000	13.728671000
1	12.095362000	4.736016000	14.362555000
6	14.145757000	5.216932000	13.825202000
6	14.958380000	6.015104000	12.986216000
1	16.049788000	5.924322000	13.054858000
6	0.120683000	6.884468000	13.298777000
6	1.524590000	7.002134000	13.413852000
1	1.955320000	7.701095000	14.139192000
6	14.768325000	4.230103000	14.797109000
1	13.999456000	3.736966000	15.414983000
1	15.485473000	4.728731000	15.476215000
1	15.324250000	3.437414000	14.261740000
6	-0.804249000	7.746700000	14.140328000
1	-1.718839000	7.196999000	14.426560000
1	-1.126107000	8.649712000	13.585174000
1	-0.307258000	8.088701000	15.064919000
8	7.842907000	1.592757000	14.780571000
6	8.932559000	1.853312000	15.597437000
6	9.599160000	3.102596000	15.623422000
1	9.267380000	3.911079000	14.963346000
6	10.682914000	3.291069000	16.505464000
1	11.186391000	4.264455000	16.531819000
6	11.114086000	2.250359000	17.354193000
1	11.958076000	2.402637000	18.034248000
6	10.446502000	1.006666000	17.311830000
1	10.773425000	0.188129000	17.962074000
6	9.362305000	0.804776000	16.443035000
1	8.831514000	-0.149699000	16.398217000
1	7.623700000	2.372624000	14.188834000

^{2}2{BP86}$_{optBS2}$

7	5.875440948	6.964390575	11.408659903
7	8.607449599	6.595037369	11.293980617
6	6.053770440	7.187390279	10.046914748
6	4.898522021	7.896792209	9.504131057
6	3.969631605	7.992054840	10.534576760
6	4.555328926	7.313320714	11.689649836
6	3.891005259	6.869899509	12.856679824
6	9.963844152	6.356852424	11.483415223
6	10.510473065	5.635243814	10.340544765
6	9.483128288	5.538103713	9.401736803
6	8.328004523	6.213953018	9.979341401
6	7.150369023	6.643299725	9.333225797
6	11.863940669	4.972971645	10.214242383
1	12.674671781	5.676144993	10.462716797
1	12.019643677	4.691627529	9.157096586
6	12.005988477	3.704053514	11.086878630
1	12.981285984	3.219095459	10.895227888
1	11.202778877	2.974937281	10.885348271
1	11.958339101	3.953877072	12.161365710
6	9.560473639	4.742701744	8.118256292
1	10.623789703	4.603271906	7.850309244
1	9.106774829	5.290209399	7.279005412
6	8.893555830	3.353031510	8.239327887
1	9.016304274	2.782914255	7.300381828
1	7.812181863	3.446801956	8.440642606
1	9.334814210	2.765875789	9.062088351
6	4.758303429	8.561941464	8.153592311
1	3.686808043	8.753933296	7.961208109
1	5.095012820	7.896154718	7.345357034
6	5.526220251	9.899814277	8.059330440
1	5.381782040	10.362986391	7.065922081
1	6.609155729	9.746429643	8.210445972
1	5.178738164	10.615553031	8.825777456
6	2.699437517	8.810339503	10.476434343
1	1.846828421	8.265662845	10.907975333
1	2.440508257	8.991767748	9.417487660
6	2.839295862	10.173565725	11.192694206
1	1.906116415	10.759112521	11.099290527
1	3.664250997	10.769760869	10.762923110
1	3.049375678	10.035581348	12.267801387
6	7.045701690	6.529169777	7.843995549
6	6.186391406	5.571506151	7.267983343
1	5.600781720	4.917299772	7.925686218
6	6.080697314	5.438300131	5.870863517
6	6.849366550	6.293878106	5.056335786
1	6.780309582	6.194361789	3.964177720
6	7.708295925	7.267929834	5.601470549
6	7.804223453	7.367109365	7.002837587
1	8.486224251	8.099348490	7.452841974
6	5.149700119	4.411854839	5.255354947
1	5.563245681	4.000592777	4.316945197
1	4.165859885	4.858119512	5.010213492
1	4.965114486	3.569049372	5.944249383
6	8.502861118	8.194665793	4.702480531
1	9.483508140	8.445423466	5.144121488
1	7.967267446	9.149490196	4.536403503
1	8.680187302	7.742391857	3.710948916
6	2.396527310	6.941471159	12.907480051
6	1.612477615	6.089936592	12.101998720
1	2.113851387	5.379131694	11.433442571
6	0.207016833	6.137601306	12.148181340
6	-0.404316269	7.079192810	13.000872530
1	-1.501389278	7.131018093	13.039289314
6	14.131758200	8.063615360	11.484618421
6	12.732591816	7.940247360	11.584788998
1	12.082264894	8.549160846	10.944454722
6	-0.633760124	5.184181611	11.321986585
1	-0.058668377	4.763479720	10.478951607
1	-1.529715869	5.683898399	10.910866861
1	-0.988940670	4.332566805	11.934339672
6	14.759820509	9.063596629	10.533917650
1	15.692972072	8.670947686	10.091873274
1	15.018245816	10.006084287	11.054778140
1	14.074186858	9.322533168	9.708485372
8	9.162338018	1.797623198	11.589908153
6	8.495156952	0.768695680	10.971145777
6	7.103926157	0.784733596	10.748752531
1	6.506711055	1.636205836	11.095675880
6	6.492716777	-0.304780567	10.111143526
1	5.407674935	-0.288695438	9.950893152
6	7.250706721	-1.408242669	9.688837022
1	6.766363385	-2.256951732	9.193282162
6	8.637354271	-1.415450075	9.917515992
1	9.241594688	-2.272834379	9.596412135
6	9.262778794	-0.336471166	10.554560759
1	10.342022032	-0.328924121	10.739776756
1	8.539677769	2.551660157	11.761134874
26	7.277506138	6.531140499	12.729898242
17	7.163757806	4.161837874	12.614581224
7	8.680511968	6.957344866	14.036166908
7	5.945711051	6.601330603	14.156051301
6	8.498099782	7.206209771	15.395135092
6	9.632391375	7.957008662	15.921181569
6	10.552943140	8.069722476	14.884223335
6	9.988539934	7.352722091	13.744559961
6	10.659507832	6.913407856	12.583525838
6	4.589076789	6.345708987	13.969845914
6	4.053550430	5.630468044	15.121411589
6	5.094846184	5.523808376	16.045791435
6	6.236669925	6.215281360	15.467059792
6	7.412290118	6.653209967	16.114654527
6	2.690783261	4.995557483	15.285178453
1	1.892471129	5.702387961	15.011015034
1	2.538024088	4.769100018	16.356191563
6	2.513433754	3.690204510	14.476189800
1	1.529643746	3.237627568	14.699463517
1	3.302198935	2.954895228	14.704616193
1	2.556968605	3.886154428	13.391220466
6	5.041158889	4.705408613	17.316324947
1	3.982820040	4.522282561	17.576295630
1	5.473189841	5.251827882	18.168335621
6	5.756454271	3.342146209	17.171812542
1	5.637083063	2.744439772	18.093610396
1	6.837395342	3.477963917	16.993330681
1	5.347791632	2.763571556	16.326977582
6	9.756648093	8.639837640	17.264202198
1	10.822251011	8.868490737	17.450225504
1	9.444290246	7.969942481	18.079270893
6	8.944217454	9.952223146	17.348521821
1	9.079077990	10.431565479	18.335684192
1	7.866072030	9.759938325	17.206499016
1	9.262475334	10.670387370	16.571579453
6	11.795950156	8.929278717	14.921149627
1	12.662772799	8.402791327	14.494489648
1	12.053723119	9.139922818	15.975103255
6	11.609484339	10.271767333	14.177649758
1	12.522783158	10.889497508	14.256331225
1	10.766031849	10.848687173	14.597657596
1	11.402269980	10.103791612	13.106179924
6	7.521970328	6.537926865	17.602166010
6	8.415039893	5.605835090	18.169502953
1	9.023241495	4.979321025	17.505230018
6	8.522395130	5.458865231	19.564923251
6	7.720761328	6.275276630	20.387621117
1	7.788205452	6.163338082	21.478750033
6	6.826938207	7.223011540	19.851486226
6	6.732250645	7.338303096	18.451221883
1	6.023644731	8.049483144	18.008333312
6	9.485407435	4.456758284	20.170271340
1	9.064968627	3.988753477	21.078382292
1	10.436481036	4.941235778	20.465149451
1	9.733118035	3.652606225	19.456324350
6	5.990359943	8.100946542	20.761116269
1	5.018272973	8.351077636	20.300883393
1	6.503786480	9.057108075	20.979087906
1	5.793322736	7.608075254	21.729574282
6	12.147955082	7.037013594	12.496915848
6	12.982027654	6.242692797	13.309641515
1	12.523527169	5.545656973	14.022010214
6	14.383159886	6.324445087	13.211781416
6	14.937600374	7.243781315	12.299016614
1	16.030762788	7.320972269	12.217570149
6	0.350277910	7.954080424	13.805826911
6	1.755341647	7.864331284	13.757644906
1	2.366036510	8.520728625	14.390557592
6	15.276129549	5.425989121	14.044635050
1	14.735941220	5.012050460	14.913740695
1	16.164179751	5.967579615	14.418897630
1	15.645527772	4.568833581	13.448891243
6	-0.331080191	8.982287570	14.687759242
1	-1.340340992	8.651213331	14.990174910
1	-0.447576139	9.949582511	14.160945028
1	0.252696766	9.179805990	15.604395461
8	5.272735923	1.787156638	13.879080066
6	5.907928310	0.688064118	14.400552731
6	7.310351798	0.584702362	14.490863589
1	7.944650430	1.391896487	14.105513850
6	7.884051343	-0.572838630	15.037279957
1	8.976560014	-0.650714998	15.093024388
6	7.077994317	-1.625454392	15.499141396
1	7.533558020	-2.527474625	15.923423202
6	5.680120247	-1.513931059	15.401187830
1	5.037710416	-2.330712800	15.753112501
6	5.091578204	-0.365488945	14.856698992
1	4.003716377	-0.264414060	14.772499308
1	5.933658864	2.492700101	13.658572590

^{4}2{BP86}$_{optBS1}$

7	5.915141221	6.652517469	11.337060120
7	8.639893439	6.265985013	11.318347249
6	6.172157061	7.042824810	10.006742130
6	5.031016192	7.800397232	9.500513672
6	4.024759782	7.727345646	10.475662290
6	4.559503592	6.942323031	11.584192471
6	3.861280204	6.366167281	12.674804285
6	9.971608836	5.878580292	11.573304146
6	10.485378147	5.135663388	10.424823626
6	9.510096215	5.210759105	9.418489413
6	8.394847052	5.983704998	9.960461376
6	7.283451111	6.539883705	9.286995320
6	11.741267490	4.296078231	10.353815967
1	12.598775969	4.823532519	10.799499467
1	12.001820258	4.125830997	9.292156782
6	11.577170094	2.919706738	11.055634600
1	12.507771053	2.329835951	10.965990335
1	10.752880805	2.338726723	10.607028501
1	11.350845646	3.046975252	12.128234410
6	9.574752377	4.483424626	8.094953255
1	10.627038362	4.212173337	7.888010524
1	9.258744747	5.139865473	7.268449039
6	8.711603279	3.193501689	8.069313433
1	8.842281692	2.665965696	7.107091081
1	7.640444297	3.430160298	8.184324935
1	8.987929165	2.503188541	8.884375041
6	4.967707112	8.641726831	8.245570846
1	3.910661896	8.879504881	8.023270727
1	5.346547161	8.082470123	7.375874463
6	5.762047675	9.969458993	8.372687331
1	5.666193025	10.567900605	7.448289708
1	6.834655520	9.770222007	8.543197724
1	5.391084123	10.576350492	9.218113628
6	2.705082358	8.463711479	10.420512924
1	1.879668744	7.815889954	10.754682507
1	2.482772645	8.727260428	9.369492227
6	2.702540810	9.762165911	11.271168606
1	1.728940671	10.278903665	11.187012875
1	3.493373020	10.458038357	10.937932619
1	2.879568508	9.533993860	12.336989102
6	7.254678983	6.566483464	7.786381117
6	6.342689426	5.747115098	7.080103871
1	5.669521037	5.087574273	7.638102218
6	6.310064158	5.749102675	5.667012113
6	7.200519811	6.598447370	4.971697888
1	7.186001247	6.603971374	3.874560154
6	8.116763134	7.434076380	5.652504754
6	8.139448983	7.401520236	7.063889794
1	8.852887208	8.026772589	7.612036783
6	5.327156698	4.867285170	4.917125112
1	5.755541688	4.503466607	3.966256183
1	4.401768190	5.422873151	4.667197129
1	5.032174991	3.992670469	5.521428732
6	9.045012257	8.352682784	4.877729341
1	9.872491460	8.716581987	5.510208624
1	8.504210088	9.239933635	4.495225184
1	9.484184661	7.838626825	4.003755995
6	2.365521342	6.270272392	12.609085475
6	1.754565976	5.389154259	11.684796925
1	2.384170854	4.773907865	11.033592818
6	0.349093916	5.270398821	11.616311225
6	-0.439899103	6.067896361	12.478795791
1	-1.533028276	5.985626797	12.431818949
6	14.381090239	6.947235802	11.963740385
6	12.971375862	7.036910628	11.910782567
1	12.494958161	7.714642193	11.193848764
6	-0.303180488	4.284309884	10.663593153
1	0.409036516	3.931840704	9.899111036
1	-1.171118061	4.733298550	10.147328861
1	-0.672290771	3.392510122	11.205513131
6	15.247673614	7.811200488	11.063912995
1	16.214063434	7.323213596	10.846292945
1	15.471810107	8.787180265	11.537168069

1	14.746231081	8.021817699	10.102734935
8	6.537625687	1.409858685	10.579907095
6	5.486134685	1.754115197	9.744907874
6	4.879017064	3.034290051	9.752697197
1	5.231365912	3.797867084	10.453690750
6	3.829151338	3.308593212	8.852744651
1	3.371012132	4.304618415	8.855046934
6	3.372466288	2.324238847	7.951596784
1	2.554297384	2.543140407	7.258026146
6	3.980015810	1.049537161	7.960043866
1	3.632281831	0.273728081	7.269406582
6	5.030090892	0.761648285	8.846992892
1	5.513837737	-0.218402023	8.866309467
1	6.782190816	2.161502310	11.198868738
26	7.261743384	6.215577779	12.691816114
17	7.252694833	3.692242148	12.683615864
7	8.610437366	6.636066166	14.050350929
7	5.882499985	6.261638481	14.064408928
6	8.352597233	7.022115285	15.382388887
6	9.495014894	7.776184391	15.892123942
6	10.502298421	7.704067051	14.917725410
6	9.966920005	6.925416072	13.804643621
6	10.663548394	6.356601048	12.708228760
6	4.550136534	5.879206248	13.806859958
6	4.034360779	5.129422115	14.949725762
6	5.010811323	5.193372284	15.955770527
6	6.126443344	5.969437482	15.419752292
6	7.237686383	6.520244603	16.098193878
6	2.775292149	4.294287904	15.015516969
1	1.922242641	4.823864197	14.564168043
1	2.508204756	4.126945474	16.076032653
6	2.939004623	2.916362377	14.316952187
1	2.005804449	2.329912687	14.401406497
1	3.758410603	2.333289340	14.771819669
1	3.172718392	3.041389022	13.245674689
6	4.948425444	4.452133563	17.271757724
1	3.899920590	4.158243099	17.465662065
1	5.243504455	5.106612200	18.107850291
6	5.836490107	3.178897563	17.293167983
1	5.702094449	2.636072368	18.246422944
1	6.904429718	3.437579698	17.197672323
1	5.586537082	2.494139731	16.464927524
6	9.557072049	8.617716939	17.147408524
1	10.612972435	8.865867771	17.363686386
1	9.188031412	8.055914718	18.020270463
6	8.749988590	9.937915961	17.023178503
1	8.844705559	10.537561943	17.946782901
1	7.678632321	9.728496280	16.858091913
1	9.111289863	10.547648418	16.175693836
6	11.824079400	8.436223832	14.977039202
1	12.646961231	7.788287255	14.636708948
1	12.048523507	8.691059806	16.029785036
6	11.829434650	9.740984820	14.136214103
1	12.804804559	10.253979824	14.222436580
1	11.041362903	10.436753008	14.476062281
1	11.649846715	9.521228578	13.069005709
6	7.252786775	6.553253957	17.598423784
6	8.172130784	5.756126250	18.318910499
1	8.859966301	5.102864210	17.771329887
6	8.197774849	5.773747810	19.732076279
6	7.287737470	6.612952402	20.413695703
1	7.296322608	6.630222911	21.510382413
6	6.360191062	7.424531055	19.718935637
6	6.348484091	7.379282034	18.307643588
1	5.628933213	7.987872485	17.748727197
6	9.197445654	4.922806933	20.495960136
1	8.781213131	4.577650301	21.459528347
1	10.117816842	5.496534717	20.724098559
1	9.499272911	4.036769795	19.910915942
6	5.410179742	8.333190112	20.479095505
1	4.579661617	8.675106531	19.838126204
1	5.932055222	9.234287959	20.855736370
1	4.975598352	7.819693126	21.355881877
6	12.160405775	6.257892455	12.767695043
6	12.773433179	5.369340790	13.683708603
1	12.145056843	4.751399251	14.333714731
6	14.178912149	5.245951398	13.744095232
6	14.965606288	6.046850851	12.882917083
1	16.058690581	5.960786729	12.923606642
6	0.142654796	6.960497444	13.406809147
6	1.552326487	7.045546486	13.467212573
1	2.026862887	7.717665782	14.190530401
6	14.833187122	4.250286725	14.685542918
1	14.129602672	3.909890669	15.464390184
1	15.718578970	4.685760582	15.184691548
1	15.176868008	3.351577688	14.137089237
6	-0.726134416	7.820968048	14.307914366
1	-1.687495109	7.326493862	14.532549857
1	-0.960502289	8.792983406	13.832134216
1	-0.221757489	8.039064010	15.265462265
8	7.979676293	1.422848696	14.798315440
6	9.028877821	1.773255400	15.634020636
6	9.637837318	3.052535860	15.616377483
1	9.288069287	3.810868600	14.908226929
6	10.685552048	3.333336540	16.516744124
1	11.145458706	4.328515070	16.506039913
6	11.137711230	2.356591060	17.428716994
1	11.954465446	2.580378891	18.122282910
6	10.527750089	1.082986814	17.431101418
1	10.871634204	0.313307914	18.130655371
6	9.480048881	0.788511405	16.543420612
1	8.994222816	-0.190676176	16.532459548
1	7.738650463	2.168711792	14.170799420

^{4}2{BP86}$_{optBS2}$

7	5.904736326	6.725195475	11.318631635
7	8.660095493	6.399073613	11.308666082
6	6.148005054	7.080042268	9.990783542
6	4.982695725	7.762136226	9.444237766
6	3.981989437	7.684018788	10.413275122
6	4.553071133	6.975119135	11.549523774
6	3.871477046	6.431018190	12.666934289
6	9.977174944	6.008360280	11.562910328
6	10.511871769	5.292153556	10.412418468
6	9.545697492	5.368652098	9.409135759
6	8.421467671	6.110035756	9.964735616
6	7.286872973	6.615374450	9.290871559
6	11.778997008	4.471916864	10.322645042
1	12.634045918	5.004123233	10.764767840
1	12.030508253	4.320800968	9.256742761
6	11.640932401	3.088441281	11.002313820
1	12.580380273	2.515054658	10.898571865
1	10.824936436	2.497899799	10.550796165
1	11.419629639	3.192431176	12.078372745
6	9.642385768	4.666587070	8.073830624
1	10.709196875	4.487755083	7.844842639
1	9.260473000	5.302320061	7.261662416
6	8.894774262	3.312795648	8.049622016
1	9.025590834	2.820231903	7.068849066
1	7.813302690	3.448996155	8.220721141
1	9.269150610	2.628970164	8.830864504
6	4.884244580	8.554361475	8.160942610
1	3.818074859	8.733614223	7.931488317
1	5.288505931	7.987070560	7.309339203
6	5.610739022	9.916595637	8.249354662
1	5.490772935	10.482785248	7.307507837
1	6.691386498	9.776943030	8.428700001
1	5.209523527	10.532319111	9.074490084
6	2.631748120	8.356709119	10.319981418
1	1.832184141	7.688228182	10.672751508
1	2.408559475	8.565331283	9.257775061
6	2.569805383	9.683251718	11.111960096
1	1.576014659	10.155316730	11.003634817
1	3.331321170	10.399201604	10.753735560
1	2.750975995	9.510897721	12.187566823
6	7.257611414	6.615144993	7.794871766
6	6.421408431	5.712615766	7.106690059
1	5.801857115	5.011590900	7.680198180
6	6.387446317	5.685348991	5.699464612
6	7.200959399	6.592004799	4.991312138
1	7.185994657	6.577197159	3.892396820
6	8.038415015	7.513383310	5.651578203
6	8.064352904	7.506593556	7.058963797
1	8.725032790	8.198914026	7.596206662
6	5.482407518	4.715097073	4.966148231
1	5.985532151	4.279299315	4.084138118
1	4.568898971	5.221896017	4.598890744
1	5.161108065	3.889002081	5.622844811
6	8.882493303	8.493265323	4.860772127
1	9.685394941	8.927947864	5.480833594
1	8.270069484	9.331915104	4.476863919
1	9.351715010	8.009143250	3.985172601
6	2.386406236	6.268413822	12.583504583
6	1.847251949	5.295968257	11.714067080
1	2.526462741	4.671841358	11.120906630
6	0.457660276	5.097069732	11.624738876
6	-0.387866399	5.909506400	12.408206413
1	-1.475490869	5.764269148	12.346717867
6	14.417058029	6.902276393	12.126187248
6	13.020570864	7.066534423	12.036514493
1	12.601600128	7.814931947	11.351925485
6	-0.117059261	4.018037205	10.728901634
1	0.662472996	3.562197284	10.095582557
1	-0.907689252	4.418472232	10.067848395
1	-0.575918743	3.207363944	11.325852121
6	15.353863126	7.777579609	11.316912086
1	16.305120884	7.260260488	11.100329610
1	15.604487993	8.708231127	11.861491928
1	14.900385278	8.075790610	10.355022182
8	6.580173202	1.407516093	10.799162431
6	5.510364513	1.671741940	10.001002214
6	4.942139310	2.961264364	9.874763892
1	5.359224615	3.793457829	10.454363680
6	3.851938365	3.159008472	9.011194130
1	3.428415535	4.167034706	8.910988107
6	3.309361774	2.090808893	8.275197502
1	2.459453479	2.252854228	7.602360985
6	3.876121023	0.811010530	8.413252925
1	3.463644018	-0.034369730	7.848784859
6	4.966668072	0.596721608	9.264761566
1	5.417184629	-0.395209150	9.378404525
1	6.824486700	2.230801586	11.301894683
26	7.266237240	6.330400366	12.691107300
17	7.260892108	3.859227057	12.689984488
7	8.625669315	6.728561161	14.063749679
7	5.873761011	6.396872291	14.075207064
6	8.378718534	7.088163739	15.389210673
6	9.538265704	7.777621102	15.934187660
6	10.543548158	7.694577558	14.970438141
6	9.977422894	6.979986681	13.835445010
6	10.662341389	6.436558896	12.719695969
6	4.558205721	6.002943443	13.822066378
6	4.027180957	5.282927532	14.971628237
6	4.993313533	5.362811406	15.974161059
6	6.112905295	6.109112009	15.418656146
6	7.244065639	6.619073832	16.091273277
6	2.764132847	4.456725407	15.059779724
1	1.908062491	4.982817132	14.612266879
1	2.509269055	4.307570664	16.125203986
6	2.913830427	3.072156910	14.384430479
1	1.976956110	2.493696023	14.483736866
1	3.730340804	2.487363468	14.842543376
1	3.141382167	3.175172142	13.309470097
6	4.903748846	4.659259181	17.308862813
1	3.839126757	4.465481139	17.535613500
1	5.275418279	5.300236049	18.121889012
6	5.670533533	3.316074856	17.334441487
1	5.538662139	2.818322709	18.312462090
1	6.751117581	3.468253850	17.173200062
1	5.313122713	2.629538672	16.547711990
6	9.633090518	8.579410170	17.211757052
1	10.698496872	8.767446189	17.437960630
1	9.234433533	8.016034674	18.068385232
6	8.898668927	9.936784756	17.114347863
1	9.016580756	10.510280468	18.052061762
1	7.818782554	9.790151106	16.937060498
1	9.295958434	10.548744664	16.284530037
6	11.895406974	8.363725043	15.068865466
1	12.693810210	7.692957562	14.717709441
1	12.115995925	8.569623499	16.132176671
6	11.965661597	9.691453173	14.279859589
1	12.961005899	10.159121428	14.392848148
1	11.205934277	10.409904987	14.636750337
1	11.787863908	9.521861120	13.203216535
6	7.276757733	6.619488169	17.586905813
6	8.121919969	5.724556701	18.273247731
1	8.744240484	5.027010011	17.699007192
6	8.163027776	5.701170423	19.680445605
6	7.347292899	6.604368096	20.389875908
1	7.368110396	6.592979266	21.488715677
6	6.501189876	7.518445553	19.731106071
6	6.468215989	7.507210774	18.324165259
1	5.801848910	8.194778422	17.788459616
6	9.079447817	4.740178678	20.411980079
1	8.593140160	4.322481563	21.311887997
1	10.002703945	5.249505563	20.750620858
1	9.384060163	3.901267086	19.763618359
6	5.656796711	8.496223743	20.524001655
1	4.849446104	8.926558357	19.906992456
1	6.267710592	9.337871578	20.903665062
1	5.193641582	8.011890385	21.402709858
6	12.147950729	6.274394531	12.807290545
6	12.684222659	5.299712093	13.676367377
1	12.002848297	4.674281183	14.265511101
6	14.073423078	5.099940109	13.770340511
6	14.922046473	5.913997563	12.992317415
1	16.009314112	5.768297350	13.057754860
6	0.120658917	6.895583637	13.275332376
6	1.517645646	7.059062514	13.359937030
1	1.939892160	7.805492794	14.044658253
6	14.645268864	4.017204638	14.663570922
1	13.867491157	3.571590632	15.306189323
1	15.447334953	4.410516899	15.315081465
1	15.088577370	3.199484966	14.064078523
6	-0.812217533	7.769428923	14.090780992
1	-1.756442162	7.246311701	14.323886365
1	-1.077564193	8.694101682	13.542918740
1	-0.348078376	8.078051207	15.044151444
8	7.884609682	1.397677422	14.588532089
6	8.964947907	1.638064179	15.379773468
6	9.569382409	2.911823596	15.491813554
1	9.172989275	3.749655508	14.906259723
6	10.668093558	3.086974334	16.348849713
1	11.119429672	4.083759312	16.439205630
6	11.183801334	2.010969797	17.092056970
1	12.041328661	2.155026176	17.759396349
6	10.581465141	0.745765560	16.967857106
1	10.973153847	-0.105922732	17.537942300
6	9.481296259	0.554451457	16.122922979
1	9.002412069	-0.425411106	16.019894379
1	7.660664490	2.223619218	14.080661162

^{6}2{BP86}$_{optBS1}$

7	5.870178888	6.703201564	11.297817965
7	8.716100388	6.468887818	11.257762358
6	6.132025029	7.065752055	9.963436658
6	4.967703599	7.778996344	9.431745441
6	3.966437149	7.723949412	10.413701624
6	4.520994230	6.991140605	11.554801224
6	3.831562573	6.471701532	12.691532385
6	10.029853892	6.060533022	11.535283518
6	10.539325099	5.280886730	10.400509266
6	9.557418396	5.325465319	9.399979022
6	8.445025820	6.124873655	9.926181593
6	7.282610638	6.604480924	9.263929545
6	11.785739723	4.424787721	10.347139007
1	12.649554152	4.953158815	10.779330607
1	12.042727097	4.229173949	9.288799336
6	11.611805265	3.065026173	11.077694245
1	12.537331124	2.465721020	11.000014851
1	10.782710007	2.481004328	10.642032202
1	11.388111934	3.215931493	12.147758926
6	9.597261441	4.530615536	8.113823635
1	10.645166193	4.248548882	7.898934402
1	9.263062931	5.142289017	7.260680734
6	8.735809781	3.239729986	8.173928275
1	8.835976958	2.667926760	7.233453929
1	7.667996006	3.479851905	8.311697619
1	9.040589375	2.587757942	9.010370046
6	4.881315340	8.575646512	8.148623779
1	3.818765746	8.783381063	7.922345053
1	5.268622444	7.996646179	7.295652161
6	5.646992914	9.923807166	8.228997854
1	5.538559746	10.487388799	7.284186784
1	6.723371339	9.753572664	8.406576665
1	5.262471152	10.551534408	9.052938407

6	2.634785590	8.436949236	10.336864534
1	1.817956327	7.780116131	10.675058340
1	2.415326901	8.680206769	9.280531849
6	2.603168108	9.748216360	11.166822457
1	1.620225762	10.244737367	11.069993796
1	3.382305581	10.453307827	10.825892663
1	2.779952408	9.540903378	12.236888802
6	7.245672544	6.575412158	7.764508975
6	6.341652953	5.713798386	7.098228910
1	5.681001582	5.070195169	7.688564333
6	6.299743501	5.651256300	5.688573333
6	7.172195041	6.485200101	4.950550470
1	7.149673750	6.445730207	3.854097632
6	8.079888099	7.361997194	5.588367984
6	8.115827757	7.389014868	7.001231243
1	8.824265380	8.046705503	7.516798125
6	5.335368078	4.713841875	4.982222774
1	5.845499904	4.130471596	4.193575688
1	4.516772100	5.275525109	4.492034266
1	4.877716353	4.001829261	5.689884856
6	8.982860119	8.267303180	4.768609614
1	9.862747688	8.593002289	5.349563155
1	8.446287675	9.179487844	4.442190878
1	9.344109665	7.758554922	3.857083525
6	2.342830139	6.306225670	12.580803192
6	1.824384032	5.357425224	11.664326495
1	2.518384962	4.759223198	11.065231073
6	0.435234809	5.150231115	11.539073726
6	-0.436704227	5.926590119	12.340804058
1	-1.520001285	5.775342590	12.254119265
6	14.430971535	6.992579921	11.993926386
6	13.025520567	7.118430209	11.926604414
1	12.572088694	7.799479038	11.198302983
6	-0.115183060	4.112558536	10.576064391
1	0.696546975	3.571686867	10.062105131
1	-0.756455956	4.578678572	9.804183188
1	-0.735867485	3.364718625	11.104027005
6	15.331449787	7.811359272	11.085279970
1	16.109613696	7.181976310	10.616286906
1	15.854213159	8.608399896	11.648612318
1	14.755759703	8.297203630	10.278945153
8	6.454343013	1.423253005	10.660319101
6	5.392972624	1.743901023	9.827415105
6	4.832849190	3.042438219	9.750972249
1	5.231146889	3.846284614	10.379200857
6	3.767404839	3.286201085	8.859932716
1	3.344418580	4.295496569	8.797283195
6	3.250344436	2.252355893	8.051362243
1	2.421279428	2.447773543	7.363837361
6	3.812832768	0.960155705	8.142962908
1	3.418231882	0.146974833	7.524145161
6	4.877176281	0.702719855	9.021698578
1	5.326367106	-0.290322340	9.105440242
1	6.733470311	2.202322655	11.225528182
26	7.266001904	6.176251381	12.702023222
17	7.337096229	3.743046265	12.646908426
7	8.658905424	6.697940667	14.109299396
7	5.815108789	6.438447774	14.147486172
6	8.394843217	7.059381459	15.441142128
6	9.548349379	7.793767804	15.968416965
6	10.548246123	7.753014234	14.984301241
6	10.005096058	7.004816172	13.848988541
6	10.695722249	6.492924736	12.712389200
6	4.492701016	6.050121278	13.874816634
6	3.970295737	5.298680672	15.024833453
6	4.954361939	5.338556261	16.023068820
6	6.082550956	6.110353873	15.485852029
6	7.249316585	6.584143712	16.144603590
6	2.701082270	4.478564051	15.105210032
1	1.852218711	5.015012098	14.654745238
1	2.438611606	4.325882031	16.169284102
6	2.836471659	3.090291984	14.422524406
1	1.894858459	2.520148646	14.521874836
1	3.649356643	2.498880301	14.878428750
1	3.060704990	3.198337322	13.347467368
6	4.893691332	4.567134976	17.322872059
1	3.839684377	4.307147398	17.535456649
1	5.232480804	5.188167341	18.167347637
6	5.732390851	3.260365336	17.293550859
1	5.605762540	2.703859241	18.240083619
1	6.806603316	3.479422016	17.171723800
1	5.431512051	2.601534019	16.461094390
6	9.622536908	8.598259781	17.246949403
1	10.680396051	8.839304053	17.461580280
1	9.262042687	8.009851560	18.105107465
6	8.814719647	9.921838455	17.170816313
1	8.914693172	10.490938912	18.113127748
1	7.742299581	9.718392482	17.004489025
1	9.171444111	10.559015150	16.341763030
6	11.863013494	8.497780038	15.048557439
1	12.691504571	7.863859015	14.695602002
1	12.090407201	8.741457748	16.103230920
6	11.849887874	9.812683557	14.223415339
1	12.819413988	10.336089936	14.312655834
1	11.054514885	10.494462761	14.574307846
1	11.668902586	9.603635379	13.154312670
6	7.315944882	6.538389939	17.644403342
6	8.238983349	5.673220267	18.280355620
1	8.882144454	5.032151974	17.668193704
6	8.318727039	5.603018408	19.688888585
6	7.467623493	6.431769148	20.456189397
1	7.519955547	6.384625663	21.551176715
6	6.542548075	7.312161548	19.848641806
6	6.469405929	7.348077046	18.437742275
1	5.748636975	8.010959708	17.946287889
6	9.303780641	4.664132184	20.362835364
1	8.835900380	4.131153363	21.210173291
1	10.173160113	5.218208794	20.767598073
1	9.687551919	3.910551636	19.655027898
6	5.659153626	8.208824943	20.698467138
1	4.790156351	8.577651884	20.127080774
1	6.217111631	9.093944388	21.061495988
1	5.280971952	7.675363382	21.589292997
6	12.188038209	6.370432423	12.787174730
6	12.771541554	5.478710111	13.718972617
1	12.121027238	4.884419300	14.369244128
6	14.172246029	5.320170096	13.795345740
6	14.985601823	6.088672514	12.930012983
1	16.075867621	5.974516082	12.980167819
6	0.050566645	6.884855319	13.256708800
6	1.450158459	7.061468275	13.372456422
1	1.851655658	7.787745938	14.088021765
6	14.792316819	4.338741753	14.774847617
1	14.019376986	3.816507084	15.363300822
1	15.472837354	4.849158256	15.482501541
1	15.388569943	3.569734495	14.248619887
6	-0.902438993	7.714701103	14.098992926
1	-1.926033492	7.304161599	14.069819655
1	-0.952099928	8.760519833	13.739125020
1	-0.580171237	7.751030924	15.155570574
8	7.831201644	1.447818719	14.804824483
6	8.925784788	1.692505231	15.622057652
6	9.626539334	2.923266968	15.628836779
1	9.317660745	3.729738662	14.955676088
6	10.715717905	3.094870901	16.507118516
1	11.248568087	4.052965985	16.517034072
6	11.116875541	2.056501899	17.373020869
1	11.966829153	2.194719409	18.048848022
6	10.413221500	0.832265081	17.351938241
1	10.716782949	0.015764904	18.015932707
6	9.324062619	0.646557774	16.485359787
1	8.766817476	-0.293370896	16.455144197
1	7.646438668	2.219232277	14.192771577

^{6}2{BP86}$_{optBS2}$

7	5.634133337	6.864229884	11.272026680
7	8.472730772	6.543426592	11.218847896
6	5.909722742	7.256612584	9.971163877
6	4.809646009	8.074445728	9.464976477
6	3.822635059	8.070899902	10.450664272
6	4.331235299	7.250195952	11.549220209
6	3.621980558	6.770151656	12.686039188
6	9.777817028	6.143359284	11.459697678
6	10.303996587	5.442482681	10.286720990
6	9.315753294	5.511489012	9.305408174
6	8.200454378	6.259273217	9.889030983
6	7.041746444	6.777657878	9.254860943
6	11.590472315	4.656297148	10.170166754
1	12.449564906	5.255337519	10.511592840
1	11.776777071	4.439378098	9.102508529
6	11.563673587	3.319354264	10.947742336
1	12.501602358	2.759994239	10.773706562
1	10.715224128	2.684783316	10.641411536
1	11.471980123	3.492598199	12.033940848
6	9.367481225	4.792143760	7.976748838
1	10.420440979	4.546186514	7.747402512
1	9.022466528	5.440935495	7.157537631
6	8.539974838	3.485405357	7.972080015
1	8.666059029	2.953206477	7.011432554
1	7.464204198	3.698254136	8.101769161
1	8.848108263	2.810777057	8.788507118
6	4.781951672	8.922862981	8.213994799
1	3.739599911	9.230365644	8.011016092
1	5.105437194	8.345447019	7.334582724
6	5.659946663	10.190905201	8.334145396
1	5.594003955	10.798455155	7.412575990
1	6.719996650	9.925090200	8.493091744
1	5.340551822	10.820201865	9.184371812
6	2.573959501	8.922894606	10.423906468
1	1.695816127	8.356384569	10.768772319
1	2.359017525	9.212642340	9.378866718
6	2.713806594	10.205100792	11.277510091
1	1.794294431	10.816076701	11.215265790
1	3.562229579	10.824459816	10.934501411
1	2.887974559	9.955658944	12.339180239
6	6.989206203	6.805353006	7.761694492
6	6.055682228	5.999964896	7.073378007
1	5.371901614	5.365182595	7.650182395
6	6.008797010	5.986709795	5.667904687
6	6.907088675	6.810350161	4.958183390
1	6.885001534	6.801100342	3.859603659
6	7.837647031	7.637584872	5.615958194
6	7.872637429	7.617448753	7.023359719
1	8.605738309	8.233329053	7.559708312
6	5.010232231	5.118447917	4.927746933
1	5.463746579	4.651491117	4.034950349
1	4.143605436	5.712479097	4.577470230
1	4.620342839	4.312100034	5.571900234
6	8.777627184	8.531716306	4.831160004
1	9.758097216	8.627950516	5.331819548
1	8.364674157	9.553856221	4.725512793
1	8.950684679	8.141437604	3.812813910
6	2.127558855	6.792095860	12.640363737
6	1.437495173	6.024021197	11.676449733
1	2.014846372	5.411012949	10.973078977
6	0.032426541	6.031217833	11.611697846
6	-0.677713075	6.848342114	12.515973855
1	-1.774650158	6.871072067	12.466840215
6	14.098692579	7.345132798	11.862176605
6	12.694046629	7.351715142	11.842886731
1	12.159771980	8.049538755	11.185967704
6	-0.704106090	5.166114920	10.608157716
1	-0.073161361	4.934758768	9.732276453
1	-1.624372157	5.657973640	10.246535870
1	-1.006400363	4.200016964	11.057940013
6	14.897166651	8.313345275	11.012122822
1	15.736991701	7.806975637	10.502055422
1	15.334235752	9.122310664	11.628697771
1	14.267077943	8.788450955	10.240673401
8	8.506831062	1.560491130	11.061370909
6	7.747496529	0.555648007	10.513131164
6	6.340032877	0.606733617	10.452151156
1	5.811542898	1.471788437	10.871106789
6	5.631706367	-0.452796599	9.863007551
1	4.536758078	-0.404221320	9.821698795
6	6.306271964	-1.564348521	9.331506502
1	5.746635984	-2.387720340	8.872962805
6	7.710675648	-1.606144467	9.401000542
1	8.254697597	-2.467384769	8.993492897
6	8.432329090	-0.557100685	9.986050868
1	9.525266779	-0.579897746	10.044589085
1	7.922285691	2.292427502	11.401412992
26	7.019264384	6.210208644	12.657976616
17	6.901906869	3.816508815	12.582310362
7	8.451601723	6.655132084	14.074393853
7	5.591630572	6.562327985	14.123358360
6	8.197945391	6.988114076	15.396027637
6	9.350322612	7.697060424	15.948961555
6	10.343123812	7.674609986	14.969149902
6	9.781533124	6.967128080	13.820906663
6	10.454606008	6.525440103	12.648580609
6	4.266075822	6.262877776	13.847488526
6	3.688090510	5.517656177	14.966583928
6	4.660206699	5.473787640	15.964446165
6	5.833942560	6.171710190	15.435001843
6	7.033747054	6.547118860	16.092450126
6	2.378243130	4.764634809	15.017139819
1	1.532597488	5.408851760	14.728492686
1	2.190767282	4.455834554	16.061618820
6	2.377277564	3.504405064	14.120493083
1	1.424084483	2.955459789	14.229475388
1	3.203400474	2.822674780	14.387896854
1	2.497160152	3.773273068	13.057248892
6	4.513463900	4.714279610	17.264231252
1	3.459101847	4.777662145	17.598043808
1	5.111013494	5.181927937	18.058515446
6	4.923273802	3.227785298	17.139717647
1	4.840265551	2.724131954	18.120392613
1	5.963817489	3.137420618	16.782687946
1	4.275735812	2.689191355	16.424847460
6	9.428414852	8.487674158	17.235424044
1	10.485807638	8.734099089	17.441305889
1	9.081986410	7.892981618	18.093878499
6	8.615519294	9.802020168	17.172116994
1	8.720576142	10.369316526	18.115052823
1	7.542067303	9.596774890	17.015143077
1	8.958858689	10.446123391	16.342935193
6	11.661684405	8.408242608	15.065898540
1	12.484081003	7.804051929	14.656132428
1	11.904726587	8.568385829	16.132216857
6	11.637895096	9.777969811	14.348836885
1	12.610211395	10.292092151	14.461410943
1	10.851873379	10.434573583	14.762902079
1	11.439029876	9.655042045	13.269938321
6	7.129192681	6.461320082	17.581843775
6	8.055254878	5.573262920	18.171416565
1	8.653334431	4.927055235	17.519120970
6	8.189812740	5.496134833	19.570838393
6	7.385481411	6.330003115	20.374539116
1	7.483364553	6.276225462	21.467731608
6	6.456211092	7.228426119	19.812985506
6	6.332780688	7.277372692	18.411497692
1	5.607661966	7.960955086	17.951375496
6	9.197790591	4.557884088	20.205791228
1	8.759121530	4.005122999	21.056658043
1	10.068912889	5.116178688	20.600086939
1	9.579529759	3.822268490	19.477634561
6	5.623115617	8.133146136	20.699809974
1	4.741527414	8.524984271	20.163171857
1	6.210155220	9.004072993	21.050877341
1	5.265739276	7.600856486	21.599736137
6	11.949166779	6.478580682	12.664937954
6	12.631310957	5.577447837	13.505232362
1	12.052358079	4.902565034	14.147873201
6	14.039818708	5.530983939	13.527610267
6	14.753029125	6.424940394	12.707661638
1	15.851580900	6.405512385	12.726454656
6	-0.018346248	7.634431921	13.481786273
6	1.388085754	7.586011544	13.542327916
1	1.924046647	8.180277749	14.293215176
6	14.763673937	4.527202073	14.402471821
1	14.262246323	4.404853503	15.378653687
1	15.808308854	4.831037849	14.588496702
1	14.788380332	3.528453881	13.926237732
6	-0.800303162	8.532398504	14.420766107
1	-1.843838103	8.189999232	14.533078607
1	-0.834265245	9.573286093	14.045065482
1	-0.341602587	8.566090883	15.425342447
8	8.026655440	3.255770672	15.499207084
6	8.849214699	2.171125658	15.652120404
6	9.378607599	1.443268415	14.566317557
1	9.118390396	1.714248372	13.536514268
6	10.230415625	0.355807120	14.814300265
1	10.632505490	-0.206464277	13.963217879
6	10.564461452	-0.017546449	16.126176908
1	11.230559825	-0.868329901	16.309139168
6	10.023425000	0.707061843	17.202287858

1	10.262959876	0.419851127	18.234393740
6	9.168022334	1.792367095	16.972808263
1	8.718333054	2.349961939	17.801791144
1	7.783332170	3.380990303	14.542108289

^{2}4{BP86}$_{optBS1}$

26	5.530647676	10.223990390	17.861361071
17	7.193514054	8.743390673	18.679056898
7	5.753077887	11.452525880	19.350288447
7	3.879496937	9.560763990	18.682510640
7	5.214874075	9.036000265	16.337958291
7	6.587361567	11.410839645	16.729527488
6	6.882461824	12.280283704	19.504448515
6	7.259629269	12.332940459	20.914123023
6	6.280411628	11.622570060	21.625224054
6	5.297613034	11.162295994	20.650930543
6	3.988555926	10.677604709	20.871125868
6	3.245614480	10.084323404	19.825886682
6	1.828488154	9.714622504	19.808938430
6	1.654609062	8.824682924	18.744330923
6	2.961891931	8.664497792	18.106395761
6	3.382019985	7.655206397	17.206254230
6	4.586960380	7.779702310	16.481079582
6	5.288109893	6.788758611	15.671040668
6	6.262887090	7.478837715	14.935862449
6	6.159266952	8.889757845	15.306125356
6	6.763572105	10.015916160	14.699266788
6	6.780283459	11.278317577	15.339627543
6	7.168125897	12.576316110	14.780249522
6	7.347313211	13.450428584	15.857340481
6	7.069057769	12.690430193	17.078838394
6	7.384451622	13.030860580	18.415528878
6	8.520573948	12.902339691	21.525206845
1	8.784766197	13.861661267	21.056856808
1	8.333726228	13.123897352	22.593791769
6	6.353747041	11.273334356	23.095373046
1	7.079172492	11.946883690	23.588994719
1	5.386059260	11.444282482	23.594761326
6	6.791945627	9.803807609	23.341962453
1	6.856994717	9.598076434	24.426234776
1	6.071002096	9.095269154	22.898874668
1	7.777964138	9.599221578	22.890171133
6	0.718862184	10.303506039	20.652074236
1	-0.186016973	9.676416515	20.545598876
1	0.980326613	10.285921576	21.722696625
6	0.366305282	11.759683092	20.247040061
1	-0.467568714	12.143119650	20.862986625
1	1.233677516	12.427781216	20.389475772
1	0.066541346	11.814684196	19.185127400
6	0.328618331	8.284334034	18.256548199
1	0.397584340	7.206013528	18.040136634
1	-0.421590257	8.387797915	19.063138050
6	-0.193010377	9.017231348	16.991260346
1	-1.170589031	8.606601421	16.678246766
1	-0.316902614	10.098625296	17.181535202
1	0.514005151	8.901821373	16.150285681
6	5.135640235	5.283794243	15.708507263
1	4.074253285	4.990178027	15.671727652
1	5.605942942	4.852405389	14.804601953
6	5.790293251	4.642762560	16.962189939
1	5.685528959	3.542982485	16.932541244
1	6.864072664	4.888579553	17.022275777
1	5.314243721	5.009560626	17.887595833
6	7.311639305	6.822195073	14.065828952
1	6.935751074	5.836776066	13.730507051
1	7.491688944	7.408382580	13.151847026
6	8.662658935	6.615920038	14.804382173
1	9.393906774	6.123301919	14.137728565
1	9.087632005	7.581044781	15.129494921
1	8.534048283	5.987925858	15.703186132
6	7.211187276	12.971728230	13.320292707
1	7.768206426	13.922307309	13.221788766
1	7.765025256	12.228949504	12.724230237
6	5.798553016	13.156988383	12.704733658
1	5.874324287	13.469078252	11.646938904
1	5.225596577	12.213844958	12.744634807
1	5.224232330	13.925770537	13.252421592
6	7.618996065	14.934066300	15.739427351
1	8.388437698	15.254398883	16.459511915
1	8.031494366	15.145704560	14.735025267
6	6.345509198	15.796391723	15.946207012
1	6.581120540	16.869890128	15.829313474
1	5.562631444	15.531400675	15.213026069
1	5.928967295	15.645193218	16.957502580
6	3.378393323	10.798398483	22.235338449
6	3.098923903	12.073515990	22.784717235
1	3.339833829	12.969356738	22.202966679
6	2.522927384	12.204591655	24.065172018
6	2.514598755	9.744805294	24.284674042
6	3.079408803	9.643555807	22.991990719
1	3.294286538	8.657249986	22.566548034
6	2.541801996	6.416961455	17.101548854
6	2.416176063	5.575649209	18.234946839
1	2.943076558	5.846766611	19.155772639
6	1.626705893	4.409382790	18.195147135
6	0.943133732	4.099966612	16.994364435
1	0.318025352	3.199553351	16.951099953
6	1.043886569	4.920264890	15.850244437
6	1.855213781	6.078880518	15.915323896
1	1.949241126	6.729436504	15.038760792
6	7.420282831	9.862847441	13.357133712
6	6.645682491	9.599841410	12.203426142
1	5.557621177	9.509688653	12.299434815
6	7.256947608	9.446881365	10.937230867
6	8.663891267	9.549428653	10.850989532
1	9.148448721	9.421702748	9.875157430
6	9.464458712	9.805424493	11.989392597
6	8.827616850	9.964596599	13.240013015
1	9.429485216	10.152787393	14.135945810
6	8.255991028	14.217874698	18.704147423
6	9.632387129	14.200978808	18.387803578
1	10.066890541	13.312611841	17.916135391
6	9.869118888	16.450036275	19.283178919
6	8.491719843	16.494195189	19.606553404
6	7.697353920	15.365497674	19.317540238
1	6.631472857	15.370394114	19.570742823
1	1.825011421	7.685515435	24.478052722
6	1.503378791	3.504240261	19.408330607
1	1.880012667	2.486979989	19.191458358
1	2.076229833	3.899138223	20.264338515
1	0.449319600	3.398151244	19.727385589
6	0.303815679	4.570169664	14.570991188
1	-0.373011391	3.711732311	14.719890259
1	-0.302419945	5.421070029	14.209825591
1	1.007112815	4.305615171	13.758633529
6	6.417062424	9.166239423	9.702209779
1	7.043185374	9.106904013	8.795308927
1	5.868912114	8.209716607	9.795554720
1	5.661240331	9.957478117	9.539673590
6	10.974003136	9.923001217	11.871175959
1	11.287994600	10.981834956	11.788486426
1	11.480036446	9.507866281	12.759764001
1	11.351651614	9.395011532	10.977945220
6	7.891085959	17.726774454	20.260782160
1	8.096411814	18.638639953	19.669286311
1	6.796986214	17.632855868	20.367480273
1	8.313403236	17.893227668	21.270112297
7	10.315542986	9.244436592	18.537460715
1	9.286955940	9.215956846	18.469035947
6	11.287576424	11.661455439	15.879594514
1	10.794685524	12.310968670	15.146273724
6	10.491017290	10.866446250	16.723202733
1	9.398042075	10.900011035	16.650436256
7	7.276705239	6.024078009	20.694165127
1	7.266269645	6.854245280	20.096663634
1	6.387029473	5.583492285	20.900960246
6	8.443451072	4.279678598	21.949782948
1	7.482623556	3.815895935	22.206252092
6	8.456175833	5.457420597	21.148147404
6	9.732686566	11.938630515	21.419007227
1	10.631433101	12.401805221	21.865678961
1	9.533759232	10.990192386	21.948737483
1	9.955218651	11.692565742	20.366634872
6	10.455251652	15.313096376	18.683487135
1	10.498686970	17.320533802	19.506468285
6	11.944151502	15.263489881	18.390363388
1	12.159665257	14.641599998	17.503730581
1	12.356290662	16.273898889	18.219422132
1	12.500589146	14.817554132	19.238564479
1	10.745692663	8.654557604	19.244518771
6	11.095017271	10.013578368	17.695683303
6	12.519593334	9.992397698	17.778367231
6	13.299058016	10.792763724	16.929372425
1	14.391578560	10.755446083	17.017464342
6	12.697102327	11.637650046	15.970983757
1	13.311437086	12.256158926	15.308877179
6	9.642520001	3.714872524	22.412421630
6	10.889088579	4.295420295	22.093939537
1	11.821234466	3.850562032	22.454967607
6	10.909300622	5.460707715	21.298292963
1	11.867479544	5.926058519	21.037356813
6	9.718374749	6.039020907	20.832083585
6	2.239061422	11.029398456	24.801899351
1	1.796332768	11.118452812	25.801719820
6	2.206226143	13.572496151	24.643990815
1	1.113973862	13.739503780	24.708604801
1	2.611459748	13.680659933	25.667042675
1	2.631507389	14.379851607	24.024138676
6	2.237974093	8.496654198	25.104120637
1	1.520705914	8.698737152	25.918033388
1	3.165667043	8.109359497	25.567741790
1	9.601482047	2.809511340	23.029048284
1	9.746612574	6.947579542	20.219873031
1	13.003636807	9.341768747	18.517267258

^{2}4{BP86}$_{optBS2}$

26	5.763347793	10.315010378	17.758424178
17	7.310912730	8.772064987	18.559179946
7	6.015135505	11.594318783	19.221125512
7	4.246192268	9.516944935	18.731983514
7	5.411612069	9.102457715	16.238698372
7	6.652639539	11.563162700	16.536869976
6	6.998035168	12.586174419	19.261369474
6	7.485984446	12.759235313	20.623155986
6	6.749400321	11.886570513	21.422509826
6	5.771713183	11.245663101	20.552810407
6	4.603963305	10.553330494	20.934215147
6	3.781503737	9.882330688	19.992055774
6	2.453427854	9.307816234	20.213656360
6	2.176961161	8.499825627	19.112620110
6	3.350363045	8.563710864	18.243726450
6	3.706615720	7.647713392	17.223185323
6	4.831756341	7.842507550	16.390572674
6	5.495538106	6.876364657	15.524031802
6	6.441192651	7.588408176	14.787257063
6	6.322016281	8.984239676	15.189276238
6	6.876518896	10.122823747	14.562091709
6	6.827116689	11.402223456	15.161847599
6	7.078385773	12.695903137	14.535341428
6	7.158016415	13.629598203	15.560738312
6	6.987652385	12.894667576	16.810571726
6	7.327162565	13.335010143	18.109885024
6	8.614333398	13.628376651	21.136722989
1	8.616818280	14.605099641	20.629980608
1	8.425217967	13.844376628	22.205835169
6	7.074746000	11.567972233	22.863907947
1	7.725365695	12.366399182	23.265712979
1	6.174158910	11.553356783	23.497137012
6	7.813918913	10.214823256	22.999245127
1	8.103672097	10.038372396	24.051488790
1	7.171040744	9.377154442	22.677005134
1	8.724946050	10.200240794	22.375776897
6	1.473952010	9.617311815	21.324008117
1	0.722102253	8.808621956	21.373189924
1	1.975262976	9.621998006	22.302575224
6	0.743071647	10.963730994	21.120860584
1	0.021487198	11.141519519	21.939822949
1	1.459961767	11.803035218	21.108262487
1	0.189694167	10.980446667	20.164632765
6	0.842995617	7.844446803	18.826420743
1	0.966367158	6.795127384	18.511448116
1	0.252804233	7.816916411	19.761384454
6	0.030212668	8.600706757	17.748802595
1	-0.950861546	8.115768242	17.589823620
1	-0.148480832	9.650533877	18.045591961
1	0.566060453	8.611458263	16.782439216
6	5.344946764	5.370942773	15.486649121
1	4.292981787	5.071588665	15.607078563
1	5.650296835	5.009884252	14.485556354
6	6.202553946	4.650661787	16.554212047
1	6.066768107	3.555625216	16.482273467
1	7.277077674	4.872565097	16.423490227
1	5.919535911	4.964442772	17.574848301
6	7.476721472	6.961297617	13.880798702
1	7.137491712	5.946419001	13.602176219
1	7.576710896	7.520364128	12.937402167
6	8.868210652	6.850370043	14.549658581
1	9.585456100	6.359342520	13.865386502
1	9.267420297	7.845599558	14.809025165
1	8.819341028	6.260294072	15.482131808
6	7.094591461	13.035753465	13.062272304
1	7.601756078	14.009101621	12.926368357
1	7.687773126	12.304252983	12.493292322
6	5.678309768	13.130013632	12.453784183
1	5.731973303	13.396704415	11.381732589
1	5.144759956	12.167359881	12.543285095
1	5.071444630	13.896978345	12.967253211
6	7.229840539	15.127980830	15.367484683
1	7.997099742	15.581529933	16.013997162
1	7.541050330	15.338816754	14.327797439
6	5.875813538	15.825874563	15.628566145
1	5.957791314	16.913208968	15.450062644
1	5.087523822	15.426174854	14.966181387
1	5.546082155	15.675800662	16.671787765
6	4.229933384	10.516649297	22.386883496
6	3.731076258	11.666448820	23.032050300
1	3.616696871	12.597279996	22.460308791
6	3.384663961	11.636015068	24.397598705
6	4.075660475	9.272561751	24.495732318
6	4.394172729	9.325362775	23.125151205
1	4.797308447	8.440943804	22.612747222
6	2.918738256	6.378108459	17.098380194
6	3.047240529	5.377143831	18.089036491
1	3.726362156	5.542050912	18.934696136
6	2.335030486	4.168757686	17.996415847
6	1.471772530	3.984443516	16.894622738
1	0.904119467	3.046657686	16.813322414
6	1.321463303	4.961719522	15.893864764
6	2.059403619	6.159596761	16.005303043
1	1.967603497	6.932116015	15.230520735
6	7.573973985	9.988281978	13.242828725
6	6.867755307	9.718386146	12.053163838
1	5.777102372	9.588494210	12.091842240
6	7.543283103	9.616031947	10.817706510
6	8.944330548	9.767614279	10.801844197
1	9.479462299	9.675985068	9.845437532
6	9.680431348	10.030588933	11.978786573
6	8.977352692	10.145805744	13.190647400
1	9.525378152	10.351628021	14.119668074
6	8.133566699	14.588218932	18.250974911
6	9.489808268	14.589644763	17.864127399
1	9.937422464	13.667167256	17.475321875
6	9.673294739	16.918005628	18.499993855
6	8.319991862	16.946136309	18.891662946
6	7.560145017	15.767489406	18.764696041
1	6.506086858	15.761652295	19.071479330
1	4.448169893	7.129946893	24.633162892
6	2.488733267	3.084910025	19.045782905
1	2.858954240	2.142276107	18.597942545
1	3.199922878	3.382838213	19.834847112
1	1.520381498	2.852440479	19.526889707
6	0.409142205	4.728813546	14.705625716
1	-0.251268619	3.859734915	14.867617099
1	-0.228944647	5.609181370	14.505164626
1	0.991823716	4.536069423	13.784622934
6	6.771149462	9.364519023	9.534288920
1	7.443628358	9.060332092	8.712207622
1	6.010804696	8.570787333	9.664573634
1	6.232241179	10.276005467	9.204407359
6	11.189619673	10.195672660	11.931715701
1	11.476829090	11.129540393	11.410088707
1	11.625094413	10.232825833	12.945865793
1	11.670717774	9.361238231	11.386837534
6	7.708519564	18.209984628	19.463759173
1	7.995687464	19.101714101	18.877282980
1	6.606198221	18.154250697	19.481401528

1	8.048501968	18.384272603	20.503433344
7	10.091280941	9.405283635	19.942947473
1	9.117357347	9.265400012	19.628434616
6	11.316754380	11.078688701	16.854032989
1	10.897333105	11.618758241	15.993569734
6	10.443109036	10.566172198	17.821297799
1	9.358296338	10.691770300	17.714665830
7	5.288621967	7.105005335	20.705980796
1	5.903686848	7.547017255	20.009265040
1	4.298096545	7.278691374	20.521098604
6	4.678194508	5.015934234	21.818348500
1	3.656119795	5.391105878	21.963956890
6	5.624282789	5.826003945	21.137502662
6	10.011177385	12.983941351	21.000396550
1	10.779659077	13.646853751	21.438659957
1	10.060722151	12.006049090	21.508909162
1	10.268148923	12.812309067	19.941348147
6	10.277406948	15.749430574	17.994264804
1	10.276455662	17.832022726	18.597641711
6	11.745008125	15.739214754	17.613997597
1	12.027753505	14.790941941	17.124622115
1	11.990591433	16.570266119	16.925640570
1	12.390137130	15.857223791	18.507532445
1	10.446939802	8.611124665	20.479261764
6	10.951722161	9.859954836	18.942080806
6	12.354159464	9.683186992	19.046549080
6	13.215789818	10.202788187	18.073486285
1	14.298687974	10.052053089	18.180961416
6	12.709952725	10.907682490	16.968408923
1	13.388532933	11.311553135	16.208037020
6	5.044840578	3.757389288	22.315892937
6	6.352169986	3.269726339	22.148080764
1	6.634772042	2.284989538	22.537219922
6	7.290969753	4.067615727	21.470880113
1	8.315387828	3.702569715	21.324532092
6	6.940341910	5.326636167	20.969725375
6	3.568364232	10.433581198	25.112603779
1	3.313036326	10.402921520	26.182412830
6	2.813875825	12.861113583	25.085561215
1	1.714606485	12.784646867	25.196586596
1	3.232806792	12.986041231	26.101347653
1	3.024527020	13.782142806	24.513257929
6	4.300127070	8.001147581	25.294019953
1	3.447880763	7.783780885	25.965435197
1	5.199378184	8.087458648	25.932391416
1	4.296446474	3.151972363	22.843729353
1	7.679173549	5.942412454	20.441344511
1	12.763871220	9.137474020	19.907817201

^{4}4{BP86}$_{optBS1}$

26	5.442604466	10.248235186	17.919821650
17	7.175312389	8.744566786	18.846971295
7	5.632048250	11.507386670	19.397160296
7	3.787194676	9.567944157	18.726238121
7	5.181454663	9.053402714	16.400438737
7	6.497958578	11.445699388	16.782222321
6	6.777923266	12.311208737	19.560659568
6	7.130298275	12.364766474	20.978582332
6	6.111069520	11.702314403	21.678200498
6	5.139123708	11.244858939	20.688222442
6	3.827547054	10.754156612	20.885200007
6	3.118929856	10.119115171	19.836368504
6	1.713788475	9.717150967	19.799756494
6	1.579000429	8.795892089	18.751228197
6	2.898046371	8.649419626	18.138967744
6	3.350874072	7.649395201	17.242567673
6	4.571993411	7.792552390	16.546097511
6	5.301155475	6.808869988	15.748907148
6	6.259260296	7.513395722	15.005063052
6	6.126677110	8.923812017	15.365217719
6	6.693882971	10.061875731	14.749363967
6	6.670322729	11.326972802	15.388927399
6	7.036303152	12.629621424	14.838371196
6	7.230130583	13.494770960	15.925288281
6	6.973411864	12.722243914	17.138310366
6	7.312967501	13.043180874	18.477593927
6	8.410493292	12.881700961	21.596016984
1	8.712446175	13.834094879	21.135024323
1	8.233550809	13.100492195	22.666549365
6	6.136511951	11.392549384	23.158873498
1	6.871003701	12.056909004	23.651134772
1	5.162772211	11.607628509	23.628759355
6	6.522837690	9.918819807	23.460185057
1	6.583852831	9.752218480	24.551542867
1	5.768237863	9.226558237	23.047033612
1	7.499865326	9.667073295	23.010801009
6	0.573404898	10.290848137	20.611555055
1	-0.309638134	9.632617624	20.509245710
1	0.819701417	10.309356481	21.685534714
6	0.181292742	11.724713917	20.163373335
1	-0.673942858	12.096636095	20.756971374
1	1.025114971	12.423567489	20.301007138
1	-0.103518534	11.743910747	19.096107861
6	0.272183757	8.205238695	18.269994917
1	0.381257011	7.130496547	18.052948333
1	-0.478323512	8.280920764	19.079299322
6	-0.281608488	8.918110126	17.007122752
1	-1.246386802	8.473216869	16.701798694
1	-0.440958281	9.995017830	17.195915613
1	0.423474140	8.823408633	16.162425248
6	5.193853393	5.301642247	15.819891213
1	4.144019405	4.972590484	15.752835660
1	5.711747937	4.863885678	14.945802897
6	5.821096204	4.715896037	17.114411838
1	5.760636158	3.612103354	17.106200192
1	6.881623367	5.005928589	17.210663101
1	5.293375399	5.084205813	18.010811018
6	7.319423289	6.875631418	14.134784180
1	6.958670761	5.886406294	13.793879794
1	7.492821191	7.469437159	13.224020306
6	8.671111187	6.687180016	14.876771104
1	9.412031096	6.208759922	14.210292248
1	9.080975470	7.656334075	15.208655027
1	8.548411135	6.052604449	15.771846095
6	7.062383671	13.037544579	13.382661711
1	7.595955780	14.001652271	13.287848753
1	7.631088733	12.311353644	12.780590200
6	5.642526995	13.191604812	12.775126650
1	5.705772426	13.514630153	11.719904543
1	5.094817024	12.233374207	12.809104282
1	5.050695948	13.940366643	13.331806029
6	7.500833672	14.978680081	15.821017438
1	8.270731143	15.288650255	16.544638706
1	7.913114482	15.200900486	14.818894727
6	6.228250650	15.839815348	16.039556989
1	6.464877742	16.914400047	15.934539396
1	5.443588501	15.584434498	15.304789380
1	5.812548406	15.676520577	17.049476417
6	3.186739267	10.882785782	22.235444603
6	2.852739441	12.156073243	22.755364680
1	3.068981934	13.048679132	22.158116837
6	2.247537295	12.288742799	24.024100277
6	2.327315044	9.834600198	24.290734651
6	2.913502746	9.730671231	23.008822604
1	3.166163126	8.745016368	22.602825017
6	2.530660819	6.408432004	17.050853428
6	2.366595546	5.494803925	18.119482423
1	2.837433157	5.713862422	19.083937403
6	1.613319047	4.311594186	17.956884715
6	1.006206252	4.065190674	16.703226224
1	0.408455305	3.155116203	16.568840987
6	1.148323675	4.960991812	15.619092644
6	1.917927129	6.131296598	15.806932639
1	2.044977092	6.839108569	14.980292624
6	7.384114759	9.927344981	13.423084527
6	6.653319171	9.649828673	12.246439110
1	5.565615487	9.530343419	12.305873705
6	7.308136965	9.525705281	10.998285846
6	8.711447465	9.679200878	10.952357211
1	9.227172311	9.585430037	9.988307722
6	9.469732626	9.947224470	12.117645247
6	8.790742936	10.075270216	13.347333397
1	9.360708351	10.277143737	14.260142639
6	8.281410470	14.161989921	18.731481781
6	9.639296228	14.013590773	18.361289249
1	9.974421874	13.076387059	17.904309318
6	10.112337587	16.251557355	19.183130912
6	8.760903152	16.426882478	19.560641968
6	7.853483846	15.368337430	19.333211610
1	6.802609089	15.480119695	19.622425930
1	1.838309935	7.720861189	24.508098164
6	1.477739594	3.312855041	19.093142720
1	2.241729200	2.514678201	19.015935794
1	1.610525804	3.798380679	20.075401200
1	0.488583983	2.820850320	19.084215517
6	0.512515270	4.660227144	14.272771740
1	-0.364862612	3.998750633	14.379225227
1	0.185302212	5.584307371	13.764557860
1	1.228113076	4.151906595	13.597376343
6	6.517538108	9.206610679	9.741150689
1	7.084799283	9.468563351	8.831304065
1	6.277542097	8.127053548	9.682093405
1	5.558545816	9.754483481	9.715812161
6	10.980428812	10.083409037	12.047939356
1	11.284797138	10.822106133	11.283129689
1	11.399848124	10.398480347	13.017602480
1	11.455483543	9.121831127	11.776618033
6	8.304776591	17.719092885	20.215766106
1	8.706624386	18.603145970	19.688195461
1	7.204483235	17.799874609	20.225523871
1	8.652701898	17.781356293	21.265050292
7	10.339926289	8.768077402	18.556727974
1	9.313998797	8.860600431	18.597004452
6	11.294302071	11.359682644	16.065980474
1	10.803003581	12.121780139	15.449056879
6	10.503989896	10.549051828	16.900360891
1	9.415368028	10.672596038	16.930385148
7	7.969732988	6.888571076	21.544457353
1	7.584785882	7.395815321	20.743540163
1	7.646550698	7.150068379	22.469188137
6	9.650851168	5.413113902	22.531635831
1	9.272071991	5.644739589	23.534232906
6	9.036806704	6.018884279	21.398619109
6	9.585019704	11.871870343	21.477885576
1	10.497661069	12.289075306	21.941704650
1	9.344890142	10.918769246	21.981059094
1	9.808889269	11.642343989	20.422303069
6	10.570141717	15.051812763	18.590475819
1	10.825270007	17.067850684	19.356250822
6	12.032720031	14.878643912	18.220067261
1	12.196475007	13.947229807	17.652890726
1	12.396791126	15.724987957	17.608273930
1	12.670149057	14.834571222	19.123995566
1	10.764402502	8.050929588	19.138647197
6	11.108702263	9.553633628	17.726892128
6	12.527318665	9.406664519	17.668247179
6	13.299312031	10.221862758	16.827892879
1	14.386760420	10.085527147	16.806507913
6	12.698248004	11.209900314	16.017297978
1	13.306880635	11.844173292	15.365410852
6	10.728161645	4.527300845	22.373352857
6	11.230221040	4.216769200	21.092609642
1	12.070811054	3.525649787	20.975473019
6	10.625574441	4.811577556	19.965730241
1	10.995300570	4.578193625	18.960545807
6	9.545397113	5.699271503	20.105427721
6	1.997221835	11.118780021	24.779150247
1	1.535212373	11.210706726	25.769998784
6	1.851229096	13.652933896	24.560806725
1	0.772832824	13.848201147	24.402075508
1	2.039399113	13.730247034	25.646663981
1	2.410044155	14.461340982	24.059014865
6	2.086516046	8.595330957	25.135254154
1	1.260544624	8.749032941	25.851648538
1	2.987327154	8.330660433	25.722998231
1	11.181253934	4.074821436	23.262522453
1	9.072680078	6.142766626	19.221856319
1	13.010966657	8.647345449	18.293731566

^{4}4{BP86}$_{optBS2}$

26	5.765683000	10.250823000	17.829991000
17	7.555186000	8.805135000	18.668199000
7	5.970590000	11.519157000	19.313229000
7	4.214448000	9.443000000	18.751480000
7	5.454440000	9.061678000	16.298520000
7	6.710387000	11.513741000	16.637553000
6	6.961359000	12.497902000	19.379460000
6	7.435735000	12.630452000	20.751306000
6	6.674922000	11.754660000	21.526174000
6	5.701771000	11.142207000	20.632075000
6	4.530034000	10.436380000	20.980504000
6	3.720503000	9.796998000	20.006717000
6	2.376087000	9.259746000	20.173183000
6	2.098125000	8.506371000	19.030743000
6	3.292857000	8.550896000	18.197833000
6	3.649114000	7.650105000	17.162972000
6	4.821184000	7.823768000	16.394795000
6	5.500317000	6.852138000	15.546421000
6	6.517078000	7.538250000	14.884855000
6	6.410599000	8.932663000	15.288542000
6	6.996270000	10.060582000	14.677385000
6	6.929052000	11.345861000	15.272104000
6	7.216455000	12.634059000	14.657132000
6	7.261505000	13.571952000	15.688689000
6	7.037074000	12.839328000	16.929493000
6	7.323645000	13.266595000	18.248181000
6	8.597595000	13.442994000	21.277795000
1	8.630550000	14.433579000	20.802492000
1	8.435631000	13.630298000	22.355913000
6	6.962547000	11.430676000	22.975454000
1	7.567989000	12.248105000	23.407842000
1	6.040179000	11.384403000	23.573398000
6	7.738230000	10.102016000	23.142275000
1	7.964128000	9.921986000	24.209448000
1	7.150977000	9.246683000	22.766941000
1	8.691086000	10.121147000	22.585187000
6	1.384299000	9.543445000	21.278366000
1	0.617417000	8.747123000	21.282849000
1	1.870835000	9.500987000	22.263947000
6	0.682437000	10.910528000	21.118633000
1	-0.043947000	11.072095000	21.935648000
1	1.415211000	11.735794000	21.145189000
1	0.139561000	10.972562000	20.158493000
6	0.750037000	7.909773000	18.695501000
1	0.845180000	6.866998000	18.354540000
1	0.134615000	7.878923000	19.613049000
6	-0.004575000	8.725105000	17.619778000
1	-0.999478000	8.283871000	17.425989000
1	-0.148563000	9.773394000	17.938623000
1	0.555621000	8.736597000	16.668123000
6	5.292742000	5.357227000	15.466220000
1	4.224467000	5.103990000	15.514671000
1	5.645565000	5.002175000	14.479687000
6	6.047750000	4.590977000	16.576985000
1	5.846938000	3.506722000	16.503107000
1	7.138095000	4.744845000	16.497088000
1	5.734790000	4.934538000	17.577477000
6	7.607407000	6.880920000	14.069721000
1	7.277992000	5.863069000	13.792370000
1	7.785699000	7.416004000	13.124581000
6	8.941325000	6.776411000	14.847429000
1	9.699868000	6.249918000	14.239282000
1	9.335834000	7.775285000	15.098999000
1	8.809568000	6.223816000	15.794089000
6	7.300047000	12.961341000	13.183880000
1	7.802173000	13.938513000	13.062234000
1	7.926556000	12.229204000	12.652804000
6	5.912091000	13.026418000	12.506340000
1	6.013320000	13.283596000	11.435939000
1	5.392231000	12.054717000	12.576971000
1	5.268625000	13.787357000	12.983648000
6	7.358962000	15.067783000	15.492544000
1	8.081692000	15.519265000	16.189220000
1	7.744295000	15.270223000	14.476710000
6	5.993285000	15.774482000	15.655175000
1	6.093613000	16.859392000	15.468861000
1	5.246162000	15.371010000	14.948088000
1	5.597757000	15.638984000	16.677028000
6	4.128694000	10.357420000	22.422992000
6	3.612090000	11.489686000	23.086399000
1	3.504747000	12.430899000	22.532858000
6	3.243827000	11.430975000	24.443241000
6	3.951140000	9.072411000	24.506384000
6	4.286218000	9.153591000	23.139606000
1	4.694187000	8.271586000	22.630778000
6	2.818726000	6.416748000	16.971095000

6	2.883157000	5.388992000	17.936967000
1	3.559842000	5.514884000	18.791778000
6	2.121370000	4.216038000	17.796806000
6	1.274213000	4.097037000	16.675030000
1	0.662279000	3.191123000	16.561742000
6	1.193397000	5.102840000	15.693873000
6	1.978906000	6.263304000	15.852503000
1	1.934593000	7.060178000	15.098996000
6	7.726215000	9.900577000	13.379675000
6	7.042644000	9.604740000	12.185193000
1	5.952312000	9.480337000	12.207214000
6	7.740686000	9.467260000	10.967406000
6	9.139074000	9.623137000	10.972267000
1	9.690639000	9.517922000	10.027428000
6	9.852703000	9.908327000	12.155340000
6	9.129786000	10.052121000	13.351703000
1	9.661144000	10.284374000	14.282074000
6	8.090974000	14.539250000	18.438345000
6	9.456597000	14.602463000	18.090317000
1	9.951472000	13.709789000	17.689161000
6	9.527257000	16.925944000	18.769885000
6	8.163959000	16.893039000	19.123746000
6	7.457144000	15.686912000	18.956505000
1	6.395955000	15.634865000	19.231473000
1	4.207907000	6.911437000	24.626508000
6	2.222075000	3.096804000	18.815066000
1	2.846799000	2.266162000	18.433376000
1	2.683482000	3.445737000	19.754827000
1	1.229686000	2.671853000	19.052458000
6	0.300537000	4.935982000	14.480155000
1	-0.475631000	4.170138000	14.652197000
1	-0.205407000	5.881534000	14.214461000
1	0.883910000	4.620900000	13.593337000
6	6.997763000	9.136217000	9.687929000
1	7.634295000	9.292830000	8.799683000
1	6.667469000	8.079269000	9.677603000
1	6.091144000	9.758379000	9.571877000
6	11.361883000	10.052776000	12.137613000
1	11.688200000	10.786487000	11.376603000
1	11.746352000	10.381632000	13.118379000
1	11.852899000	9.092316000	11.889798000
6	7.484107000	18.123597000	19.691222000
1	7.731228000	19.029120000	19.107475000
1	6.386104000	18.012652000	19.697651000
1	7.805069000	18.314221000	20.733456000
7	10.652563000	9.497380000	18.986728000
1	9.634146000	9.317398000	18.924013000
6	11.003658000	11.780797000	16.073272000
1	10.356520000	12.416771000	15.455333000
6	10.419544000	11.020217000	17.096148000
1	9.336821000	11.054597000	17.265368000
7	5.283003000	6.746812000	20.104483000
1	6.052654000	7.315742000	19.720756000
1	4.434037000	7.299245000	20.239066000
6	4.568850000	5.317595000	21.953050000
1	3.530289000	5.641039000	21.800254000
6	5.591508000	5.862583000	21.134555000
6	9.966066000	12.747609000	21.093587000
1	10.773625000	13.378615000	21.508256000
1	9.996008000	11.770064000	21.604371000
1	10.185270000	12.565963000	20.027963000
6	10.190978000	15.791710000	18.261617000
1	10.089047000	17.862371000	18.895758000
6	11.667653000	15.844589000	17.922067000
1	11.973208000	14.972843000	17.318959000
1	11.922677000	16.762177000	17.360477000
1	12.287061000	15.844875000	18.839784000
1	11.179442000	8.685682000	19.311362000
6	11.218528000	10.187655000	17.923894000
6	12.613106000	10.138186000	17.666259000
6	13.182028000	10.899051000	16.638254000
1	14.264700000	10.838505000	16.468781000
6	12.389752000	11.734026000	15.829064000
1	12.841193000	12.334435000	15.031345000
6	4.874307000	4.368787000	22.939003000
6	6.197645000	3.942747000	23.142417000
1	6.433201000	3.202990000	23.915652000
6	7.214639000	4.485283000	22.337280000
1	8.255611000	4.170584000	22.484771000
6	6.924156000	5.430969000	21.347228000
6	3.425159000	10.216900000	25.136037000
1	3.154946000	10.163612000	26.200066000
6	2.655995000	12.639025000	25.145333000
1	1.555449000	12.557467000	25.232722000
1	3.054401000	12.744802000	26.170741000
1	2.874932000	13.571325000	24.597243000
6	4.187373000	7.795231000	25.288195000
1	3.407841000	7.641258000	26.056354000
1	5.161834000	7.828122000	25.814776000
1	4.064545000	3.957009000	23.554235000
1	7.724134000	5.848978000	20.724065000
1	13.250809000	9.499807000	18.292476000

^{6}4{BP86}$_{\mathrm{optBS1}}$

26	5.466089822	10.285221644	18.010071112
17	7.257549321	8.899321238	19.086267636
7	5.605980166	11.643708428	19.421284819
7	3.808652915	9.625662862	18.830123462
7	5.214644480	9.005899614	16.550303184
7	6.597437839	11.382320009	16.837731718
6	6.654451162	12.591920814	19.509561461
6	7.059325169	12.724666695	20.913202489
6	6.209787817	11.908851785	21.669775380
6	5.230359061	11.334599208	20.747379474
6	3.988287139	10.733178091	21.030554681
6	3.197471403	10.164501195	19.976499803
6	1.780359885	9.915228737	19.935082986
6	1.531889986	9.136384768	18.768869777
6	2.810316773	8.885874775	18.158838223
6	3.181228966	7.829002450	17.252000532
6	4.444510092	7.820896819	16.647503248
6	5.158924815	6.729714499	15.981492272
6	6.292833622	7.286205674	15.376840987
6	6.256574057	8.726513855	15.641278329
6	6.946542179	9.768126008	14.989797712
6	6.883456177	11.112852999	15.480305514
6	7.191272729	12.348061423	14.802019082
6	7.168130330	13.378048509	15.782987254
6	6.888053012	12.747425191	17.045966024
6	7.138305981	13.255985480	18.373840013
6	8.252664798	13.464562668	21.477561261
1	8.420040372	14.407937303	20.938352042
1	8.037672156	13.744464415	22.526934716
6	6.409527004	11.571668801	23.130932395
1	7.138078833	12.281673880	23.564584866
1	5.476284540	11.695222365	23.705063952
6	6.935238162	10.125834861	23.343715737
1	7.117987512	9.937168073	24.417764459
1	6.203518829	9.383783317	22.981425518
1	7.877569861	9.961457973	22.792620411
6	0.715170461	10.456071897	20.861989740
1	-0.193784608	9.830406738	20.774050985
1	1.036426470	10.378165314	21.913511167
6	0.335881441	11.931173044	20.562182329
1	-0.459801804	12.277801110	21.247648981
1	1.211576485	12.592438260	20.687681593
1	-0.026676444	12.045460135	19.524847094
6	0.162870142	8.774296687	18.235619242
1	0.097207605	7.699332513	17.995183685
1	-0.591179618	8.957831628	19.024235383
6	-0.228427228	9.589809274	16.974277960
1	-1.247485528	9.323747029	16.637488488
1	-0.203092890	10.675140322	17.179153338
1	0.472080910	9.387046190	16.144981185
6	4.850390902	5.250018714	16.055183979
1	3.787864120	5.056781270	15.833019889
1	5.430259707	4.724774612	15.273255922
6	5.195693491	4.624652543	17.433612782
1	5.015195864	3.533817416	17.416479954
1	6.252083160	4.800540852	17.700913582
1	4.567261973	5.063120022	18.228288231
6	7.414237157	6.504723597	14.729683423
1	7.046637864	5.495804491	14.462893033
1	7.730137350	6.975828107	13.785426647
6	8.652816372	6.357650987	15.655136851
1	9.442175413	5.770212496	15.151252076
1	9.069915873	7.346155680	15.913898178
1	8.390258530	5.847258629	16.597981396
6	7.371210976	12.579469989	13.318232960
1	7.947073723	13.512252481	13.164554405
1	7.969317010	11.774506009	12.863690666
6	6.023895643	12.695539617	12.555632636
1	6.198687587	12.889302951	11.481106142
1	5.442685365	11.760995998	12.647520310
1	5.407052506	13.518001418	12.959976416
6	7.291971238	14.858253011	15.494783053
1	8.029208530	15.334398614	16.163433211
1	7.677153991	14.994628639	14.466800736
6	5.942290848	15.613421443	15.623507425
1	6.067277031	16.682588320	15.370164781
1	5.179714769	15.183561395	14.948887143
1	5.554739394	15.547774125	16.655607509
6	3.455413717	10.640352738	22.425115070
6	3.124651939	11.791790545	23.180901499
1	3.274686297	12.781874119	22.737265385
6	2.594153075	11.680818019	24.484748464
6	2.745652645	9.222927315	24.312863514
6	3.249725091	9.363000073	23.001335006
1	3.497850500	8.472268771	22.414063941
6	2.232919190	6.692539204	17.041842254
6	1.838264548	5.873526892	18.129652617
1	2.232063090	6.092799060	19.127941581
6	0.950623718	4.794297731	17.945372146
6	0.435476639	4.555072781	16.647993934
1	-0.267988823	3.726874707	16.496146888
6	0.799301416	5.359343896	15.546576996
6	1.705541265	6.425000706	15.757107134
1	2.001559666	7.064208687	14.917838927
6	7.713367100	9.480837970	13.735122763
6	7.040130576	9.065163362	12.563149944
1	5.950532650	8.951263067	12.586297900
6	7.751448458	8.796217314	11.370820645
6	9.156608200	8.936024541	11.376275873
1	9.718263822	8.722022618	10.458271202
6	9.858655528	9.340929513	12.537111075
6	9.122842793	9.620404432	13.707570993
1	9.648949274	9.930429459	14.615424604
6	8.020042125	14.459022666	18.516825975
6	9.385666649	14.391459425	18.147041332
1	9.789799379	13.450810732	17.759950693
6	9.686739621	16.714194056	18.795857564
6	8.326196285	16.814107601	19.164152577
6	7.501662367	15.674827020	19.021978752
1	6.445407887	15.726725040	19.308669036
1	2.379018202	7.078993873	24.181190855
6	0.563147613	3.892941754	19.104599561
1	1.104434483	2.927967247	19.060170856
1	0.799368446	4.361051546	20.075393479
1	-0.517046936	3.659614780	19.092112329
6	0.248545000	5.079375060	14.159060337
1	-0.656230626	4.448967995	14.204188399
1	-0.012610241	6.014535707	13.632018442
1	0.991306515	4.549088067	13.531971450
6	7.012393935	8.382899105	10.110162748
1	7.701951253	7.958824638	9.360235101
1	6.236640112	7.626206227	10.326939589
1	6.501687673	9.246736913	9.642277015
6	11.373494627	9.447298402	12.526999753
1	11.733701152	10.000315177	11.639857377
1	11.745520066	9.962044018	13.428756648
1	11.843845313	8.445427672	12.497940793
6	7.772324679	18.109574112	19.731800237
1	8.151889035	18.988207881	19.179500846
1	6.669891355	18.129770016	19.688720527
1	8.065398835	18.240013332	20.791849912
7	10.370964113	9.533946114	19.105693947
1	9.340241812	9.457447763	19.057745854
6	11.184286717	11.658444995	16.168874829
1	10.652283054	12.179410213	15.365417344
6	10.440515756	10.922023194	17.106140166
1	9.348962595	10.865004009	17.033265481
7	7.774758853	5.709699810	20.092976609
1	7.605685778	6.648632711	19.716326867
1	6.994230631	5.235099205	20.535144067
6	9.244329193	3.840519148	20.648892217
1	8.403755265	3.309920681	21.111519529
6	9.027085493	5.127756440	20.074988986
6	9.558871432	12.626057252	21.441158517
1	10.401556434	13.208510264	21.857247857
1	9.452544379	11.698422598	22.031062535
1	9.814677476	12.339144313	20.407383822
6	10.233793206	15.510418540	18.291361472
1	10.336689802	17.591494570	18.907331055
6	11.703717393	15.427083559	17.915170649
1	11.981904826	14.406106367	17.603723338
1	11.944873374	16.113839429	17.081082481
1	12.351956124	15.711275087	18.765479092
1	10.838102289	9.073250417	19.881307194
6	11.100160234	10.242773912	18.174385685
6	12.522687727	10.325143663	18.253977575
6	13.248701477	11.063314932	17.308323799
1	14.340062306	11.111512346	17.392490570
6	12.592319683	11.742197361	16.257309507
1	13.164265305	12.316403684	15.522203277
6	10.517809818	3.254855805	20.619722024
6	11.607629469	3.923898802	20.022771898
1	12.598787247	3.461334259	20.001467029
6	11.397897388	5.198354808	19.452046106
1	12.232310063	5.728735828	18.979984218
6	10.132218445	5.801123193	19.473974596
6	2.416512137	10.390728363	25.037249145
1	2.013389877	10.294344918	26.053054904
6	2.197392709	12.916422358	25.273622136
1	1.103077377	13.082091279	25.236110529
1	2.475503437	12.821319211	26.338803947
1	2.682563460	13.823305149	24.873956724
6	2.595148607	7.848851136	24.942389165
1	1.781348907	7.831868139	25.688698263
1	3.524298531	7.543574441	25.462417807
1	10.661520231	2.264833922	21.066020048
1	9.979742146	6.789453998	19.026954121
1	13.044953427	9.806729293	19.066493761

^{6}4{BP86}$_{\mathrm{optBS2}}$

26	5.457078061	10.310601103	17.963246387
17	7.152582446	8.880413923	19.046652799
7	5.608299221	11.655688100	19.390471812
7	3.821783983	9.622422778	18.814116306
7	5.194826381	9.052987260	16.471589039
7	6.600591802	11.414306998	16.796223010
6	6.659506775	12.580011962	19.481050991
6	7.070317025	12.712729695	20.878397743
6	6.230185665	11.893110340	21.628084809
6	5.252622072	11.334907966	20.702226741
6	4.017012763	10.724063293	20.998170892
6	3.221970925	10.138892401	19.963311293
6	1.814012614	9.856576041	19.952166625
6	1.566606999	9.084115183	18.792420193
6	2.839738516	8.878053516	18.163746949
6	3.216919448	7.839241741	17.232914044
6	4.451890215	7.867525950	16.578350039
6	5.154635678	6.788258589	15.884498461
6	6.270535563	7.356678941	15.276443210
6	6.221877981	8.786961903	15.564528103
6	6.930288191	9.831711129	14.937406194
6	6.878361037	11.166923659	15.446378471
6	7.199647016	12.401008670	14.781878733
6	7.181036407	13.412055201	15.770488487
6	6.894341723	12.758731587	17.017249069
6	7.145318464	13.250560972	18.353154347
6	8.251283539	13.467348733	21.445290781
1	8.391416464	14.424869127	20.923742303
1	8.034995309	13.723477446	22.499893561
6	6.440105415	11.543093143	23.083545106
1	7.165303182	12.255752328	23.517305286
1	5.511556777	11.654399886	23.666446505
6	6.981485362	10.106878188	23.276770233
1	7.172110738	9.909221742	24.347891503
1	6.258495493	9.357600551	22.911621916
1	7.923878425	9.957471237	22.721135461
6	0.752540348	10.361143733	20.901640566
1	-0.134789101	9.703831643	20.833014639
1	1.096549065	10.295535184	21.945771907
6	0.319827600	11.816366391	20.606937108
1	-0.472686659	12.139656678	21.307488113
1	1.173913850	12.508905172	20.712483018

1	-0.068870417	11.918444244	19.577645052
6	0.200283962	8.682892054	18.285057974
1	0.157013757	7.605565139	18.051903076
1	-0.543100882	8.851394591	19.086194522
6	-0.228971579	9.480452732	17.032374895
1	-1.248161930	9.192907469	16.713434329
1	-0.222455321	10.568032367	17.228551259
1	0.460121900	9.287717942	16.191264439
6	4.861284760	5.305919412	15.927548119
1	3.792900537	5.107816957	15.751471153
1	5.404890423	4.813657841	15.100391431
6	5.282312001	4.642785098	17.259128936
1	5.111112974	3.551211926	17.215079609
1	6.348847876	4.818249556	17.481573553
1	4.693654830	5.046293059	18.101428737
6	7.388699778	6.590215091	14.607981613
1	7.026062596	5.578996114	14.347044546
1	7.685322914	7.063708751	13.659045438
6	8.636613413	6.455087418	15.512482200
1	9.421705441	5.868967722	15.000443736
1	9.055709446	7.445663788	15.759896940
1	8.393276018	5.948319858	16.462617793
6	7.401406370	12.645318601	13.304066423
1	7.967555146	13.586273495	13.169192039
1	8.020000246	11.853455809	12.854587496
6	6.071452754	12.748937927	12.521680821
1	6.261843834	12.952858621	11.451432644
1	5.498718637	11.807281671	12.594485966
1	5.434728771	13.560447604	12.918030672
6	7.316985019	14.893859246	15.501387464
1	8.039144236	15.362874571	16.189691379
1	7.727061606	15.038234284	14.484678908
6	5.969656267	15.645732048	15.603358652
1	6.099869079	16.717289603	15.362916763
1	5.221874904	15.224517863	14.907026902
1	5.554545996	15.573004017	16.624371050
6	3.501589952	10.658910291	22.395217540
6	3.167494178	11.823899364	23.118804254
1	3.299435040	12.802818696	22.641930663
6	2.664246772	11.746834548	24.430102125
6	2.853017958	9.294039230	24.332160947
6	3.326487187	9.401406516	23.011011142
1	3.578831398	8.496077662	22.445035060
6	2.300384572	6.673149960	17.086873163
6	2.002301509	5.863461673	18.206234330
1	2.450693173	6.115211330	19.175420023
6	1.143284881	4.758403194	18.097974386
6	0.556332149	4.483786455	16.844093252
1	-0.130554100	3.631137729	16.750896749
6	0.823047224	5.274871313	15.712452943
6	1.702918954	6.367991171	15.848271615
1	1.927124293	7.000781416	14.980721395
6	7.738000031	9.560465284	13.710721335
6	7.128506710	9.173750355	12.500770634
1	6.037483001	9.060383064	12.463486867
6	7.899643799	8.929751496	11.345612474
6	9.297430122	9.066417738	11.428162286
1	9.906654036	8.871029839	10.534551155
6	9.937911934	9.442402276	12.627445042
6	9.143001289	9.696745919	13.757192801
1	9.620431855	9.986862746	14.699349068
6	8.022157917	14.451494441	18.500003565
6	9.387418497	14.388513078	18.144222730
1	9.800357547	13.444829865	17.769265376
6	9.665250345	16.713387381	18.763366605
6	8.306215720	16.810230610	19.117517131
6	7.494479190	15.666912806	18.983684954
1	6.433131040	15.715796821	19.258797526
1	2.501542931	7.144031369	24.276810103
6	0.861258018	3.867113350	19.291342691
1	1.427143653	2.917658862	19.225358902
1	1.146857439	4.356764571	20.238405980
1	-0.209445044	3.600544260	19.354876705
6	0.199573450	4.952586313	14.368637360
1	-0.683757252	4.299737032	14.478104293
1	-0.116066632	5.868837296	13.838062122
1	0.917907420	4.428279241	13.708862503
6	7.231156401	8.544019866	10.040755040
1	7.964364114	8.160767246	9.310131273
1	6.463627822	7.763602181	10.193924191
1	6.722151250	9.410822622	9.577406719
6	11.448294647	9.554123039	12.698290267
1	11.846633031	10.176871290	11.875587862
1	11.774843400	9.998560078	13.653899485
1	11.927871369	8.560458509	12.611190993
6	7.738055061	18.107170076	19.660230896
1	8.108628388	18.981394069	19.094730141
1	6.635271959	18.115410757	19.615255853
1	8.027162878	18.260733616	20.718044305
7	10.279158071	9.442029050	19.220333611
1	9.246902427	9.378646877	19.171209837
6	11.099325103	11.574185981	16.307546870
1	10.565142963	12.125604007	15.523700442
6	10.352506475	10.851715012	17.246200348
1	9.257035632	10.830604666	17.191959597
7	7.495215714	5.662982319	20.034673878
1	7.359125142	6.606896241	19.656759283
1	6.700448277	5.210612598	20.476455316
6	8.926144341	3.789343552	20.638638451
1	8.069264404	3.268684499	21.085545695
6	8.733266695	5.069617438	20.053171791
6	9.569906237	12.662173724	21.385229242
1	10.400542026	13.251427617	21.815773026
1	9.488033414	11.716977904	21.950492319
1	9.833114686	12.406980322	20.344877620
6	10.223770821	15.511576890	18.281408284
1	10.308801477	17.598514826	18.867409910
6	11.692525370	15.438794992	17.909307775
1	11.998147726	14.406168395	17.669204026
1	11.914715931	16.070289603	17.027570954
1	12.335003605	15.801673293	18.733471240
1	10.749258023	8.920540533	19.955725182
6	11.007716056	10.134752316	18.287592997
6	12.428764471	10.172157124	18.336445440
6	13.156570653	10.898520968	17.389871058
1	14.251721401	10.908770367	17.455020846
6	12.506790083	11.611574240	16.364015565
1	13.082901711	12.180401971	15.626071005
6	10.191753353	3.195529478	20.644374150
6	11.298658899	3.847942059	20.073010801
1	12.287365317	3.376340846	20.078862513
6	11.112818725	5.115757148	19.491450528
1	11.962862656	5.639393768	19.036725313
6	9.856197213	5.727206290	19.478836922
6	2.520636128	10.475535557	25.022335306
1	2.139587449	10.405103847	26.050719702
6	2.263892391	12.998287233	25.186602778
1	1.167724210	13.152072668	25.157481688
1	2.553368123	12.938051881	26.251427077
1	2.734119153	13.899177180	24.755882583
6	2.733089152	7.940725196	25.005808708
1	1.942430139	7.939555574	25.776965104
1	3.679358228	7.657805485	25.507273065
1	10.315141999	2.206379056	21.102298991
1	9.723032717	6.716867011	19.024921863
1	12.951279323	9.627717024	19.133900447
